# Highly stereocontrolled total synthesis of racemic codonopsinol B through isoxazolidine-4,5-diol vinylation

**DOI:** 10.3762/bjoc.17.188

**Published:** 2021-11-24

**Authors:** Lukáš Ďurina, Anna Ďurinová, František Trejtnar, Ľuboš Janotka, Lucia Messingerová, Jana Doháňošová, Ján Moncol, Róbert Fischer

**Affiliations:** 1 Institute of Organic Chemistry, Catalysis and Petrochemistry, Slovak University of Technology in Bratislava, Radlinského 9, 81237 Bratislava, Slovak Republic; 2 Faculty of Pharmacy in Hradec Kralove, Charles University, Heyrovskeho 1203, 50005 Hradec Kralove, Czech Republic; 3 Institute of Molecular Physiology and Genetics, Centre of Biosciences, Slovak Academy of Sciences, Dúbravská cesta 9, 845 05 Bratislava 4, Slovak Republic; 4 Institute of Biochemistry and Microbiology, Slovak University of Technology in Bratislava, Radlinského 9, 812 37 Bratislava, Slovak Republic; 5 Central Laboratories, Slovak University of Technology in Bratislava, Radlinského 9, 81237 Bratislava, Slovak Republic; 6 Institute of Inorganic Chemistry, Technology and Materials, Slovak University of Technology in Bratislava, Radlinského 9, 81237 Bratislava, Slovak Republic

**Keywords:** alkaloids, antiproliferative effect, codonopsinol B, diastereoselectivity, pyrrolidines

## Abstract

A new highly diastereoselective synthesis of the polyhydroxylated pyrrolidine alkaloid (±)-codonopsinol B and its *N*-nor-methyl analogue, starting from achiral materials, is presented. The strategy relies on the *trans*-stereoselective epoxidation of 2,3-dihydroisoxazole with in situ-generated DMDO, the *syn*-selective α-chelation-controlled addition of vinyl-MgBr/CeCl_3_ to the isoxazolidine-4,5-diol intermediate, and the substrate-directed epoxidation of the terminal double bond of the corresponding γ-amino-α,β-diol with aqueous hydrogen peroxide catalyzed by phosphotungstic heteropoly acid. Each of the key reactions proceeded with an excellent diastereoselectivity (dr > 95:5). (±)-Codonopsinol B was prepared in 10 steps with overall 8.4% yield. The antiproliferative effect of (±)-codonopsinol B and its *N*-nor-methyl analogue was evaluated using several cell line models.

## Introduction

**Figure 1:**
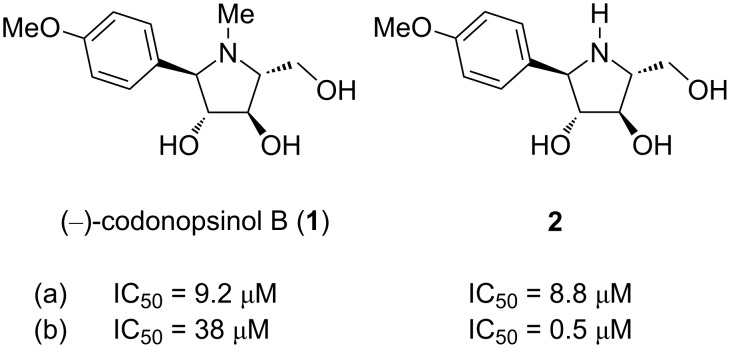
(−)-Codonopsinol B (**1**) and its *N*-nor-methyl analogue **2**; known inhibition activities against α-glucosidases from: (a) *Bacillus stearothermophilus lyoph.*, (b) yeast [2].

Codonopsinol B (**1**) is a polyhydroxylated pyrrolidine alkaloid isolated from the roots of the plant *Codonopsis pilosula* (Figure 1) [1]. This compound was first prepared synthetically before its isolation from natural crude material, employing a stereoselective addition of an aryl Grignard reagent to a five-membered chiral cyclic nitrone derived from ᴅ-arabinose [2]. Its analytical data were consistent with those for the later isolated natural product. Codonopsinol B, together with its *N*-nor-methyl analogue **2**, proved to be a potent α-glucosidase inhibitor (Figure 1).

The potency it showed is higher than that of the known natural alkaloids such as radicamines A and B [3–4], and codonopsinol [5]. In contrast, the unnatural enantiomers of **1** and radicamine A were inactive towards the examined α-glucosidases. The enantiomer of **2** was the only one that still exhibited inhibitory activity against yeast α-glucosidase [2]. To our knowledge, the sole total synthesis of (−)-codonopsinol B and similar hydroxylated pyrrolidines to date was reported in the above mentioned work. Pyrrolidine **2** has been prepared before, however, it was not tested against glycosidases [6]. In addition, a very limited number of related 2-aryl-substituted hydroxylated pyrrolidines with a hydroxymethyl substituent at C-5 have been synthesized [7].

Along with (−)-codonopsinol B, five other pyrrolidine alkaloids, namely codonopsinol A and C, codonopiloside A, codonopyrrolidium B, and radicamine A, have been isolated from *C. pilosula* [1]. Its fresh or dried roots are generally considered as famous herbal medicines and are a part of Radix Codonopsis (together with *C*. *pilosula* var. *modesta* and *C*. *tangshen*). This crude drug is called *Dangshen* in Chinese and *Tojin* in Japanese and has been used as traditional Chinese medicine with numerous beneficial pharmacological activities to treat multiple diseases [8–9], including cancer [10–13]. It is assumed that polyacetylenes, phenylpropanoids, triterpenoids, polysaccharides, and alkaloids are responsible for the majority of the activities found in *Codonopsis* species. Although the polyhydroxylated pyrrolidine alkaloids from *C. pilosula* possess glycosidase inhibitory activities and they are considered to be anticancer species, their activity against human cancer cell lines has never been described.

In view of this, we have developed an efficient and highly diastereoselective approach towards codonopsinol B (**1**) and its *N*-nor-methyl analogue **2** from achiral starting materials and evaluated their anticancer activity using four different cancer cell lines U87-MG, HepG2, JEG-3 and MOLM-13 (AML cell line) as well as immortalized proximal tubular cells HK2. We have very recently found out that even enantiomerically pure polyhydroxylated pyrrolizidine alkaloids with proven antiglycosidase activities may not exhibit antiproliferative effects against cancer cell lines [14]. For this reason, the compounds **1** and **2** were prepared first in their racemic form to begin the initial biological studies.

## Results and Discussion

**Scheme 1:**
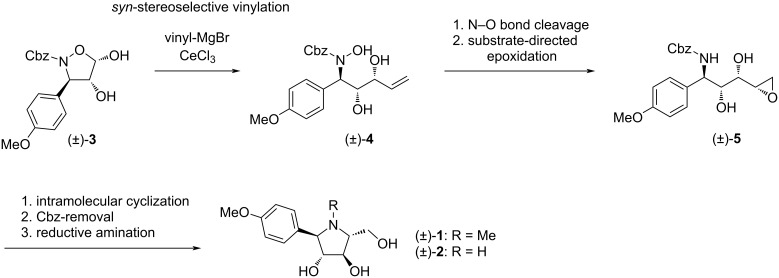
Synthetic approach towards (±)-codonopsinol B (**1**) and its *N*-nor-methyl analogue **2**.

The starting isoxazolidine-4,5-diol **3**, possessing the desired 3,4-*trans* configuration (Scheme 1), will be prepared by using our methodology based on the *trans*-stereoselective epoxidation reaction of 2,3-dihydroisoxazoles followed by the regioselective hydrolysis of the corresponding isoxazolidinyl epoxide [15–16]. Very recently, we have reported the synthesis of γ-(hydroxyamino)-α,β-diols by the addition of Grignard reagents to isoxazolidine-4,5-diols in the presence of anhydrous cerium chloride [17], which proceeded in a highly *syn*-diastereoselective manner due to the presence of the unprotected hydroxy group in the α-position. Accordingly, the diol **3** will be examined in the reaction with vinylmagnesium bromide with an emphasis on the expected high *syn* diol diastereoselectivity (Scheme 1). The obtained *anti*,*syn*-(hydroxyamino)alkenol **4** will be then subjected to reductive cleavage of the N–O bond. Next, a key intermediate epoxide **5** with the desired *syn* (*threo*) configuration between the hydroxy group and the epoxide oxygen could be prepared by substrate-directed epoxidation. A subsequent S_N_2 intramolecular epoxide ring-opening cyclization could provide an *N*-Cbz-protected pyrrolidine derivative with a hydroxymethyl group at C-5 and with *trans* configuration relative to the hydroxy group at C-4. Finally, (±)-codonopsinol B (**1**) will be obtained by treatment of the deprotected pyrrolidine **2** with formaldehyde under reductive amination conditions.

**Scheme 2:**
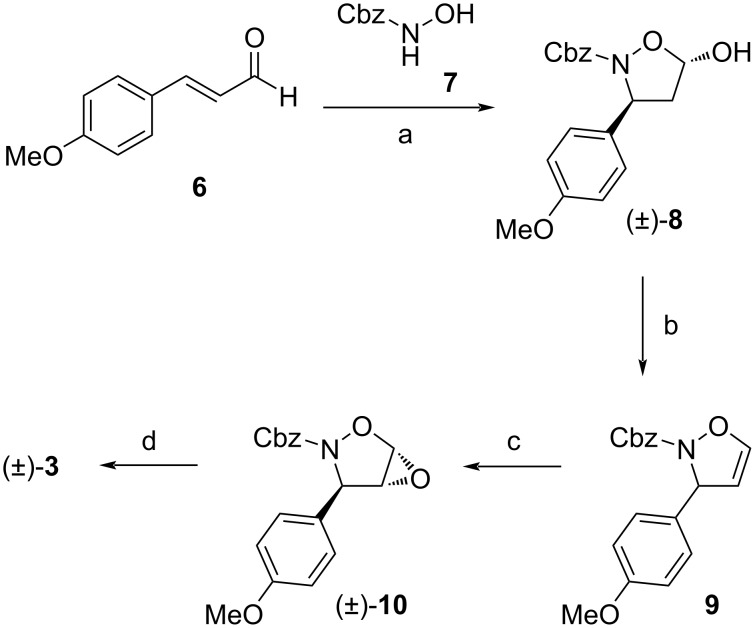
Synthesis of isoxazolidine-4,5-diol (±)-**3**. Reagents and conditions: (a) ᴅʟ-proline, CHCl_3_, rt, 48 h, 77%; (b) Tf_2_O, 2-fluoropyridine, NMP, −20 °C to rt, 16 h, 68%; (c) oxone, NaHCO_3_, acetone/H_2_O 3:2, 0 °C to rt, 80 min, 99%; (d) HCl (37 wt % in H_2_O), acetone/H_2_O 4:1, 0 °C, 30 min, 93%.

As described in Scheme 2, isoxazolidine-4,5-diol **3** was readily synthesized in four steps according to our procedure [16], starting from commercially available (*E*)-4-methoxycinnamaldehyde (**6**). Thus, the reaction of **6** with *N*-Cbz-protected hydroxylamine **7** catalyzed by ᴅʟ-proline in chloroform gave racemic 5-hydroxyisoxazolidine **8** in 77% yield almost as a sole *trans* isomer (dr > 9:1). Its structure was confirmed by comparison with already reported NMR data for the known (3*S*,5*S*)-enantiomer [18]. We have used ᴅʟ-proline for three reasons: first, it provides the target compounds **1** and **2** in their racemic form, second, it is able to catalyze the conjugate addition between *N*-EWG-protected hydroxylamines and enals effectively, and third, based on our experience, the products obtained by the proline catalysis are of higher purity (after purification by silica gel column chromatography), than those synthesized by other methods [16]. Definitely, the effective asymmetric organocatalytic conjugate additions of hydroxylamines to enals using various catalysts provide access to enantiomerically enriched isoxazolidin-5-ols [18–24], which, if needed can be converted into the desired pyrrolidine alkaloids. Treatment of **8** with Tf_2_O in the presence of 2-fluoropyridine in NMP at room temperature afforded 2,3-dihydroisoxazole **9** in good 68% yield. The use of originally reported 2-chloropyridine reduced the product yield slightly to 60% [25]. The subsequent epoxidation of **9** with in situ-generated DMDO (a combination of oxone and NaHCO_3_ in acetone/water) provided isoxazolidinyl epoxide **10** in almost quantitative yield as a sole *trans* isomer (dr > 95:5).

**Scheme 3:**
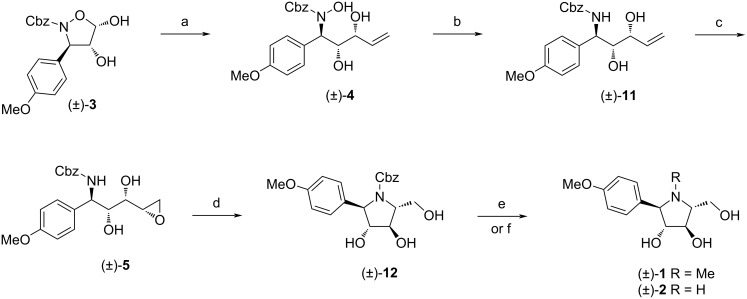
Synthesis of final pyrrolidines (±)-**1** and (±)-**2**. Reagents and conditions: (a) vinyl-MgBr, CeCl_3_, THF, 0 °C to rt, 16 h, 73%; (b) Zn dust, AcOH, 40 °C, 24 h, 85%; (c) 12WO_3_·H_3_PO_4_×H_2_O, H_2_O_2_ (35 wt % in H_2_O), pyridine, ethyl acetate, rt, 48 h, 70%; (d) BF_3_·OEt_2_, CH_2_Cl_2_, 0 °C, 15 min, 69%; (e) H_2_ (1 atm), Pd(OH)_2_/C (5 wt %), MeOH, rt, 2 h, (±)-**2**, 71%; (f) H_2_ (1 atm), Pd(OH)_2_/C (5 wt %), MeOH, rt, 2 h; then formaldehyde (37 wt % in H_2_O), H_2_ (1 atm), Pd(OH)_2_/C (5 wt %), MeOH, rt, 16 h, (±)-**1**, 58% over two steps.

Finally, the acid-catalyzed hydrolysis of **10** with concentrated hydrochloric acid in acetone/water afforded diol **3** in excellent 93% yield. The crystallization of the crude product from a hexanes/CH_2_Cl_2_ mixture led to the preferential formation of the thermodynamically more stable 4,5-*cis* isomer (determined on the basis of a doublet at δ = 5.63 ppm with *J*_4,5_ = 4.1 Hz for the H-5 proton). The reaction of isoxazolidine-4,5-diol **3** with vinylmagnesium bromide in the presence of 1.5 equivalents of anhydrous CeCl_3_ in THF at room temperature proceeded with an excellent *syn* selectivity providing only one *anti*,*syn* isomer of γ-(hydroxyamino)-α,β-diol **4** (dr > 95:5) in 73% yield (Scheme 3). Its relative configuration was determined by comparison with already reported NMR data of related γ-(hydroxyamino)-α,β-diols [17]. More specifically, the value of the vicinal coupling constant *J*_2,3_ = 1.8 Hz and the chemical shift of the H-2 proton at 4.22 ppm referred to the 2,3-*syn* configuration. Whereas the above mentioned addition was highly diastereoselective, the same reaction under identical conditions but in the absence of CeCl_3_ resulted in the formation of a small amount of the *anti*,*anti* diastereomer of **4** (*anti*,*syn*/*anti*,*anti*, 80:20). Subsequently, the treatment of *anti*,*syn*-**4** with zinc in acetic acid at 40 °C gave the *N*-Cbz-protected amino diol **11** in a very good yield of 85% [20,26].

Next, we investigated the substrate-directed epoxidation that could deliver an oxygen atom from the same side of the double bond as the adjacent hydroxy group [27]. First, the epoxidation of **11** with *m*-CPBA in dichloromethane was carried out. However, the reaction was almost nonselective regardless to the reaction temperature, affording a mixture of both *syn* and *anti* isomers of epoxide **5** in comparable amounts (*syn*/*anti*, 58:42; see Supporting Information File 1, page S25). Although the *syn* selectivity was further improved (80:20) by using the in situ-generated trifluoroperoxyacetic acid [28], the reaction suffered from formation of a high level of impurities. Gratifyingly, this issue has been overcome by the use of hydrogen peroxide in the presence of phosphotungstic heteropoly acid, a commercial heteropoly acid [29–30]. Under our optimized conditions in terms of amount of catalyst, reaction temperature, and organic co-solvent, the epoxidation of **11** with aqueous H_2_O_2_ (35 wt %) and 12WO_3_·H_3_PO_4_×H_2_O (1 mol %) at room temperature in ethyl acetate provided the desired epoxide **5** in an acceptable 70% yield with excellent stereoselectivity as the sole *syn* isomer (dr > 95:5). It is worth noting that a small quantity of pyridine was added to prevent unwanted acid-catalyzed epoxide hydrolysis [31]. The stereochemistry of **5** was assigned later after pyrrolidine ring formation. Since the aqueous tungstic acid-catalyzed hydrogen peroxide epoxidations of monosubstituted allylic alcohols usually proceed in *anti* (*erythro*) stereoselective fashion [32], we propose that the high *syn* selectivity can be attributed to the presence of the unprotected hydroxy group in the homoallylic position that assists the electrophilic attack on the double bond [33–34]. The protection of the homoallylic hydroxy group in similar alkenyl diols in epoxidations with the VO(acac)_2_/*t-*BuOOH system led to the formation of the *erythro* isomer [35]. Actually, highly stereoselective epoxidations of terminal alkenes bearing both an allylic and homoallylic-type hydroxy group, yielding a sole *threo* isomer, are rare [36].

**Figure 2:**
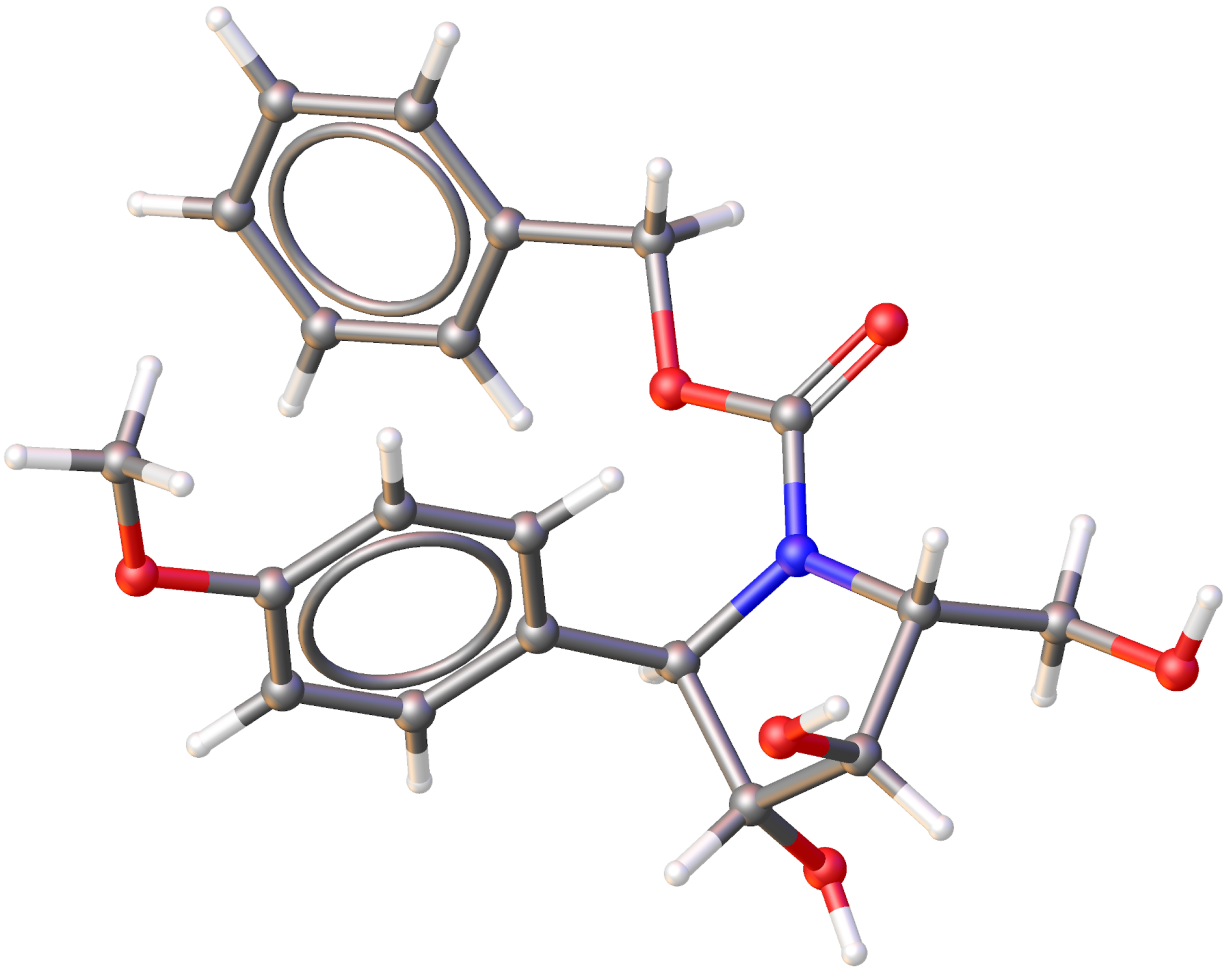
Molecular structure of *N*-Cbz-protected pyrrolidine **12** confirmed by single-crystal X-ray crystallographic analysis, proving the same relative configuration as that in natural codonopsinol B.

The cyclization of **5** through an epoxide ring-opening reaction with boron trifluoride etherate in dichloromethane at 0 °C yielded pyrrolidine derivative **12** in 69% isolated yield [37]. Its structure was determined on the basis of ^1^H and ^13^C NMR spectra. The relative configuration was unambiguously confirmed by X-ray crystallographic analysis (Figure 2) [38] (see also Supporting Information File 1, Figures S1–S3).

Interestingly, NMR spectroscopy of **12** revealed that it exists as a mixture of two rotamers in a ≈2:1 ratio at 25 °C, probably caused by the Cbz-protecting group (see Supporting Information File 1, page S26).

It is worth mentioning that the attempts to prepare polyhydroxylated pyrrolidine **2** directly from epoxide **5** by the one pot Cbz-removal/aminocyclization under hydrogenolysis conditions led only to the formation of several undesired byproducts. To our satisfaction when compound **12** was subjected to catalytic hydrogenation using Pd(OH)_2_/C in methanol [39], **2** was formed in 71% yield.

Finally, (±)-codonopsinol B (**1**) was directly obtained from **12** in the yield of 58% (over two steps) under the same reaction conditions when using formaldehyde (37 wt % in H_2_O). The ^1^H and ^13^C NMR spectra recorded on compounds (±)-**1** and (±)-**2** were in good agreement with those previously reported for the natural sample [1] as well as for the synthetic products [2].

### Biological evaluation

(±)-Codonopsinol B (**1**) and its *N*-nor-methyl analogue **2** were evaluated for their antiproliferative activity against the four cancer cell lines U87-MG, HepG2, JEG-3, MOLM-13 and against immortalized proximal tubular cells HK2 (see Supporting Information File 1, Table S1, Figures S4–S8). The IC_50_ values of all tested compounds exceeded the highest treated concentration (1000 µM respectively and 500 µM for MOLM-13) or were not defined by the employed method of analysis (see Supporting Information File 1). The findings reveal that these compounds display no antiproliferative activity at the tested concentrations.

The results may indicate that the expected antiglycosidase activities of the tested compounds are not sufficient enough to be effective in the examined cancer cell lines. The observed weak or even no antiproliferative activities may be caused due to the high hydrophilicity of the polyhydroxylated pyrrolidine core. It is proven that the presence of lipophilic groups in the structure of such compounds improves their penetration trough the cell membrane, and thereby increases their antitumor effectiveness [40].

## Conclusion

In summary, we have developed an efficient highly diastereoselective synthesis of racemic codonopsinol B (**1**) and its *N*-nor-methyl analogue **2** starting from achiral materials. Four consecutive stereocenters in the target molecules were accomplished sequentially by the organocatalytic aza-Michael addition of *N*-Cbz-protected hydroxylamine to (*E*)-4-methoxycinnamaldehyde, the *trans*-stereoselective epoxidation of 2,3-dihydroisoxazole **9** with in situ-generated DMDO, the *syn*-selective α-chelation-controlled addition of vinylmagnesium bromide to isoxazolidine-4,5-diol **3** in the presence of cerium chloride, and the substrate-directed epoxidation of the terminal double bond of *N*-Cbz-protected γ-amino-α,β-diol **11** with aqueous hydrogen peroxide catalyzed by phosphotungstic heteropoly acid. Each of the key reactions proceeded with an excellent diastereoselectivity (dr > 95:5). Employing our synthetic strategy, racemic codonopsinol B was prepared in 10 steps with overall 8.4% yield.

Although naturally occurring (−)-codonopsinol B (**1**) and its *N*-nor-methyl analogue **2** were found to be effective α-glucosidase inhibitors, their racemic forms showed no evident antiproliferative activities against the selected human cancer cell lines U87-MG, HepG2, JEG-3 and MOLM-13 as well as immortalized proximal tubular cells HK2.

## Supporting Information

File 1 Detailed experimental procedures, characterization data and NMR spectra of synthesized compounds, X-ray crystallographic data of **12**, and biological evaluation of antiproliferative activities of **1** and **2**.
