# Supplementary material for: Highly stereocontrolled total synthesis of racemic codonopsinol B through isoxazolidine-4,5-diol vinylation
Source: Beilstein J Org Chem. 2021 Nov 24;17:2781–6. doi: 10.3762/bjoc.17.188 (PMC8630432; doi:10.3762/bjoc.17.188)
Supplement: File 1 — Detailed experimental procedures, characterization data and NMR spectra of synthesized compounds, X-ray crystallographic data of 12, and biological evaluation of antiproliferative activities of 1 and 2. [file Beilstein_J_Org_Chem-17-2781-s001.pdf]

BEILSTEIN JOURNAL OF ORGANIC CHEMISTRY

# Supporting Information

for

# Highly stereocontrolled total synthesis of racemic codonopsinol B through isoxazolidine-4,5-diol vinylation

Lukáš Ďurina, Anna Ďurinová, František Trejtnar, Ľuboš Janotka, Lucia Messingerová, Jana Doháňošová, Ján Moncol and Róbert Fischer

*Beilstein J. Org. Chem.* **2021,** *17,* 2781–2786. doi:10.3762/bjoc.17.188

# Detailed experimental procedures, characterization data and NMR spectra of synthesized compounds, X-ray crystallographic data of 12, and biological evaluation of antiproliferative activities of 1 and 2

License and Terms: This is a supporting information file under the terms of the Creative Commons Attribution License (https://creativecommons.org/licenses/by/4.0). Please note that the reuse, redistribution and reproduction in particular requires that the author(s) and source are credited and that individual graphics may be subject to special legal provisions.
The license is subject to the *Beilstein Journal of Organic Chemistry* terms and conditions: (https://www.beilstein-journals.org/bjoc/terms)

# Table of contents


| | | |
|---|---|---|
| 1. | Experimental section | S2–S10 |
| 2. | General remarks | S2–S8 |
| 3. | Experimental procedures | S2 |
| 4. | Crystallography | S8, S9 |
| 5. | Cell culture and cultivation conditions | S9 |
| 6. | Cell viability testing | S9, S10 |
| 7. | $^{1}$H, $^{13}$C NMR, APT spectra of compound **9** | S11, S12 |
| 8. | $^{1}$H, $^{13}$C NMR, APT spectra of compound (±)-**10** | S13, S14 |
| 9. | $^{1}$H, $^{13}$C NMR, APT spectra of compound (±)-**3** | S15, S16 |
| 10. | $^{1}$H, $^{13}$C NMR, APT spectra of compound (±)-*anti*,*syn*-**4** | S17, S18 |
| 11. | $^{1}$H, $^{13}$C NMR, APT spectra of compound (±)-*anti*,*anti*-**4** | S19, S20 |
| 12. | $^{1}$H, $^{13}$C NMR, APT spectra of compound (±)-**11** | S21, S22 |
| 13. | $^{1}$H, $^{13}$C NMR, APT spectra of compound (±)-**5** | S23, S24 |
| 14. | The part of $^{1}$H NMR spectrum of the crude product (±)-**5** obtained from an epoxidation of (±)-**11** with $H_2O_2/12WO_3 \cdot H_3PO_4 \times H_2O$ (spectrum A) and with *m*-CPBA (spectrum B) | S25 |
| 15. | $^{1}$H and $^{13}$C NMR spectra of compound (±)-**12** | S26 |
| 16. | $^{1}$H, $^{13}$C NMR, APT spectra of compound (±)-**2** | S27, S28 |
| 17. | $^{1}$H and $^{13}$C NMR spectra of (±)-codonopsinol B (**1**) | S29 |
| 18. | The ORTEP-like style drawn of IAM model of compound (±)-**12** | S30 |
| 19. | The ORTEP-like style drawn of HAR model of compound (±)-**12** | S31 |
| 20. | The O–H···O hydrogen bonds in crystal structure of compound (±)-**12** | S32 |
| 21. | Evaluation of antiproliferative effect: Summarization of $IC_{50}$ values for compounds (±)-**1** and (±)-**2** | S33 |
| 22. | Antiproliferative effect of the tested compounds (±)-**1** and (±)-**2** on U87-MG cells | S34 |
| 23. | Antiproliferative effect of the tested compounds (±)-**1** and (±)-**2** on HK-2 cells | S35 |
| 24. | Antiproliferative effect of the tested compounds (±)-**1** and (±)-**2** on HepG2 cells | S36 |
| 25. | Antiproliferative effect of the tested compounds (±)-**1** and (±)-**2** on JEG-3 cells | S37 |
| 26. | Antiproliferative effect of the tested compounds (±)-**1** and (±)-**2** on MOLM-13 cells | S38 |
| 27. | References | S39 |

# Experimental section

## General remarks

Flash column chromatography (FCC) was carried out with a Büchi system (Pump Manager C-615 and Fraction Collector C-660) using Normasil 60 silica gel (0.040–0.063 mm; VWR). Thin layer chromatography (TLC) analysis was carried out using TLC silica gel 60 F254 (aluminium sheets, Merck), and plates were visualized with UV light or by treatment with permanganate solution followed by heating. Melting points were obtained using a Melting Point B-540 (Büchi) instrument. Infrared (IR) spectra were recorded as neat samples with a Nicolet 5700 FTIR spectrometer with an ATR Smart Orbit Diamond adapter (Thermo Electron Corporation). NMR spectra were recorded with a Varian INOVA-300 spectrometer ($^1$H, 299.95 MHz and $^{13}$C, 75.42 MHz) and a Varian VNMRS-600 instrument ($^1$H, 599.75 MHz and $^{13}$C, 150.81 MHz) in $CDCl_3$ (using tetramethylsilane as the internal standard), $CD_3OD$ (residual [$D_4$]methanol, $\delta_H$ = 3.31, 4.87 ppm, $\delta_C$ = 49.00 ppm), and $D_2O$. Chemical shifts are reported in parts per million (ppm). HRMS analysis was carried out with an Orbitrap Velos Pro spectrometer (Thermo Fisher Scientific). All solvents used were dried and distilled according to conventional methods. Oxone®, monopersulfate ($2KHSO_5 \cdot KHSO_4 \cdot K_2SO_4$, MW: 615.50) and cerium(III) chloride (anhydrous, 99.5%) were purchased from Alfa Aesar. Phosphotungstic acid hydrate ($12WO_3 \cdot H_3PO_4 \times H_2O$, MW: 2880.05 (anhydrous basis)) was purchased from Sigma-Aldrich. 4-Methoxycinnamaldehyde (**6**) was purchased from Acros Organics. *N*-Cbz-Hydroxylamine **7** was prepared using a published procedure [1].

## Experimental procedures

### (±)-Benzyl 5-hydroxy-3-(4-methoxyphenyl)isoxazolidine-2-carboxylate (8)

4-Methoxycinnamaldehyde (**6**, 120 mg, 0.74 mmol) and *N*-Cbz-hydroxylamine **7** (100 mg, 0.60 mmol) were added to a stirred solution of DL-proline (13.8 mg, 0.12 mmol) in anhydrous $CHCl_3$ (1.2 mL) at rt. The mixture was stirred for 48 h. After this time, TLC showed that the reaction was complete (hexanes/EtOAc 2:1). The solvent was evaporated under reduced pressure and the product was purified by FCC (hexanes/EtOAc, 85:15) to give isoxazolidine **8** (150 mg, 0.46 mmol, 77%) as a white solid with spectroscopic data in good agreement with those reported in the literature [2]. mp 91–93 °C; $R_f$ = 0.16 (*n*-hexane/EtOAc, 2:1); $^1$H NMR (300 MHz, $CDCl_3$): δ = 7.30–7.22 (m, 7 H, Ph-H), 6.88–6.83 (m, 2 H, Ph-H), 5.84 (dd, *J* = 4.7, 2.7 Hz, 1 H, 5-H), 5.34 (bs, 1 H, OH), 5.33 (t, *J* = 8.2 Hz, 1 H, 3-H), 5.17 (s, 2 H, OC$H_2$Ph), 3.79 (s, 3 H, OMe), 2.74 (dd, *J* = 12.6, 8.4 Hz, 1 H, 4-H), 2.33–2.24 (m, 1 H, 4-H); $^{13}$C NMR (75 MHz, $CDCl_3$): δ = 159.4, 159.1 (C=O, C-Ph), 135.9 (C-Ph), 133.6 (C-Ph), 128.6 (CH-Ph), 128.3 (CH-Ph), 127.9 (CH-Ph), 127.5 (CH-Ph), 114.2 (CH-Ph), 98.9 (C-5), 68.2 ($CH_2$Ph), 61.0 (C-3), 55.5 (OMe), 45.4 (C-4).

**(±)-Benzyl 3-(4-methoxyphenyl)isoxazole-2(3*H*)-carboxylate (9)**

Hydroxyisoxazolidine **8** (1.0 g, 3.04 mmol) was placed in a reaction flask, which was subsequently sealed with a rubber septum, evacuated, and filled with argon. Anhydrous NMP (30 mL) was added, and the resulting solution was cooled in ice/NaCl bath (−20 °C). 2-Fluoropyridine (1.83 mL, 21.3 mmol) and $Tf_2O$ (0.76 mL, 4.52 mmol, 1.5 equiv) were added. The mixture was stirred at −20 °C for 20 min, and then allowed to warm to rt within 16 h. After this time, TLC showed that the reaction was complete (hexanes/EtOAc 2:1). The mixture was poured into $H_2O$ (100 mL), and sat. solution of $NaHCO_3$ (10 mL) was added. The mixture was subsequently extracted with $Et_2O$ (3 × 100 mL). The combined organic layers were washed with $H_2O$ (2 × 100 mL), dried ($MgSO_4$), and concentrated in vacuo. The product was purified by FCC (hexanes/EtOAc 9:1) to give 2,3-dihydroisoxazole **9** (640 mg, 2.06 mmol, 68%) as a white solid which gradually became yellow over time. mp 77–79 °C; $R_f$ = 0.29 (*n*-hexane/EtOAc, 7:3); $^1$H NMR (300 MHz, $CDCl_3$): δ = 7.35–7.30 (m, 5 H, H-Ph), 7.30–7.25 (m, 2 H, H-Ph), 6.89–6.84 (m, 2 H, H-Ph), 6.68 (dd, *J* = 3.1, 2.1 Hz, 1 H, 5-H), 5.90 (pseudo t, *J* = 2.3, 2.2 Hz, 1 H, 3-H), 5.25 (d, *J* = 12.2 Hz, 1 H, OC$H_2$Ph), 5.19 (d, *J* = 12.2 Hz, 1 H, OC$H_2$Ph), 5.15 (dd, *J* = 3.1, 2.4 Hz, 1 H, 4-H), 3.79 (s, 3 H, $OCH_3$); $^{13}$C NMR (75 MHz, $CDCl_3$): δ =159.6, 156.8 (C=O, C-Ph), 141.6 (C-5), 135.5 (C-Ph), 132.2 (C-Ph), 128.5 (CH-Ph), 128.3 (× 2, CH-Ph), 128.2 (CH-Ph), 114.0 (CH-Ph), 101.9 (C-4), 68.2 ($CH_2$Ph), 66.9 (C-3), 55.3 ($OCH_3$); IR (ATR): $\nu_{max}$ = 3105, 2837, 1703, 1342, 1238, 1154, 1073, 1031, 823, 776, 730, 694, 561 cm$^{-1}$. HRMS (ESI): *m/z* calcd for $C_{18}H_{18}NO_4$: 312.1231 [M+H$^+$]; found: 312.1232.

**(±)-Benzyl 4-(4-methoxyphenyl)-2,6-dioxa-3-azabicyclo[3.1.0]hexane-3-carboxylate (10)**

Solid $NaHCO_3$ (2.0 g; 23.8 mmol) was placed in a reaction flask, and $H_2O$ (13 mL) followed by acetone (21 mL) were added. The resulting mixture was cooled to 0 °C in an ice bath and stirred for 20 min. Oxone (2.09 g, 3.40 mmol) was added in one portion, and stirring was continued at 0 °C for further 15 min. Then, 2,3-dihydroizoxazole **9** (530 mg, 1.70 mmol) was added in one portion. The ice bath was removed, and the reaction mixture was stirred at rt for an additional 80 min. After this time, TLC showed that the reaction was complete (hexanes/EtOAc 4:1). The reaction mixture was diluted with $H_2O$ (40 mL) and extracted with $CH_2Cl_2$ (3 × 30 mL). The organic layers were dried ($MgSO_4$), and the solvent was evaporated in vacuo to give isoxazolidinyl epoxide **10** (550 mg, 1.68 mol, 99%) as a white solid with satisfactory purity. mp 83–85 °C; $R_f$ = 0.24 (*n*-hexane/EtOAc, 7:3); $^1$H NMR (600 MHz, $CDCl_3$, 25 °C): δ = 7.40–7.30 (m, 5 H, H-Ph), 7.28–7.23 (m, 2 H, H-Ph), 6.90–6.88 (m, 2 H, H-Ph), 5.59 (bs, 1 H, 3-H), 5.56 (bs, 1 H, 5-H), 5.26 (d, *J* = 12.3 Hz, 1 H, OC$H_2$Ph), 5.22 (d, *J* = 12.5 Hz, 1 H, OC$H_2$Ph), 3.86 (d, *J* = 1.7 Hz, 1 H, 4-H), 3.79 (s, 3 H; $OCH_3$); $^{13}$C NMR (150 MHz, $CDCl_3$, 25 °C): δ = 159.8 (C-Ph), 135.5 (C-Ph), 128.5 (CH-Ph), 128.3 (CH-Ph), 128.1 (CH-Ph), 128.0 (CH-Ph), 126.7 (C-Ph), 114.4 (CH-Ph), 80.4 (C-5), 68.6 ($CH_2$Ph), 63.7 (C-3), 60.0

(C-4), 55.3 ($OCH_3$), (the signal corresponding to the carbonyl carbon is missing); IR (ATR): $\nu_{max}$ = 2960, 2835, 1715, 1514, 1306, 1285, 1234, 1178, 1031, 830, 809, 728, 696, 506 $cm^{-1}$.

**(±)-Benzyl 4,5-dihydroxy-3-(4-methoxyphenyl)isoxazolidine-2-carboxylate (3)**

To a stirred solution of epoxide **10** (650 mg, 1.99 mol) in acetone/water 4:1 (v/v, 40 mL) at 0 °C was added concentrated HCl (37 wt % in $H_2O$, 3 drops). The reaction mixture was stirred for 30 min. After this time, TLC showed that the reaction was complete (hexanes/EtOAc 1:1). The mixture was diluted with water (40 mL) and extracted with $CH_2Cl_2$ (3 × 30 mL). The combined organic layers were diluted with $MgSO_4$, and the solvent was evaporated under reduced pressure. The residue was crystallized from hexanes/$CH_2Cl_2$ to give single 4,5-*cis*-anomer **3** (640 mg, 1.85 mmol, 93%) as a white solid. mp 143–144 °C; $R_f$ = 0.14 (*n*-hexane/EtOAc, 1:1); $^1$H NMR (300 MHz, $CDCl_3$): δ = 7.32–7.19 (m, 7 H, H-Ph), 6.92–6.87 (m, 2 H, H-Ph), 6.39 (bs, 1 H, 5-OH), 5.63 (pseudo t, *J* = 4.1 Hz, 1 H, 5-H), 5.19 (s, 2 H, OC$H_2$Ph), 4.84 (d, *J* = 5.7 Hz, 1 H, 3-H), 4.40–4.30 (m, 1 H, 4-H), 3.81 (s, 3 H, $OCH_3$), 2.95 (d, *J* = 10.1 Hz, 1 H, 4-OH); $^1$H NMR (300 MHz, $CDCl_3$ + $D_2O$): δ = 7.32–7.20 (m, 7 H, H-Ph), 6.92–6.87 (m, 2 H, H-Ph), 5.60 (d, *J* = 4.3 Hz, 1 H, 5-H), 5.18 (s, 2 H, OC$H_2$Ph), 4.84 (d, *J* = 5.7 Hz, 1 H, 3-H), 4.33 (dd, *J* = 5.7, 4.3 Hz, 1 H, 4-H), 3.81 (s, 3 H, $OCH_3$); $^{13}$C NMR (150 MHz, $CDCl_3$): δ = 159.3 (C-Ph), 135.3 (C-Ph), 131.1 (C-Ph), 128.5 (CH-Ph), 128.2 (CH-Ph), 127.6 (CH-Ph), 127.2 (CH-Ph), 114.1 (CH-Ph), 96.0 (C-5), 82.2 (C-4), 68.4 (O*C*$H_2$Ph), 67.6 (C-3), 55.3 ($OCH_3$), (the signal corresponding to the carbonyl carbon is missing); IR (ATR): $\nu_{max}$ = 3350, 2954, 1704, 1513, 1302, 1244, 1141, 1065, 1028, 801, 737, 696, 554 $cm^{-1}$; HRMS (ESI): *m/z* calcd for $C_{18}H_{19}NNaO_6$: 368.1105 $[M + Na]^+$; found: 368.1105.

**(±)-Benzyl [2,3-dihydroxy-1-(4-methoxyphenyl)pent-4-en-1-yl](hydroxy)carbamate (4)**

In a manner similar to [3], a reaction flask containing diol **3** (760 mg, 2.20 mmol) and anhydrous $CeCl_3$ (815 mg, 3.31 mmol) was sealed with a rubber septum, evacuated, and filled with argon. Anhydrous THF (22 mL) was added, and the stirred mixture was cooled to 0 °C. Vinyl-MgBr (8.83 mL, 8.83 mmol, 1 M solution in THF) was added dropwise over 5 min. The reaction mixture was allowed to slowly warm to rt over 16 h. When TLC showed that the reaction was complete [$CH_2Cl_2$/acetone/$NH_3$ 4:1:0.3 (aq., 26%)], the reaction was quenched with 1 M HCl (100 mL), and the product was extracted into $Et_2O$ (3 × 100 mL). The combined organic layers were dried over $MgSO_4$, and the solvent was evaporated under reduced pressure. The residue was purified by FCC ($CH_2Cl_2$/acetone 9:1) to give single diastereoisomer *anti*,*syn*-**4** (600 mg, 1.61 mmol, 73%) as a pale yellow sticky oil which turned to a solid in the refrigerator. mp 100–101 °C (recrystallized from hexanes/$CH_2Cl_2$); $R_f$ = 0.28 [$CH_2Cl_2$/acetone/$NH_3$ (aq., 26%), 4:1:0.3]; $^1$H NMR (600 MHz, $CD_3OD$): δ = 7.39–7.28 (m, 7 H, H-Ph), 6.87–6.82 (m, 2 H, H-Ph), 6.05 (ddd, *J* = 17.3, 10.5, 5.8 Hz, 1 H, 4-

H), 5.34 (dt, $J$ = 17.3, 1.7 Hz, 1 H, 5-$H_a$), 5.21 (d, $J$ = 9.9 Hz, 1 H, 1-H), 5.18 (d, $J$ = 12.4 Hz, 1 H, OC$H_2$Ph), 5.17 (td, $J$ = 10.5, 1.7 Hz, 1 H, 5-$H_b$), 5.09 (d, $J$ = 12.4 Hz, 1 H, OC$H_2$Ph), 4.34 (dq, $J$ = 5.8, 1.6 Hz, 1 H, 3-H), 4.22 (dd, $J$ = 9.9, 1.8 Hz, 1H, 2-H), 3.77 (s, 3 H, OMe); $^{13}$C NMR (75 MHz, $CD_3OD$): δ = 160.6, 158.8 (C=O, C-Ph), 140.3 (C-4), 137.8 (C-Ph), 131.5 (C-Ph), 131.4 (CH-Ph), 129.4 (CH-Ph), 129.1 (CH-Ph), 129.0 (CH-Ph), 115.9 (C-5), 114.4 (CH-Ph), 73.5 (C-2), 73.1 (C-3), 68.7 (O$CH_2$Ph), 63.8 (C-1), 55.7 ($OCH_3$); IR (ATR): $\nu_{max}$ = 3325, 2935, 1689, 1513, 1407, 1247, 1178, 1105, 1028, 745, 696, 561 cm$^{-1}$; HRMS (ESI): *m/z* calcd for $C_{20}H_{24}NO_6$: 374.1599 [M + H]$^+$; found: 374.1610; *m/z* calcd for $C_{20}H_{23}NNaO_6$: 396.1418 [M + Na]$^+$; found: 396.1429.

Data for γ-(hydroxyamino)-α,β-diol *anti*,*anti*-**4** isolated in a small amount (≈ 10%) when the reaction was performed in the absence of anhydrous $CeCl_3$: Pale yellow sticky oil. $R_f$ = 0.05 (*n*-hexane/EtOAc, 55:45); $R_f$ = 0.11 [$CH_2Cl_2$/acetone/$NH_3$ (aq., 26%), 4:1:0.3]; $^1$H NMR (300 MHz, $CD_3OD$): δ = 7.39–7.27 (m, 7 H, H-Ph), 6.87–6.82 (m, 2 H, H-Ph), 6.02 (ddd, $J$ = 17.4, 10.5, 7.0 Hz, 1 H, 4-H), 5.26–5.16 (m, 2 H, 5-H), 5.14 (d, $J$ = 12.3 Hz, 1 H, OC$H_2$Ph), 5.08 (d, $J$ = 12.3 Hz, 1 H, OC$H_2$Ph), 4.92 (d, $J$ = 9.5 Hz, 1 H, 1-H), 4.37 (dd, $J$ = 9.5, 3.5 Hz, 1 H, 2-H), 4.28 (tdd, $J$ = 7.0, 3.5, 1.1 Hz, 1 H, 3-H), 3.77 (s, 3 H, $OCH_3$); $^{13}$C NMR (75 MHz, $CD_3OD$): δ = 160.7, 158.5 (C=O, C-Ph), 137.7 (C-Ph), 137.4 (C-4), 131.4 (CH-Ph), 131.1 (C-Ph), 129.5 (CH-Ph), 129.2 (CH-Ph), 129.1 (CH-Ph), 118.0 (C-5), 114.4 (CH-Ph), 74.8, 74.3 (C-2, C-3), 68.6 (O$CH_2$Ph), 64.4 (C-1), 55.7 ($OCH_3$); IR (ATR): $\nu_{max}$ = 3369, 2935, 1693, 1513, 1247, 1178, 1111, 1086, 1028, 744, 697, 563 cm$^{-1}$; HRMS (ESI): *m/z* calcd for $C_{20}H_{24}NO_6$: 374.1599 [M + H]$^+$; found: 374.1604.

**(±)-Benzyl [2,3-dihydroxy-1-(4-methoxyphenyl)pent-4-en-1-yl]carbamate (11)**

In a manner similar to [3], to a well-stirred solution of (hydroxyamino)diol *anti*,*syn*-**4** (700 mg, 1.87 mmol) in acetic acid (13 mL) was added zinc dust (4.90 g, 75.0 mmol), and the reaction mixture was stirred at 40 °C. The reaction progress was monitored by TLC [$CH_2Cl_2$/acetone/$NH_3$ 5:1:0.3 (aq., 26%)]. After stirring for 24 h, the reaction mixture was cooled to rt, diluted with 0.1 M NaOH solution (60 mL) and $CH_2Cl_2$ (40 mL), and the resulting slurry was vigorously stirred for 10 min. Insoluble solids were removed by filtration through a pad of Celite and sequentially washed with 0.1 M NaOH solution (15 mL) and $CH_2Cl_2$ (15 mL). After phase separation, the aqueous layer was extracted with $CH_2Cl_2$ (2 × 50 mL). The combined organic layers were dried over $MgSO_4$ and concentrated under reduced pressure. The product was isolated by FCC ($CH_2Cl_2$/acetone 9:1) to give *N*-Cbz-protected amino diol **11** (570 mg, 1.59 mmol, 85%) as a white solid. mp 93–94 °C; $R_f$ = 0.18 ($CH_2Cl_2$/acetone, 9:1); $^1$H NMR (300 MHz, $CD_3OD$): δ = 7.37–7.19 (m, 7 H, H-Ph), 6.90–6.85 (m, 2 H, H-Ph), 5.94 (ddd, $J$ = 17.2, 10.5, 6.3 Hz, 1 H, 4-H), 5.21 (ddd, $J$ = 17.2, 1.8, 1.4 Hz, 1 H, 5-$H_a$), 5.14 (ddd, $J$ = 10.5, 1.8, 1.3 Hz, 1 H, 5-$H_b$), 5.08 (d, $J$ = 12.5 Hz, 1 H, OC$H_2$Ph), 5.01 (d, $J$ = 12.5 H, 1 H, OC$H_2$Ph), 4.76 (d, $J$ = 6.7 Hz, 1 H, 1-H), 4.06–3.96 (m, 1 H, 3-H), 3.77 (s, 3 H, $OCH_3$), 3.66 (dd, $J$ = 6.6, 3.4 Hz, 1 H, 2-H); $^{13}$C NMR (75 MHz, $CD_3OD$): δ = 160.4, 158.2 (C=O, C-Ph), 139.8 (C-4), 138.3 (C-

Ph), 133.6 (C-Ph), 129.9 (CH-Ph), 129.4 (CH-Ph), 129.0 (CH-Ph), 128.9 (CH-Ph), 116.4 (C-5), 114.6 (CH-Ph), 76.7, 73.4 (C-2, C-3), 67.6 (O*C*$H_2$Ph), 58.5 (C-1), 55.7 ($OCH_3$); IR (ATR): $\nu_{max}$ = 3356, 2957, 1683, 1517, 1249, 1138, 1013, 836, 756, 700, 587 $cm^{-1}$; HRMS (ESI): *m/z* calcd for $C_{20}H_{23}NNaO_5$: 380.1469 $[M + Na]^+$; found: 380.1469.

**(±)-Benzyl [2,3-dihydroxy-1-(4-methoxyphenyl)-3-(oxiran-2-yl)propyl]carbamate (5)**

To a stirred solution of amino diol **11** (120 mg, 0.34 mmol) in EtOAc (0.8 mL) at rt was added pyridine (1.6 µL, 0.02 mmol), phosphotungstic acid hydrate (10 mg, 3.4 µmol) and hydrogen peroxide solution (86 µL, 1.0 mmol, 35 wt % in $H_2O$). The reaction mixture was stirred for 48 h. After this time, TLC showed that the reaction was complete ($CH_2Cl_2$/acetone 85:15). The reaction mixture was cooled to 0 °C, and sat. aq. $Na_2S_2O_3$ (20 mL) was added dropwise over 10 min. The mixture was diluted with EtOAc (15 mL), warmed to rt, and stirred for additional 5 min. The organic layer was separated, and the aqueous layer was extracted with EtOAc (2 × 15 mL). The combined organic layers were dried over $MgSO_4$ and the solvent was evaporated under reduced pressure. The residue was purified by FCC ($CH_2Cl_2$/acetone 9:1) to give a single *anti*,*syn*,*syn*-isomer of epoxide **5** (90 mg, 0.24 mmol, 70%) as a white solid. mp 112–113 °C; $R_f$ = 0.10 ($CH_2Cl_2$/acetone, 9:1); $^1H$ NMR (300 MHz, $CD_3OD$): δ = 7.37–7.19 (m, 7 H, H-Ph), 6.90–6.85 (m, 2 H, H-Ph), 5.07 (d, *J* = 12.4 Hz, 1 H, OC*H*$_2$Ph), 5.01 (d, *J* = 12.4 Hz, 1 H, OC*H*$_2$Ph), 4.80 (d, *J* = 7.7 Hz, 1 H, 1-H), 3.80 (dd, *J* = 7.8, 2.1 Hz, 1 H, 2-H), 3.77 (s, 3 H, $OCH_3$), 3.27 (dd, *J* = 6.4, 2.1 Hz, 1 H, 3-H), 3.13 (ddd, *J* = 6.4, 4.2, 2.7 Hz, 1 H, 4-H), 2.72 (dd, *J* = 4.9, 4.2 Hz, 1 H, 5-$H_a$), 2.49 (dd, *J* = 4.9, 2.7 Hz, 1 H, 5-$H_b$); $^{13}C$ NMR (75 MHz, $CD_3OD$): δ = 160.4, 158.3 (C=O, C-Ph), 138.2 (C-Ph), 133.7 (C-Ph), 129.7 (CH-Ph), 129.4 (CH-Ph), 129.0 (CH-Ph), 128.9 (CH-Ph), 114.7 (CH-Ph), 75.1, 73.4 (C-2, C-3), 67.6 (O*C*$H_2$Ph), 58.5 (C-1), 55.7, 55.2 ($OCH_3$, C-4), 45.0 (C-5); IR (ATR): $\nu_{max}$ = 3361, 2935, 1690, 1514, 1247, 1030, 833, 738, 698, 585 $cm^{-1}$.

**(±)-Benzyl 3,4-dihydroxy-2-(hydroxymethyl)-5-(4-methoxyphenyl)pyrrolidine-1-carboxylate (12)**

To a stirred solution of epoxide **5** (340 mg, 0.91 mmol) in $CH_2Cl_2$ (9 mL) at 0 °C was added $BF_3{\cdot}OEt_2$ (0.14 mL, 1.13 mmol) and the reaction mixture was stirred for 15 min. When TLC showed that the reaction was complete [$CH_2Cl_2$/MeOH/$NH_3$ 19:1:0.1 (aq., 26%)], sat. aq. $NH_4Cl$ (50 mL) and $CH_2Cl_2$ (50 mL) were added, and the mixture was vigorously stirred at rt for additional 5 min. Afterwards, the organic layer was separated, and the aqueous layer was extracted with $CH_2Cl_2$ (2 × 50 mL). The combined organic layers were dried over $MgSO_4$ and the solvent was evaporated under reduced pressure. The residue was purified by FCC ($CH_2Cl_2$/MeOH 95:5) to give *N*-Cbz-protected pyrrolidine **12** (235 mg, 0.63 mmol, 69%) as a white solid. mp 139–140 °C; $R_f$ = 0.08 ($CH_2Cl_2$/MeOH, 95:5); $^1H$

and $^{13}$C NMR spectra obtained at 25 °C show the presence of two *N*-Cbz rotamers in a ~2:1 ratio: $^{1}$H NMR (600 MHz, DMSO-$d_6$, 25 °C, major rotamer): δ = 7.19–7.11 (m, 5 H, H-Ph), 6.85–6.79 (m, 2 H, H-Ph), 6.72–6.69 (m, 2 H, H-Ph), 5.55 (d, *J* = 4.9 Hz, 1 H, OH), 5.09 (d, *J* = 3.2 Hz, 1 H, OH), 5.06–5.00 (m, 1 H, OH), 4.90 (d, *J* = 13.1 Hz, 1 H, OC$H_2$Ph), 4.77 (d, *J* = 13.1 Hz, 1 H, OC$H_2$Ph), 4.61–4.59 (m, 1 H, 5-H), 4.06–4.03 (m, 1 H, 3-H), 3.91–3.87 (m, 1 H, 2-H), 3.82–3.79 (m, 1 H, 4-H), 3.76–3.72 (m, 5 H, C$H_2$OH, $OCH_3$); $^{13}$C NMR (150 MHz, DMSO-$d_6$, 25 °C, major rotamer): δ = 157.9 (C-Ph), 154.0 (C=O), 136.6 (C-Ph), 134.4 (C-Ph), 127.9 (CH-Ph), 127.5 (CH-Ph), 127.2 (CH-Ph), 126.7 (CH-Ph), 113.2 (CH-Ph), 83.2 (C-4), 77.0 (C-3), 70.4 (C-5), 68.7 (C-2), 65.5 (O*C*$H_2$Ph), 58.9 (*C*$H_2$OH), 55.1 ($OCH_3$); $^{1}$H NMR (600 MHz, DMSO-$d_6$, 25 °C, minor rotamer): δ = 7.39–7.27 (m, 5 H, H-Ph), 7.19–7.11 (m, 2 H, H-Ph), 6.85–6.79 (m, 2 H, H-Ph), 5.57 (d, *J* = 4.6 Hz, 1 H, OH), 5.06–5.00 (m, 4 H, OC$H_2$Ph, 2 × OH), 4.59–4.58 (m, 1 H, 5-H), 4.10–4.07 (m, 1 H, 3-H), 3.91–3.87 (m, 1 H, 2-H), 3.79–3.77 (m, 1 H, 4-H), 3.71 (s, 3 H, $OCH_3$), 3.69–3.65 (m, 2 H, C$H_2$OH); $^{13}$C NMR (150 MHz, DMSO-$d_6$, 25 °C, minor rotamer): δ = 157.7 (C-Ph), 153.6 (C=O), 137.0 (C-Ph), 133.5 (C-Ph), 128.4 (CH-Ph), 127.8 (CH-Ph), 127.6 (CH-Ph), 127.5 (CH-Ph), 113.0 (CH-Ph), 82.6 (C-4), 77.9 (C-3), 71.0 (C-5), 68.3 (C-2), 65.8 (O*C*$H_2$Ph), 60.0 (*C*$H_2$OH), 55.0 ($OCH_3$); IR (ATR): $\nu_{max}$ = 3326, 2903, 1673, 1427, 1351, 1200, 1035, 861, 744, 695, 586 $cm^{-1}$; HRMS (ESI): *m/z* calcd for $C_{20}H_{24}NO_6$: 374.1599 $[M + H]^+$; found: 374.1600; *m/z* calcd for $C_{20}H_{23}NNaO_6$: 396.1418 $[M + Na]^+$; found: 396.1418.

**(±)-2-(Hydroxymethyl)-5-(4-methoxyphenyl)pyrrolidine-3,4-diol (2)**

To a solution of pyrrolidine **12** (180 mg, 0.48 mmol) in anhydrous MeOH (28 mL) was added $Pd(OH)_2$/C (90 mg, 5 wt %), and the reaction mixture was vigorously stirred under $H_2$ atmosphere (1 atm) at rt for 2 h. After this time, TLC showed that the reaction was complete [$CH_2Cl_2$/MeOH/$NH_3$ 9:1:0.1 (aq., 26%)]. The catalyst was removed by filtration through a pad of Celite and washed with MeOH (10 mL). The filtrate was concentrated under reduced pressure. The residue was crystallized from EtOAc to give unprotected pyrrolidine **2** (81 mg, 0.34 mmol, 71%) as a white needle-like crystals with spectroscopic data in good agreement with those reported in the literature [4]. mp 143–144 °C; $R_f$ = 0.06 ($CH_2Cl_2$/MeOH, 9:1); $^{1}$H NMR (300 MHz, $D_2O$): δ = 7.44–7.39 (m, 2 H, H-Ph), 7.07–7.02 (m, 2 H, H-Ph), 4.12 (dd, *J* = 9.1,7.5 Hz, 1 H, 4-H), 3.97 (t, *J* = 7.4 Hz, 1 H, 3-H), 3.93 (d, *J* = 9.1 Hz, 1 H, 5-H), 3.86 (s, 3 H, $OCH_3$), 3.77 (dd, *J* = 11.6, 4.7 Hz, 1 H, C$H_2$OH), 3.71 ( dd, *J* = 11.6, 6.4 Hz, 1 H, C$H_2$OH), 3.26 (td, *J* = 6.7, 4.7 Hz, 1 H, 2-H); $^{1}$H NMR (300 MHz, $CD_3OD$): δ = 7.37–7.32 (m, 2 H, H-Ph), 6.93–6.88 (m, 2 H, H-Ph), 3.97–3.85 (m, 3 H, 3-H, 4-H, 5-H), 3.78 (s, 3 H, $OCH_3$), 3.72 (dd, *J* = 11.1, 4.3 Hz, 1 H, C$H_2$OH), 3.64 ( dd, *J* = 11.1, 6.2 Hz, 1 H, C$H_2$OH), 3.19 (td, *J* = 6.2, 4.3 Hz, 1 H, 2-H); $^{13}$C NMR (75 MHz, $CD_3OD$): δ = 160.7 (C-Ph), 134.8 (C-Ph), 129.5 (CH-Ph), 114.9 (CH-Ph), 85.0, 79.5 (C-3, C-4), 66.1 (C-5), 64.5 (C-2), 63.8 (*C*$H_2$OH), 55.7 ($OCH_3$); IR (ATR): $\nu_{max}$ = 3210, 2912, 2398, 1513, 1250, 1183, 1028, 967, 825, 753, 669, 560 $cm^{-1}$.

**(±)-2-(Hydroxymethyl)-5-(4-methoxyphenyl)-1-methylpyrrolidine-3,4-diol, (±)-codonopsinol B (1)**

To a solution of pyrrolidine **12** (180 mg, 0.48 mmol) in anhydrous MeOH (28 mL) was added $Pd(OH)_2/C$ (90 mg, 5 wt %) and the reaction mixture was vigorously stirred under $H_2$ atmosphere (1 atm) at rt for 2 h. When TLC showed that the protecting group was removed [$CH_2Cl_2/MeOH/NH_3$ 9:1:0.1 (aq., 26%)], formaldehyde solution (0.54 mL, 7.2 mmol, 37wt % in $H_2O$) was added, and the reaction mixture was stirred at rt under $H_2$ atmosphere (1 atm) for additional 16 h. After this time, the catalyst was removed by filtration through a pad of Celite and washed with MeOH (10 mL). The filtrate was concentrated under reduced pressure. The residue was purified by FCC ($CH_2Cl_2/MeOH$ 9:1) to give (±)-codonopsinol B (**1**, 70 mg, 0.28 mmol, 58% over two steps) as a colourless sticky oil with spectroscopic data in good agreement with those reported in the literature [4,5]. $R_f$ = 0.10 ($CH_2Cl_2/MeOH$, 9:1); $^1H$ NMR (300 MHz, $CD_3OD$): δ = 7.33–7.28 (m, 2 H, H-Ph), 6.94–6.89 (m, 2 H, H-Ph), 4.04 (pseudo t, $J$ = 4.7, 4.6 Hz, 1 H, 3-H), 3.99 (dd, $J$ = 6.6, 4.8 Hz, 1 H, 4-H), 3.88 (dd, $J$ = 11.6, 4.2 Hz, 1 H, $CH_2OH$), 3.83 (dd, $J$ = 11.6, 4.2 Hz, 1 H, $CH_2OH$), 3.79 (s, 3 H, $OCH_3$), 3.74 (d, $J$ = 6.6 Hz, 1 H, 5-H), 3.12 (pseudo q, $J$ = 4.2, 4.3 Hz, 1 H, 2-H), 2.22 (s, 3 H, $NCH_3$); $^{13}C$ NMR (75 MHz, $CD_3OD$): δ = 161.2 (C-Ph), 131.1 (CH-Ph), 131.0 (C-Ph), 115.0 (CH-Ph), 84.1 (C-4), 79.0 (C-3), 75.2 (C-5), 71.5 (C-2), 60.1 ($CH_2OH$), 55.7 ($OCH_3$), 35.4 ($NCH_3$).

## Crystallography

Data collection and cell refinement for **12** were carried out with a Stoe StadiVari diffractometer equipped with a Pilatus3R 300K HPD detector, and using CuKα radiation (λ = 1.54186 Å, microfocussed source Xenocs Genix3D Cu HF) at 100 K. The structure was solved using *Sir14* [6] and refined by a full-matrix least-squares procedure of independent atom model (AIM) with the *SHELXL* (ver. 2018/3) [7]. The 4-methoxyphenyl group is disordered in two positions with occupancy factors 0.64 and 0.36 for main and minor parts (Figures S1 and S2). The disordered 4-methoxyphenyl group has been modelled using SAME, SADI, RIGU, and EADP instructions. The Hirshfeld atom refinement (HAR) was carried out using IAM model as a starting point. The wave function was calculated using *ORCA* software [8,9] with basis set def2-TZVP and method PBE0. The least-squares refinements of HAR model were then carried out with *olex2.refine* [10], while keeping the same constrains and restrains as for the *SHELXL* refinement. The *NoSpherA2* implementation [11] of HAR makes used for tailor-made aspherical atomic factors calculated on-the-fly from a Hirshfeld-partitioned electron density. For the HAR approach, all H atoms were refined isotropically and independently. All calculations and structure drawings were done in the *OLEX2* package [12].

**Crystal data for 12**

$C_{20}H_{23}NO_6$ ($M$ = 373.39 g/mol): monoclinic, space group $C2/c$ (no. 15), $a$ = 35.2705(13) Å, $b$ = 5.8905(2) Å, $c$ = 17.6025(7) Å, $\beta$ = 94.974(3)°, $V$ = 3643.3(2) Å$^3$, $Z$ = 8, $T$ = 100.0 K, μ(CuKα) = 0.837 mm$^{-1}$, $Dcalc$ = 1.361 g/cm$^3$, 51482 reflections measured (5.03° ≤ 2Θ ≤ 144.05°), 3538 unique ($R_{int}$ = 0.0785, $R_{sigma}$ = 0.0296) which were used in all calculations. IAM: The final $R_1$ was 0.0391 (I > 2σ(I)) and $wR_2$ was 0.1017 (all data). CCDC no 2058692. HAR: The final $R_1$ was 0.0294 (I > 2σ(I)) and $wR_2$ was 0.0693 (all data). CCDC no 2099851.

## Cell culture and cultivation conditions

The antiproliferative effect of the tested compounds was evaluated on different cell lines. Cells were cultivated in humidified atmosphere with 5% $CO_2$ concentration at 37 °C.
The human glioblastoma astrocytoma cancer cell line U87-MG (purchased from ATCC, USA) was cultivated in Dulbecco′s Modified Eagle′s Medium (DMEM) High Glucose containing L-glutamine (2 mM), sodium pyruvate (1 mM), 1% non-essential amino acids (NAA) and 10% fetal bovine serum (FBS).
The HK-2 (purchased from ATCC, USA) is an immortalized proximal tubular cell line. The cells were maintained in DMEM High Glucose supplemented with 2 mM L-glutamine and 10% FBS.
The hepatocellular carcinoma cell line HepG2 (purchased from ECACC, UK) was cultivated in DMEM High Glucose containing L-glutamine (2 mM), 10% FBS, and 1% NAA.
The human placental choriocarcinoma cells JEG-3 (purchased from ECACC, UK) were maintained in Minimum Essential Medium Eagle (MEM) supplemented with Earle's Salts, sodium bicarbonate, 1% NAA, sodium pyruvate (1 mM), and 10% FBS.
The acute myeloid leukemia MOLM-13 cell line (DSMZ, GE) was cultivated in RPMI-1640 medium supplemented with 12% fetal bovine serum (both purchased from Gibco, Miami, OK, USA), 100,000 units/mL penicillin and 50 mg/L streptomycin.
All used supplements and mediums were purchased from Sigma-Aldrich, USA except for FBS purchased from Biosera, South America.

## Cell viability testing

For the U87-MG, HepG2, JEG-3 cancer cell lines, as well as immortalized proximal tubular cells HK2 the potential antiproliferative effect of tested substances was determined using the CellTiter 96® AQueous One Solution Cell Proliferation Assay (Promega, USA). The whole evaluation was proceeded according to the manufacturer’s instructions. The tested cells were seeded in a 96-well plate (TPP, Switzerland) at low density 4 × 10$^3$ cells per well and subsequently incubated for 24 h. The cells were exposed to the tested compounds at a concentration range (1–1000 µM), 1% DMSO

(non-toxic control, 100% viability) or 10% DMSO (toxic control, 100% mortality). The compounds were dissolved in DMSO (Sigma-Aldrich, USA), the volume of solvent was 1% (v/v) in the cultivation medium. The cells were incubated for 24, 48, or 72 h under standard culture conditions. The $IC_{50}$ values were calculated using the GraphPad Prism 8.3.1 software.

In the case of AML cell line MOLM-13, the cells were treated with compounds at a concentration range (0.5–500 µM). The DMSO concentration to dissolve the tested substances was 1% (non-toxic concentration). After the treatment, the cells were incubated for 48 h under standard culture conditions and the antiproliferative effect of the tested substances was also determined using CellTiter 96® AQueous One Solution Cell Proliferation Assay (Promega, USA). Numerical data are expressed as the mean ± SD of three independent measurements.

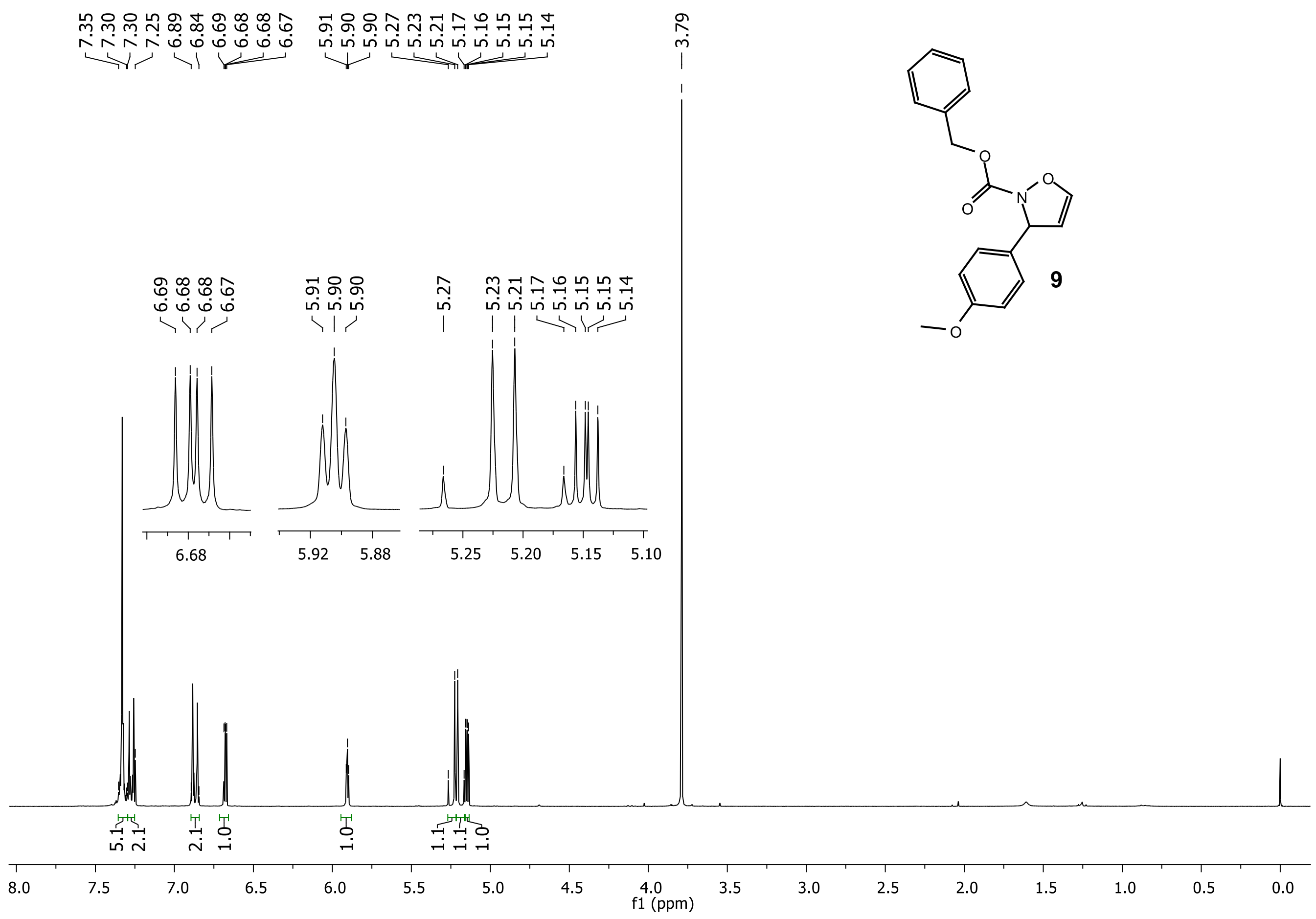
7.35 7.30 7.30 7.25 6.89 6.84 6.69 6.68 6.68 6.67 5.91 5.90 5.90 5.27 5.23 5.21 5.17 5.16 5.15 5.15 5.14
3.79
9
5.1 2.1 2.1 1.0 1.0 1.1 1.1 1.0
f1 (ppm)

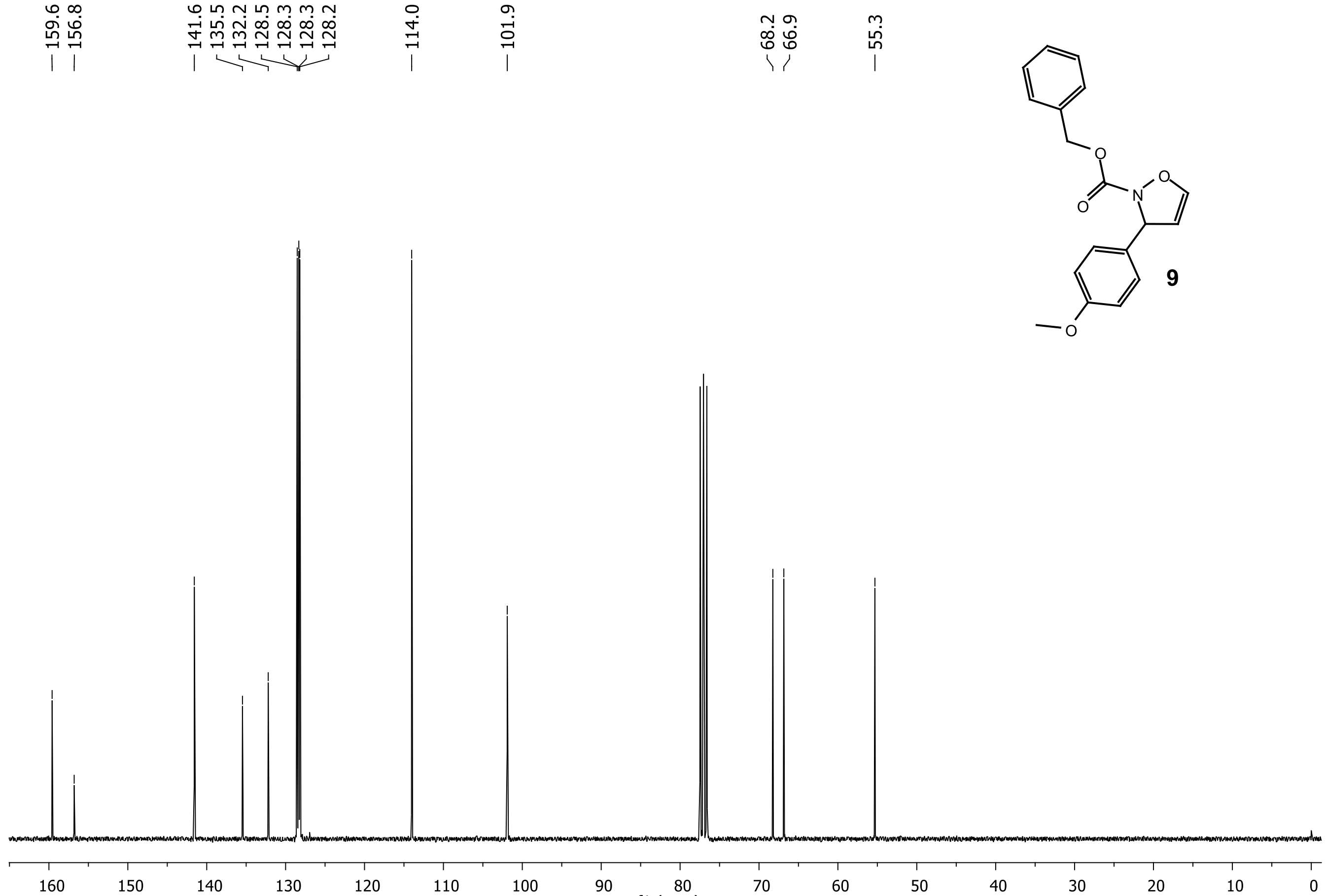
159.6 156.8 141.6 135.5 132.2 128.5 128.3 128.3 128.2 114.0 101.9 68.2 66.9 55.3
9
f1 (ppm)

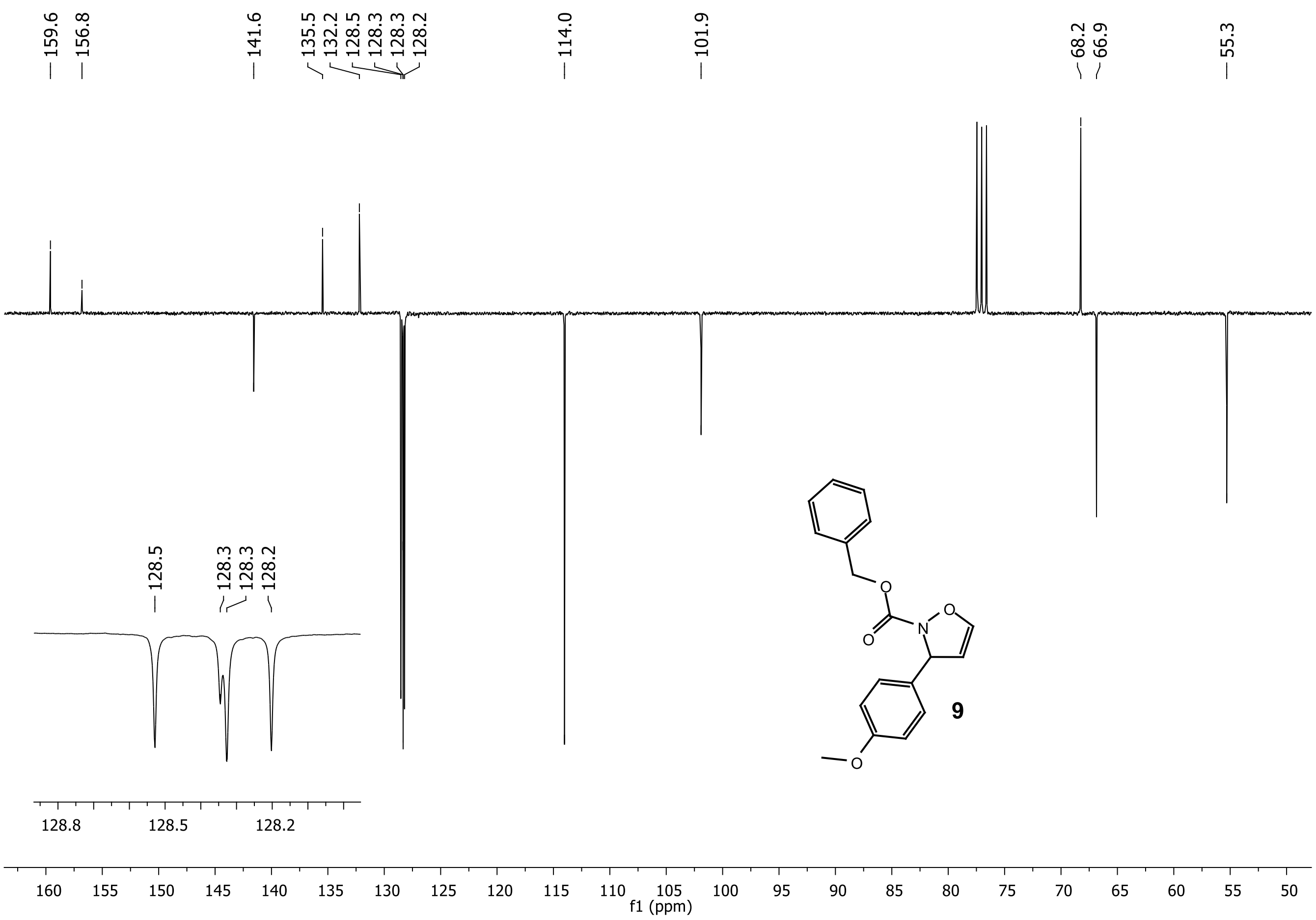
159.6
156.8
141.6
135.5
132.2
128.5
128.3
128.3
128.2
114.0
101.9
68.2
66.9
55.3
128.5
128.3
128.3
128.2
128.8
128.5
128.2
9
160 155 150 145 140 135 130 125 120 115 110 105 100 95 90 85 80 75 70 65 60 55 50
f1 (ppm)

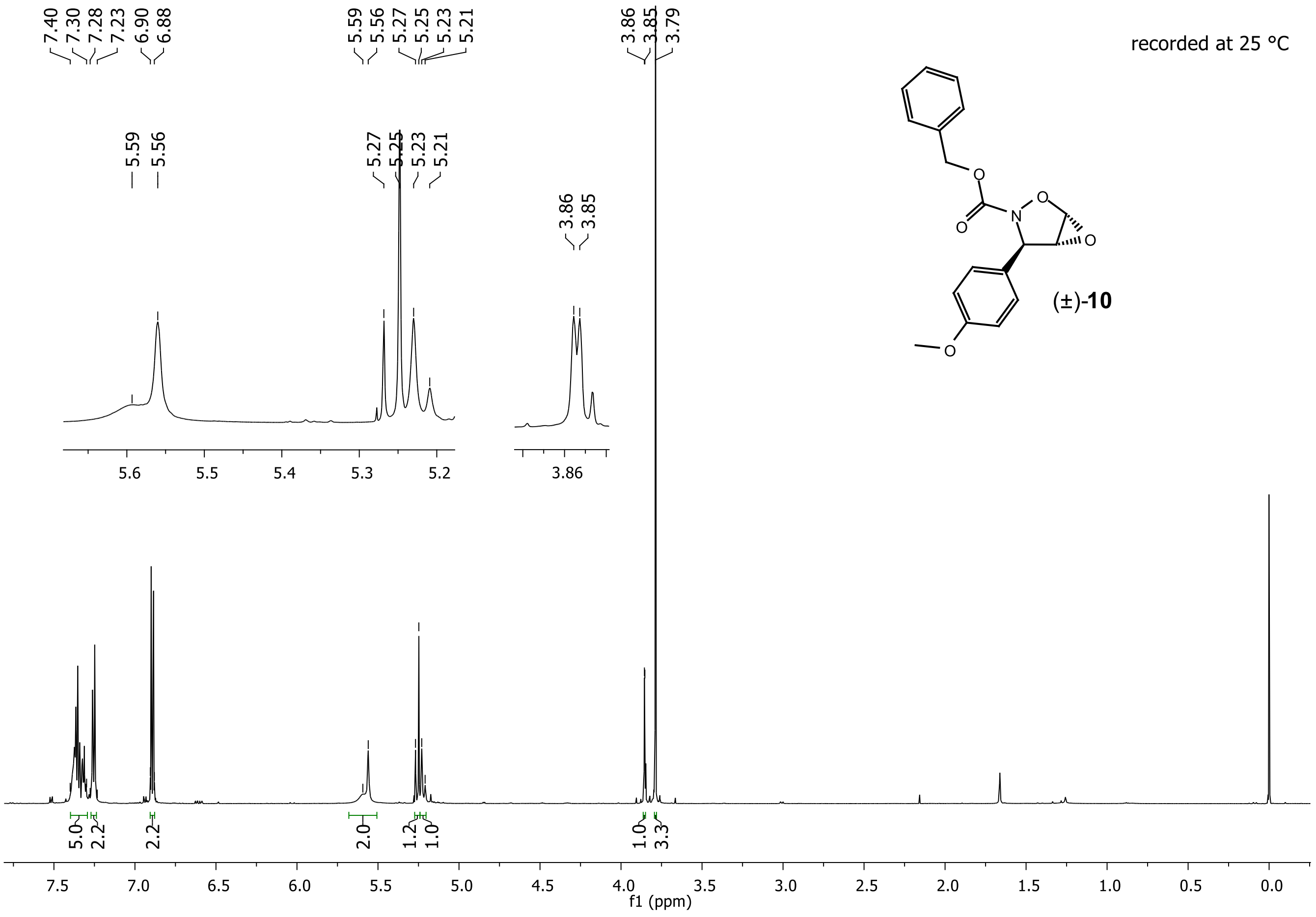
recorded at 25 °C
(±)-10
f1 (ppm)

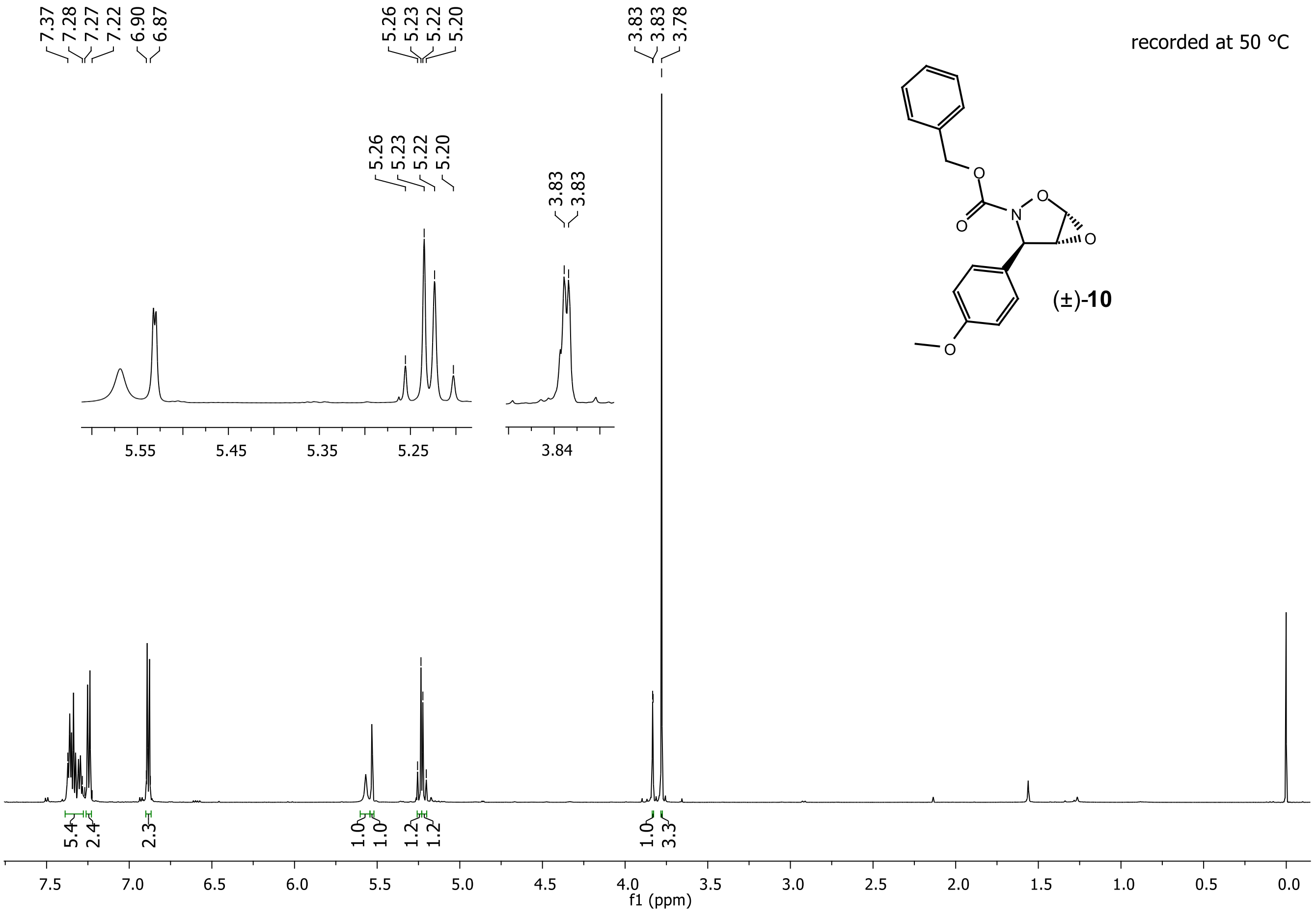
recorded at 50 °C
(±)-10
f1 (ppm)

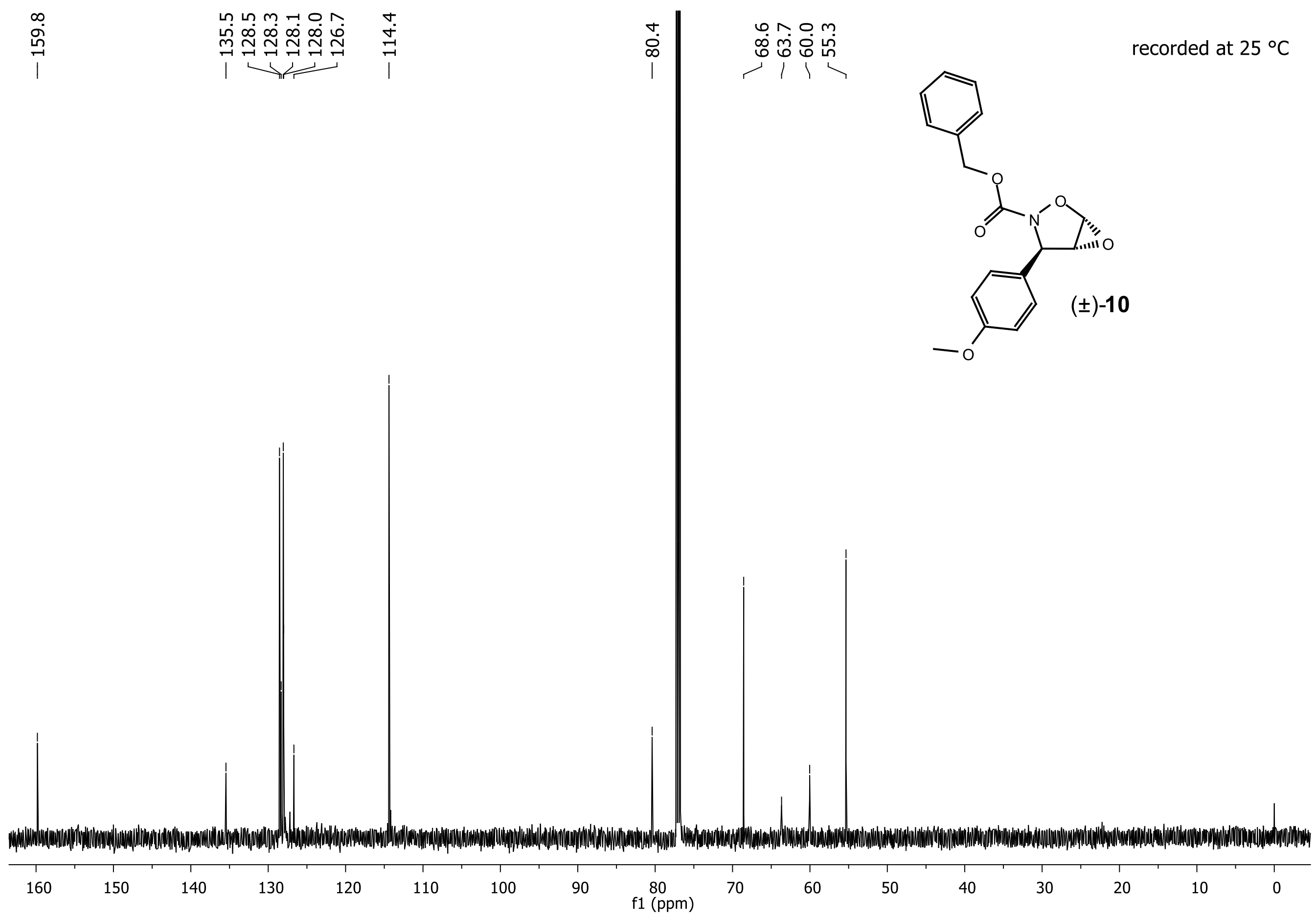
159.8
135.5
128.5
128.3
128.1
128.0
126.7
114.4
80.4
68.6
63.7
60.0
55.3
recorded at 25 °C
(±)-**10**
f1 (ppm)

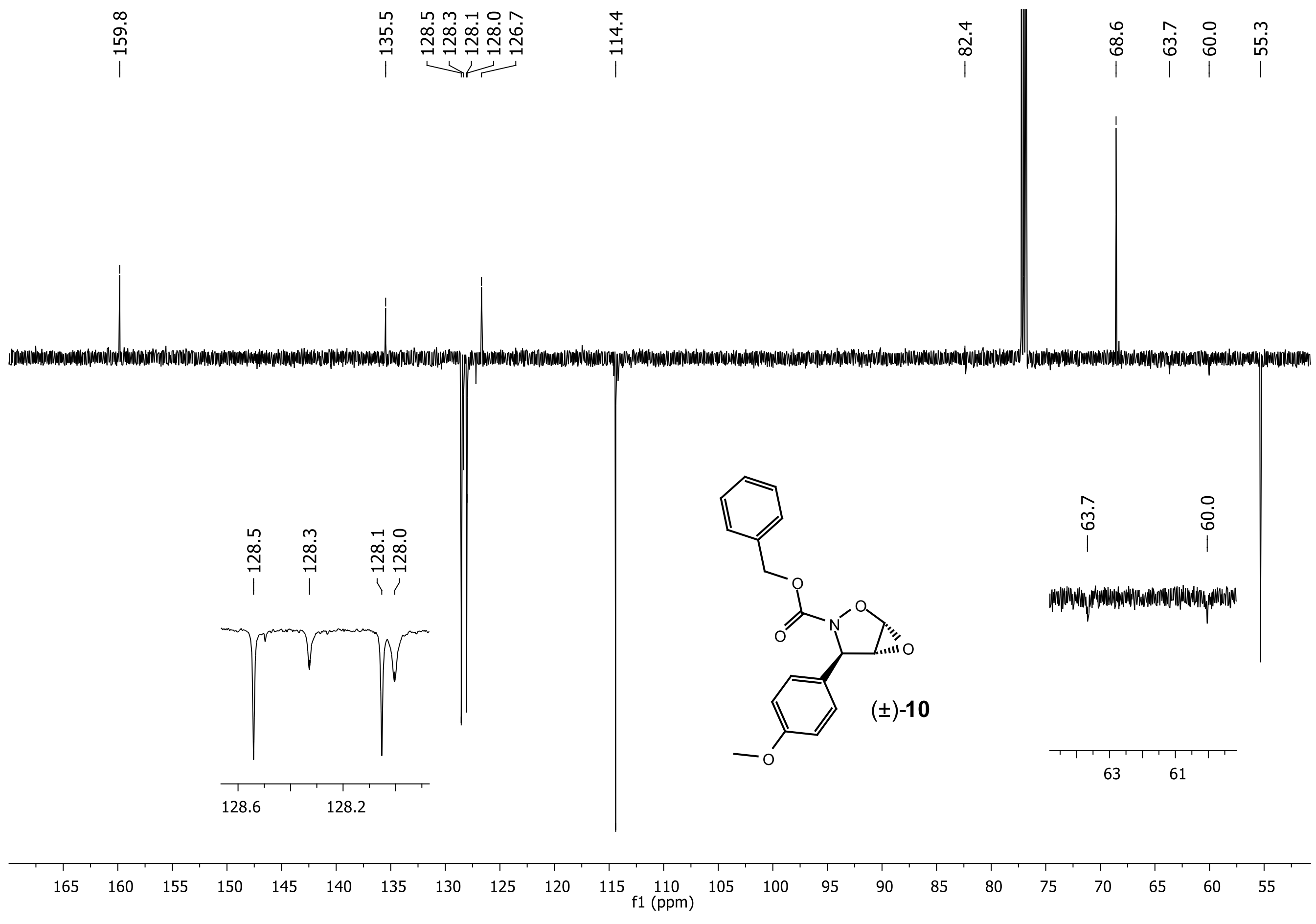
159.8
135.5
128.5
128.3
128.1
128.0
126.7
114.4
82.4
68.6
63.7
60.0
55.3
(±)-**10**
f1 (ppm)

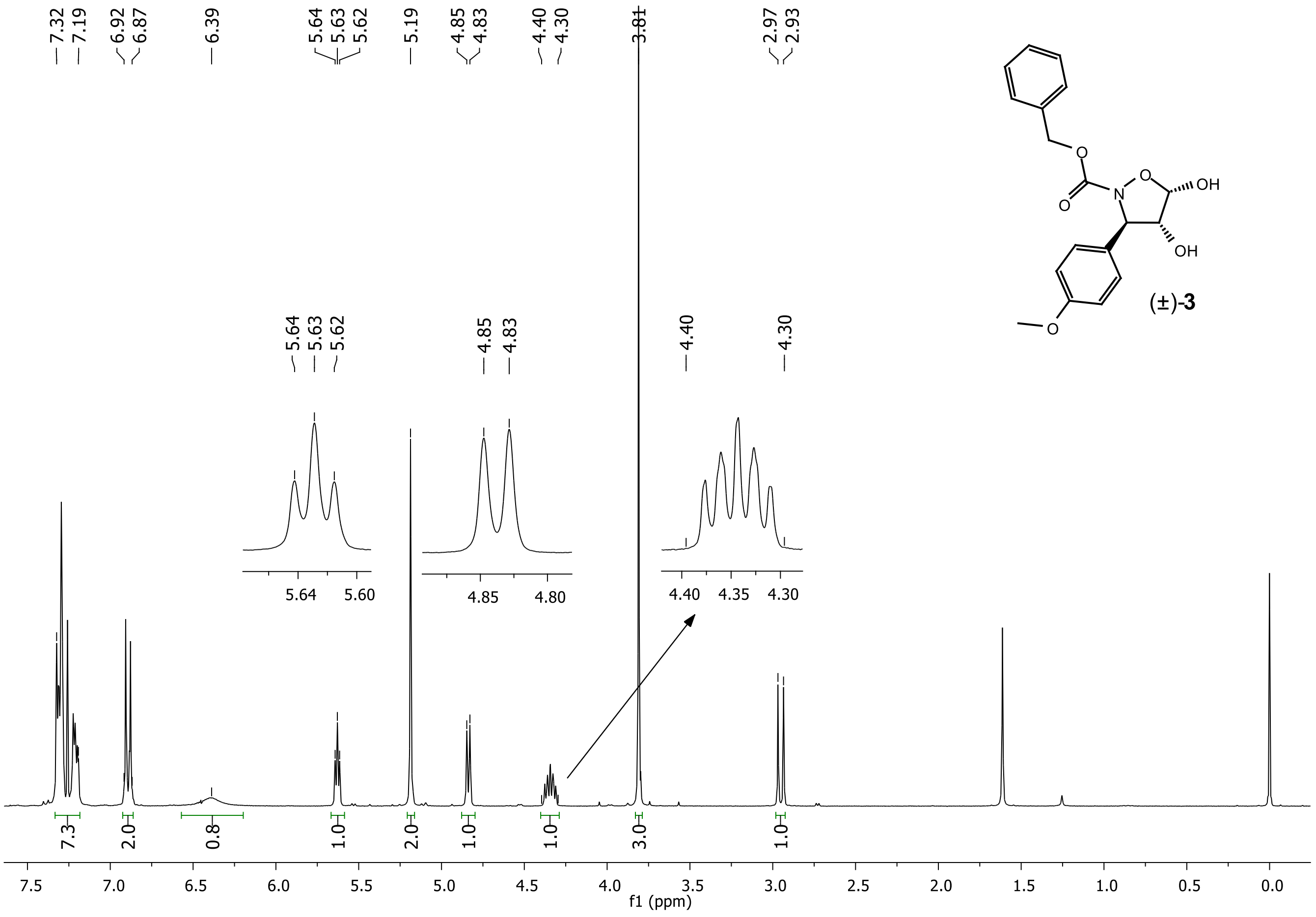
(±)-3
f1 (ppm)

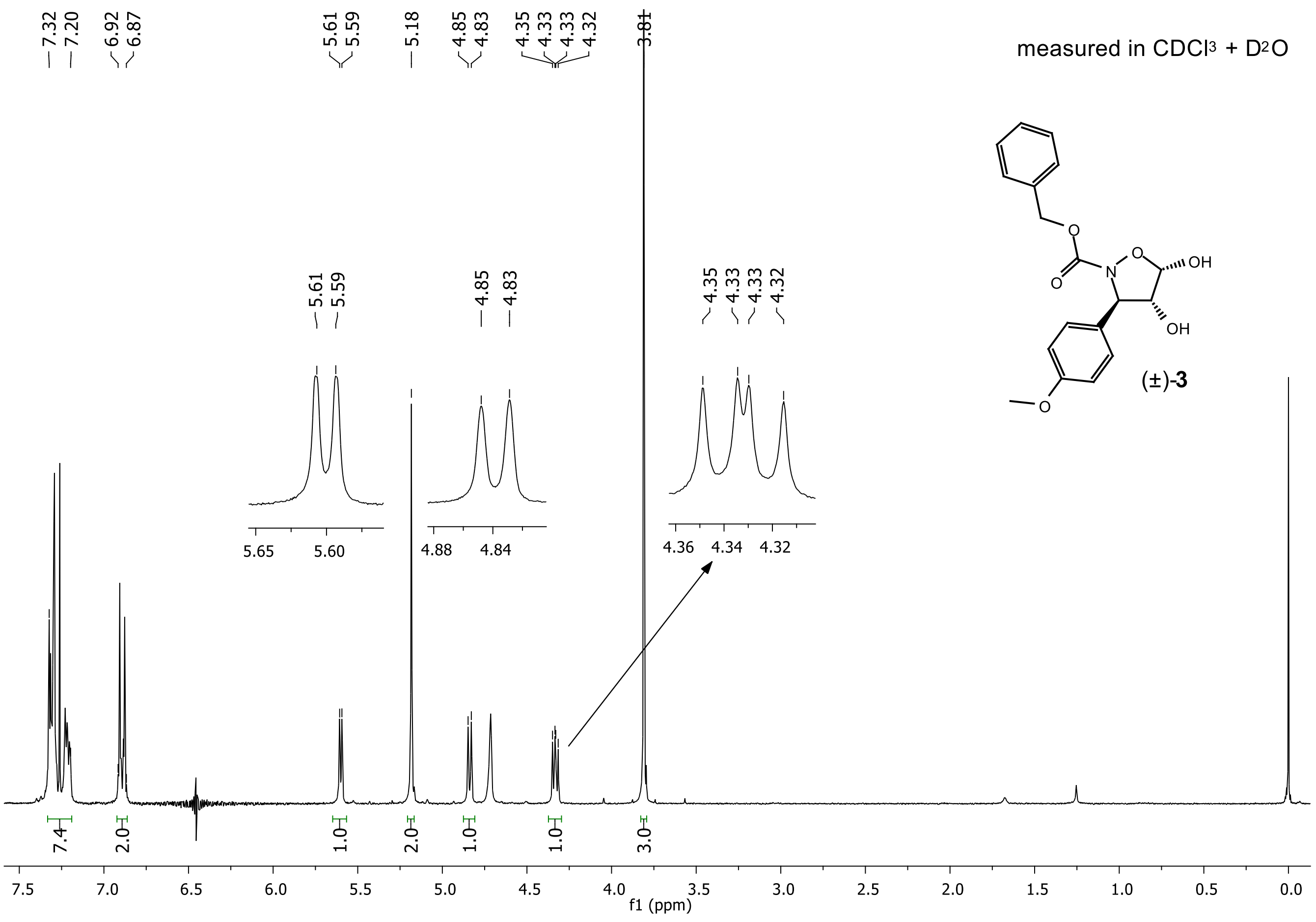
measured in CDCl3 + D2O
(±)-3
f1 (ppm)

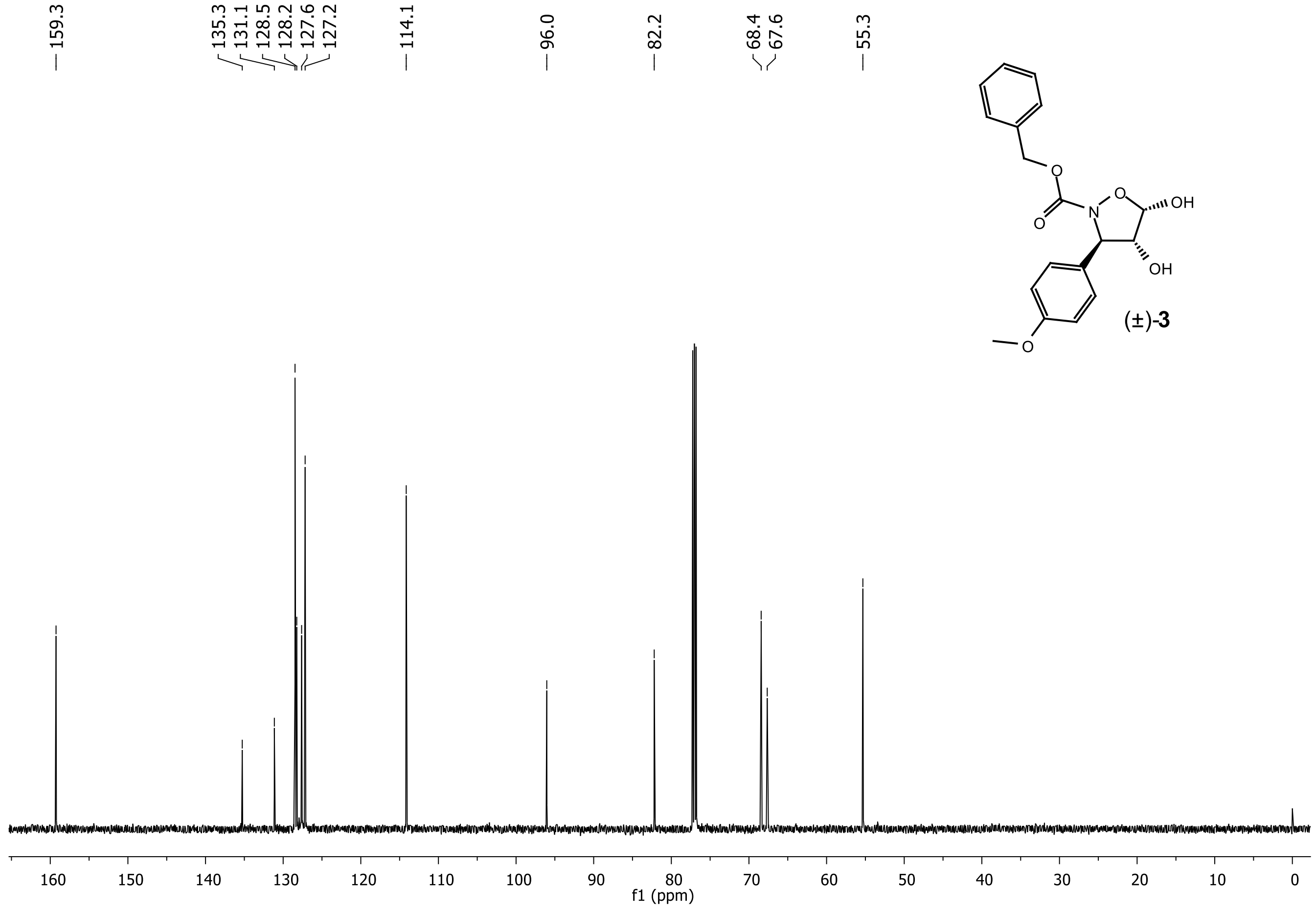
159.3
135.3
131.1
128.5
128.2
127.6
127.2
114.1
96.0
82.2
68.4
67.6
55.3
OH
OH
O
N
O
O
O
(±)-3
160 150 140 130 120 110 100 90 80 70 60 50 40 30 20 10 0
f1 (ppm)

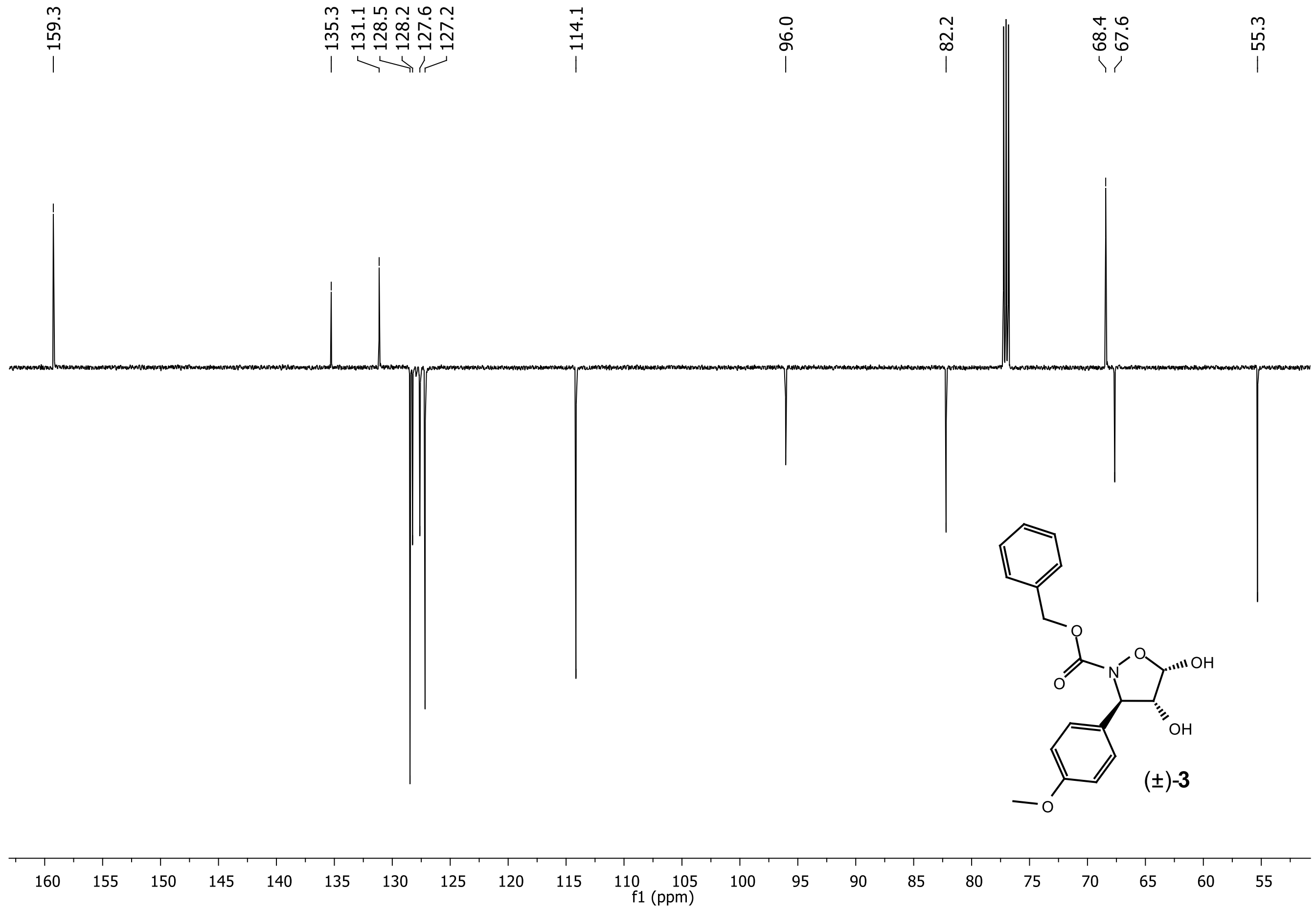
159.3
135.3
131.1
128.5
128.2
127.6
127.2
114.1
96.0
82.2
68.4
67.6
55.3
OH
OH
O
N
O
O
O
(±)-3
160 155 150 145 140 135 130 125 120 115 110 105 100 95 90 85 80 75 70 65 60 55
f1 (ppm)

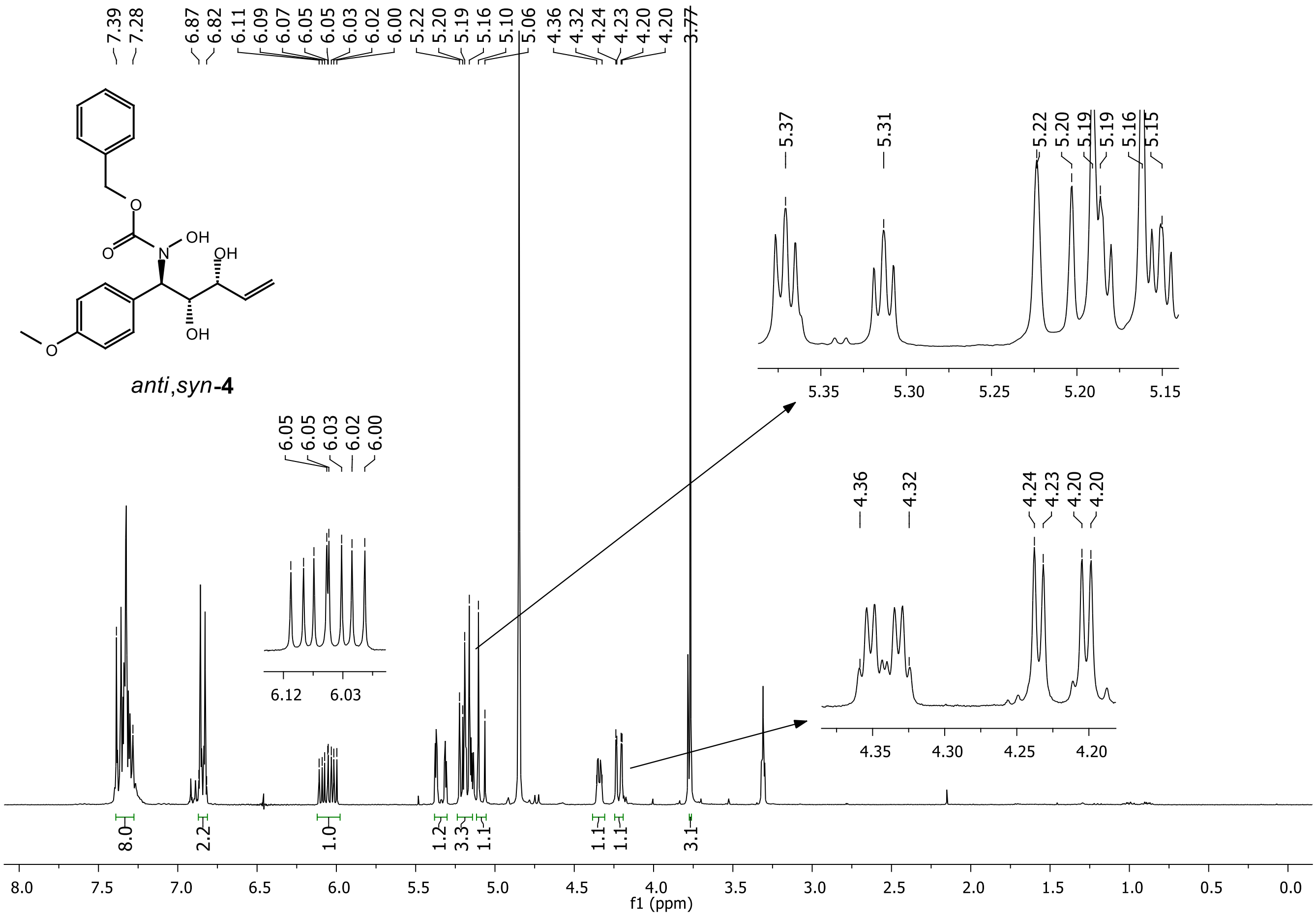

anti,syn-**4**

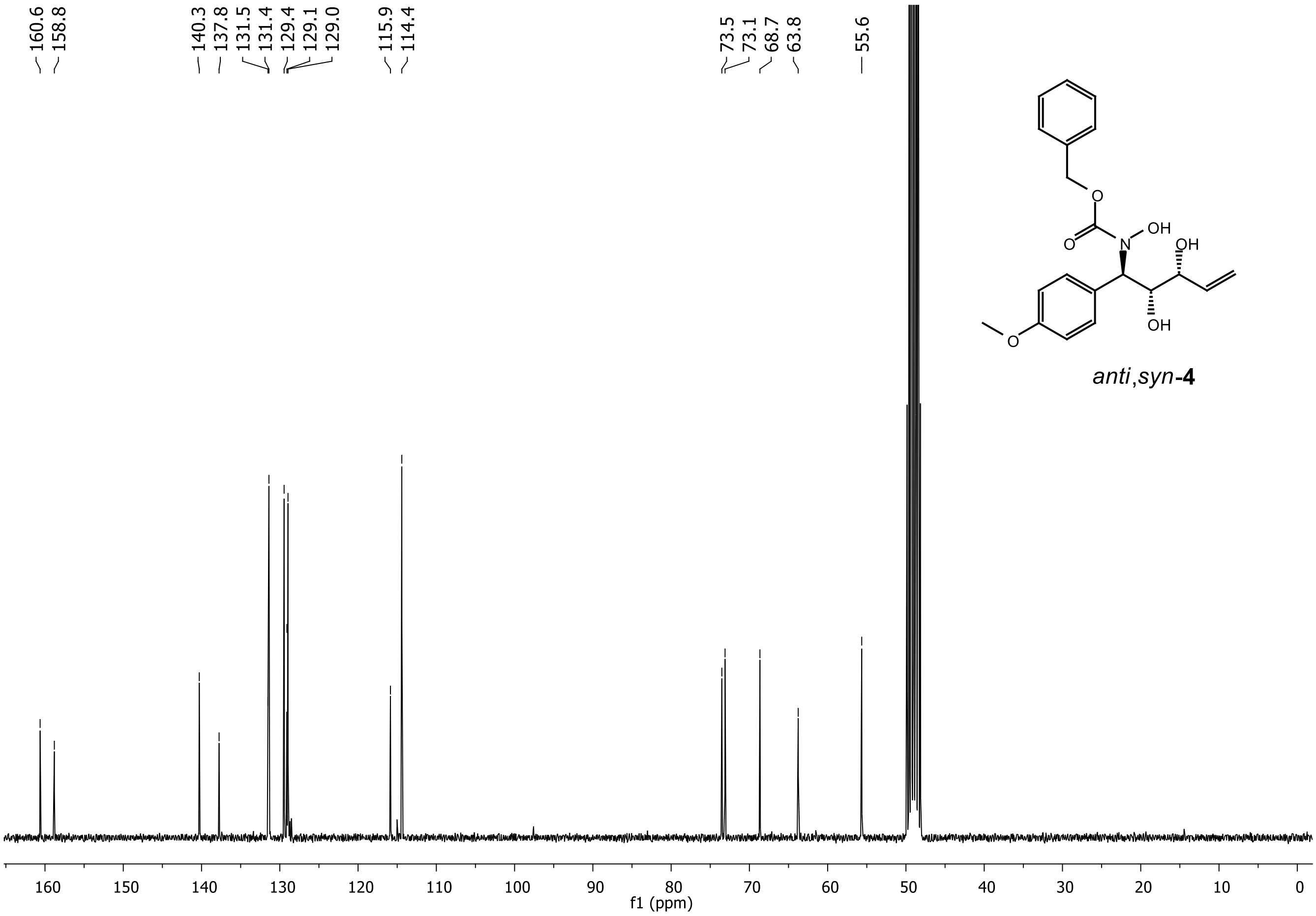

anti,syn-**4**

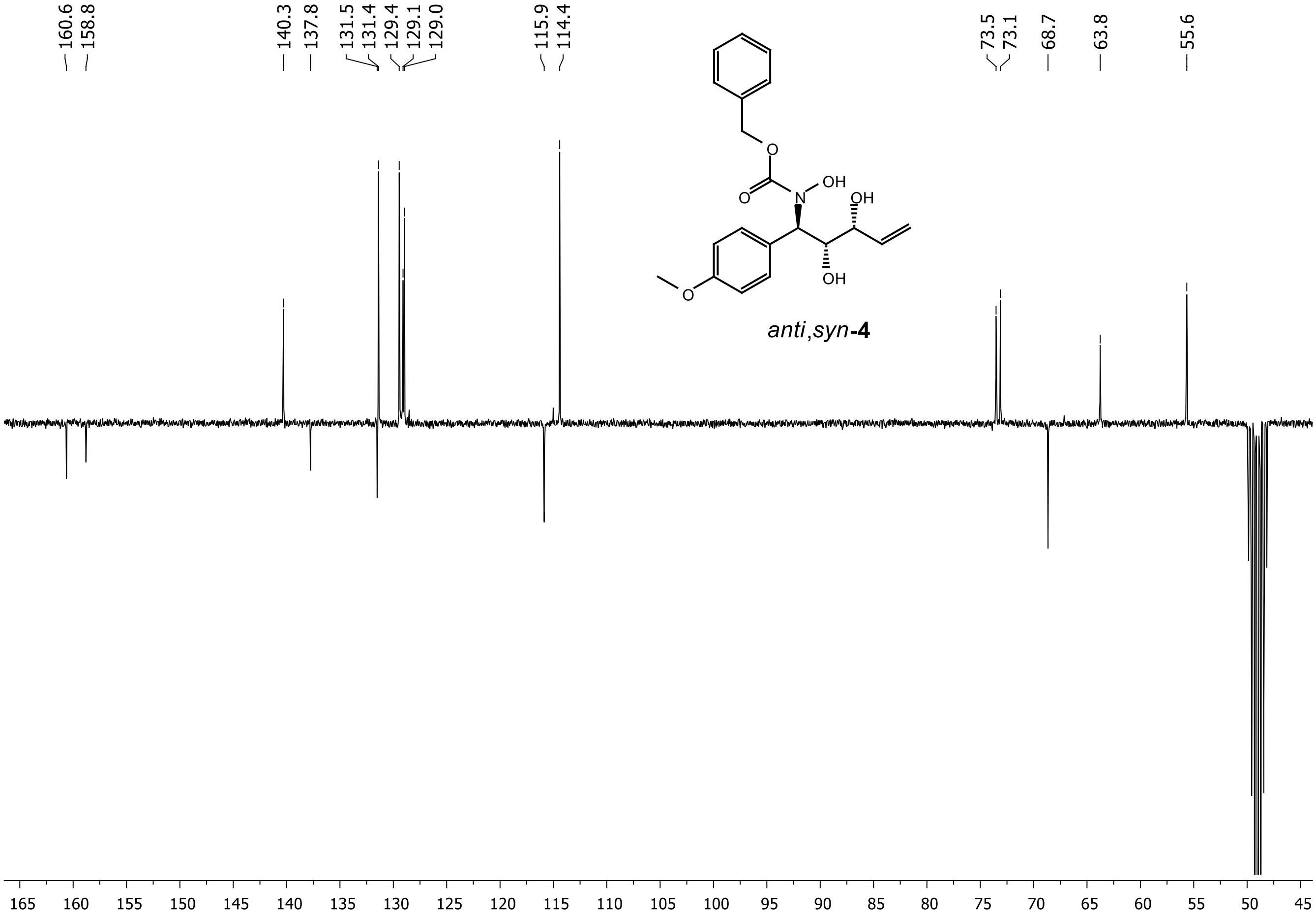
160.6
158.8
140.3
137.8
131.5
131.4
129.4
129.1
129.0
115.9
114.4
73.5
73.1
68.7
63.8
55.6
O
N
OH
OH
OH
165 160 155 150 145 140 135 130 125 120 115 110 105 100 95 90 85 80 75 70 65 60 55 50 45

*anti*,*syn*-**4**

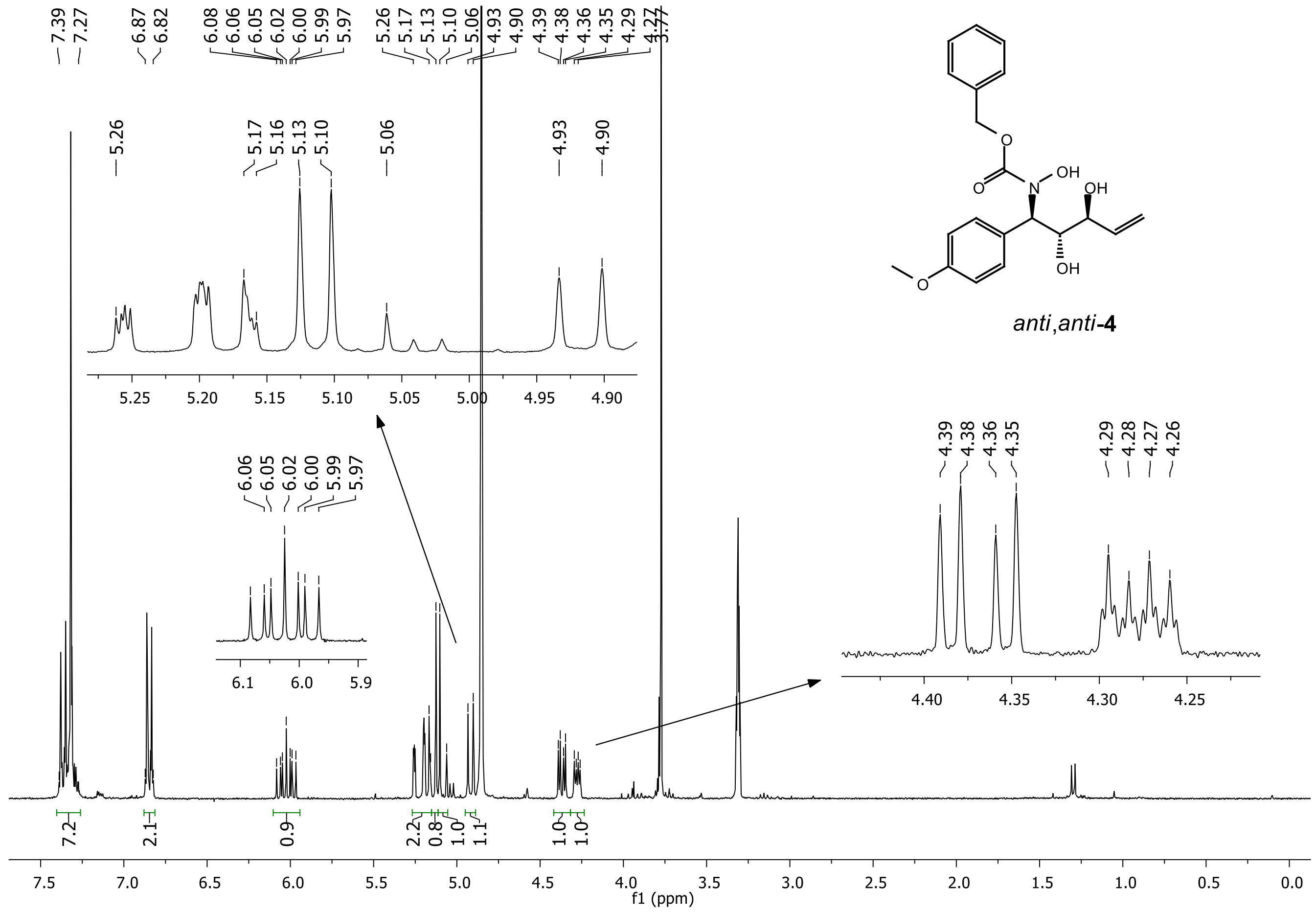
anti,anti-4
f1 (ppm)

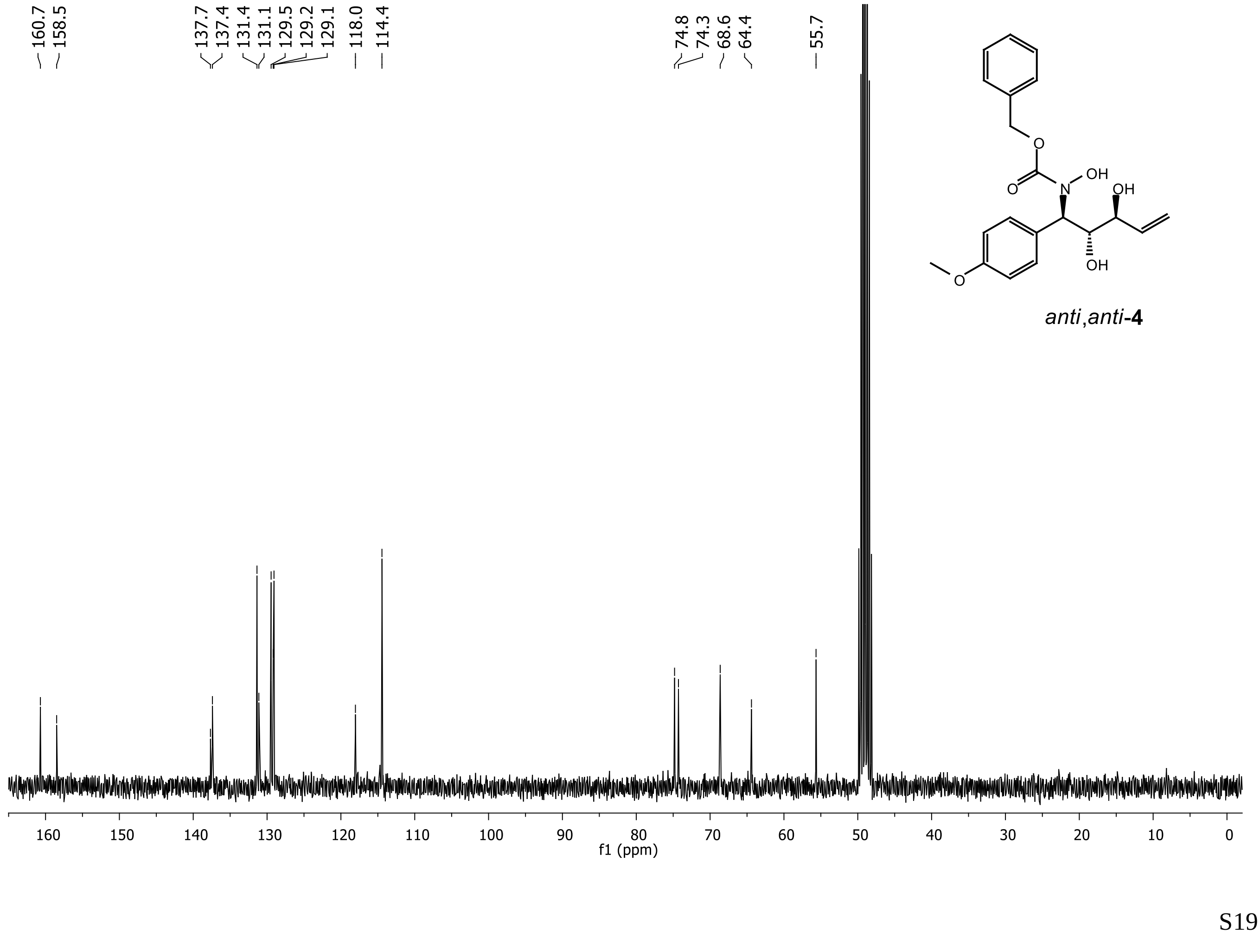
anti,anti-4
f1 (ppm)

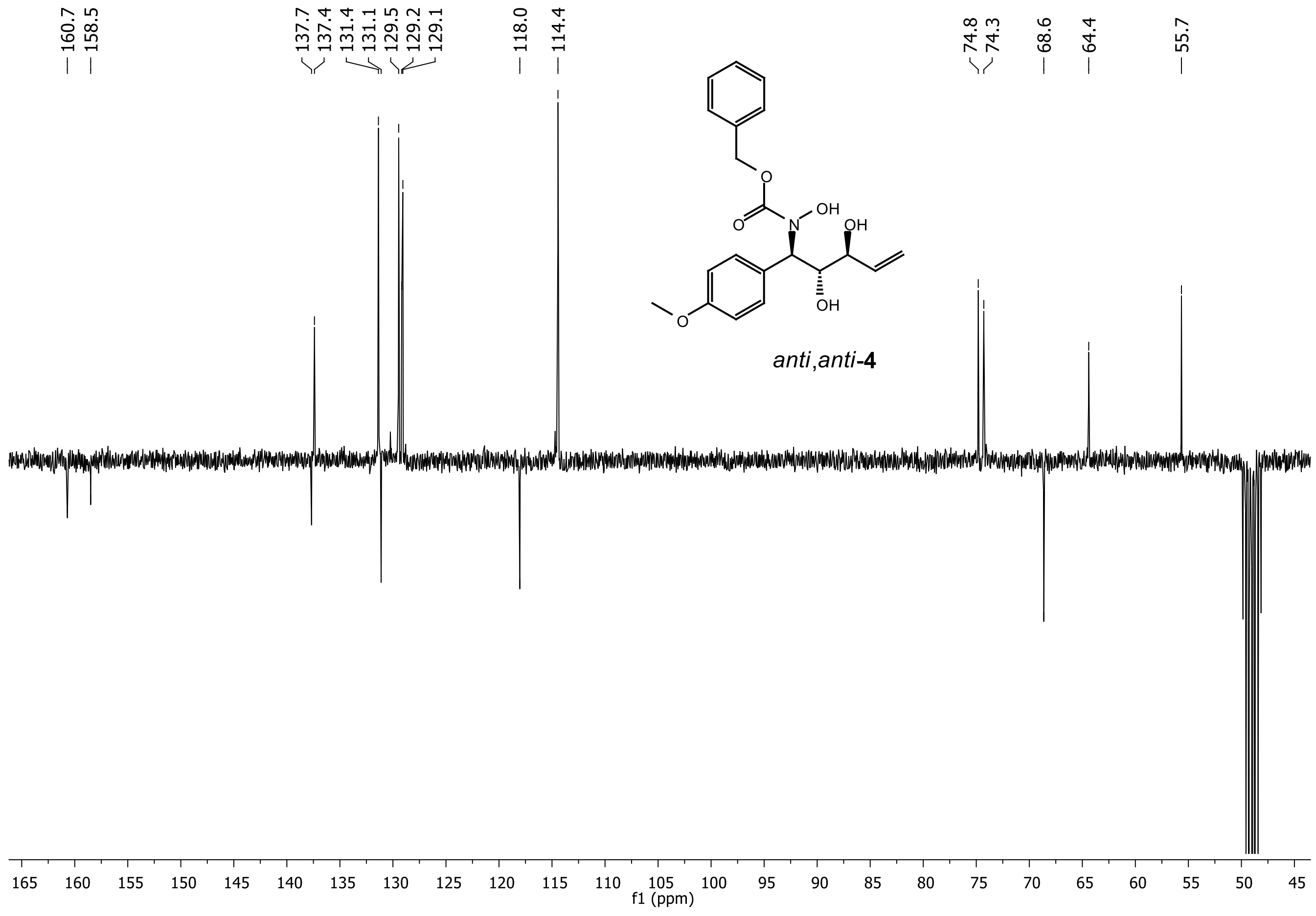
160.7
158.5
137.7
137.4
131.4
131.1
129.5
129.2
129.1
118.0
114.4
74.8
74.3
68.6
64.4
55.7
O
O
N
OH
OH
O
OH
anti,anti-4
165 160 155 150 145 140 135 130 125 120 115 110 105 100 95 90 85 80 75 70 65 60 55 50 45
f1 (ppm)

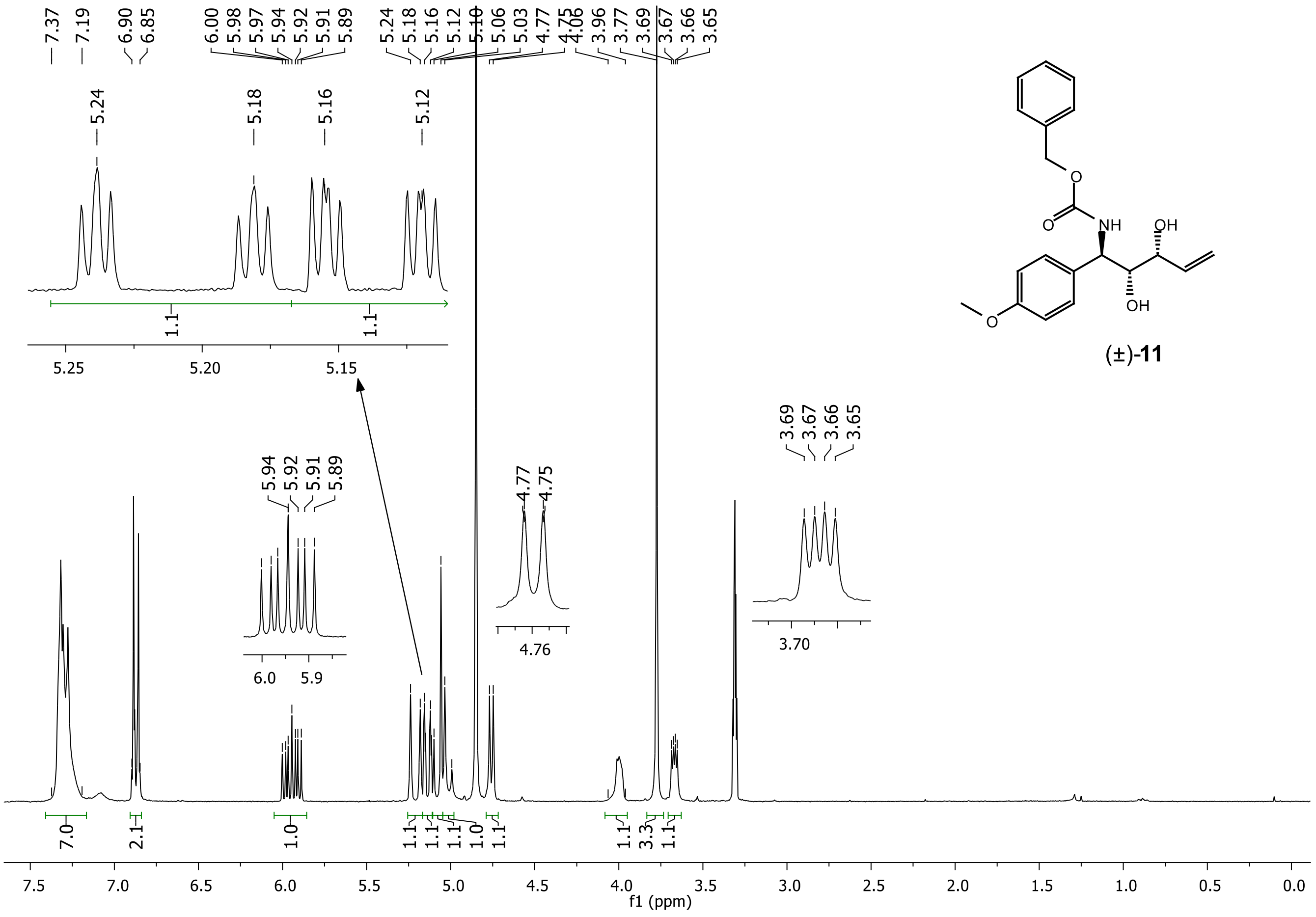
7.37
7.19
6.90
6.85
6.00
5.98
5.97
5.94
5.92
5.91
5.89
5.24
5.18
5.16
5.12
5.10
5.06
5.03
4.77
4.75
4.06
3.96
3.77
3.69
3.67
3.66
3.65
NH
OH
O
OH
f1 (ppm)

(±)-**11**

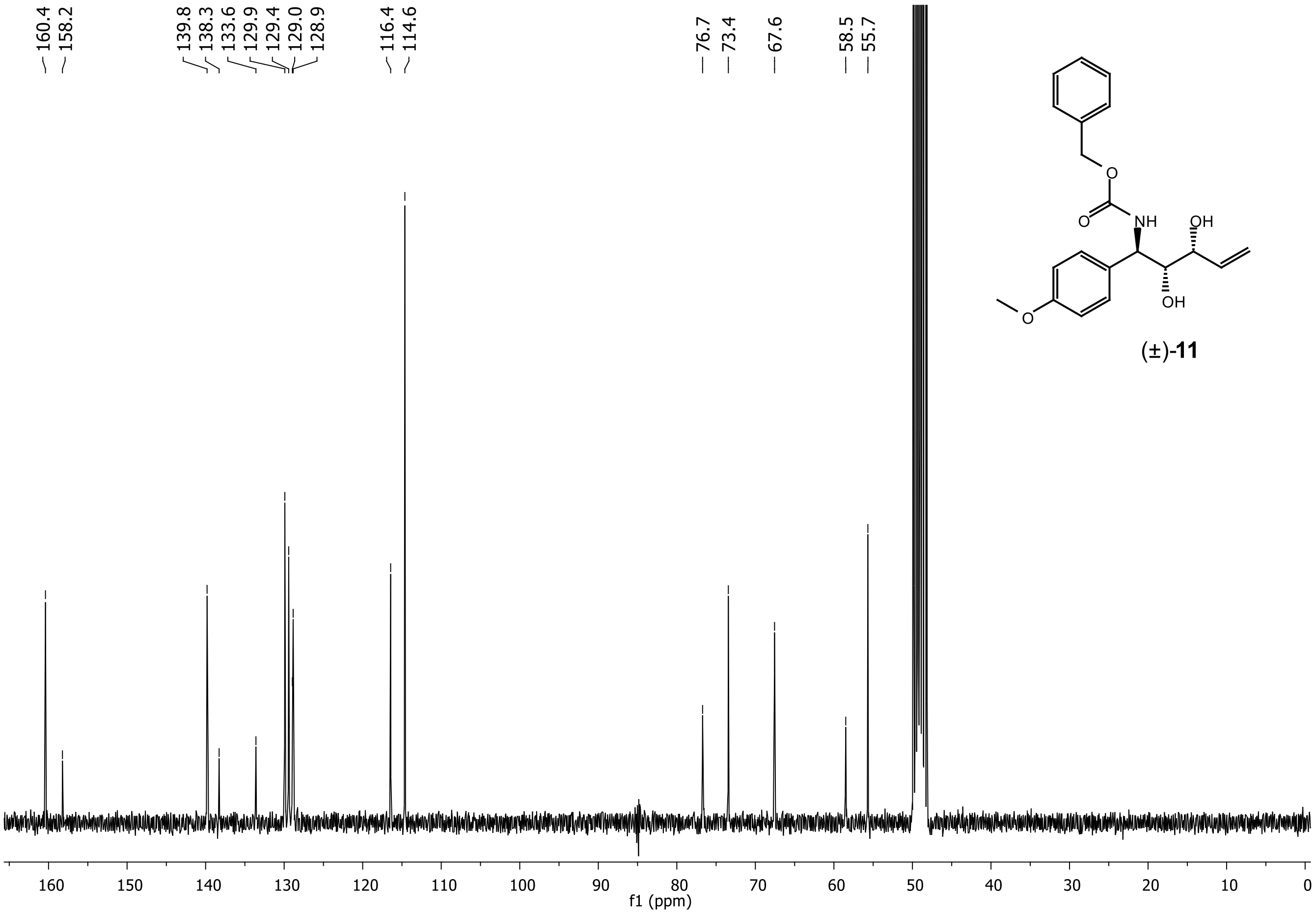
160.4
158.2
139.8
138.3
133.6
129.9
129.4
129.0
128.9
116.4
114.6
76.7
73.4
67.6
58.5
55.7
NH
OH
O
OH
f1 (ppm)

(±)-**11**

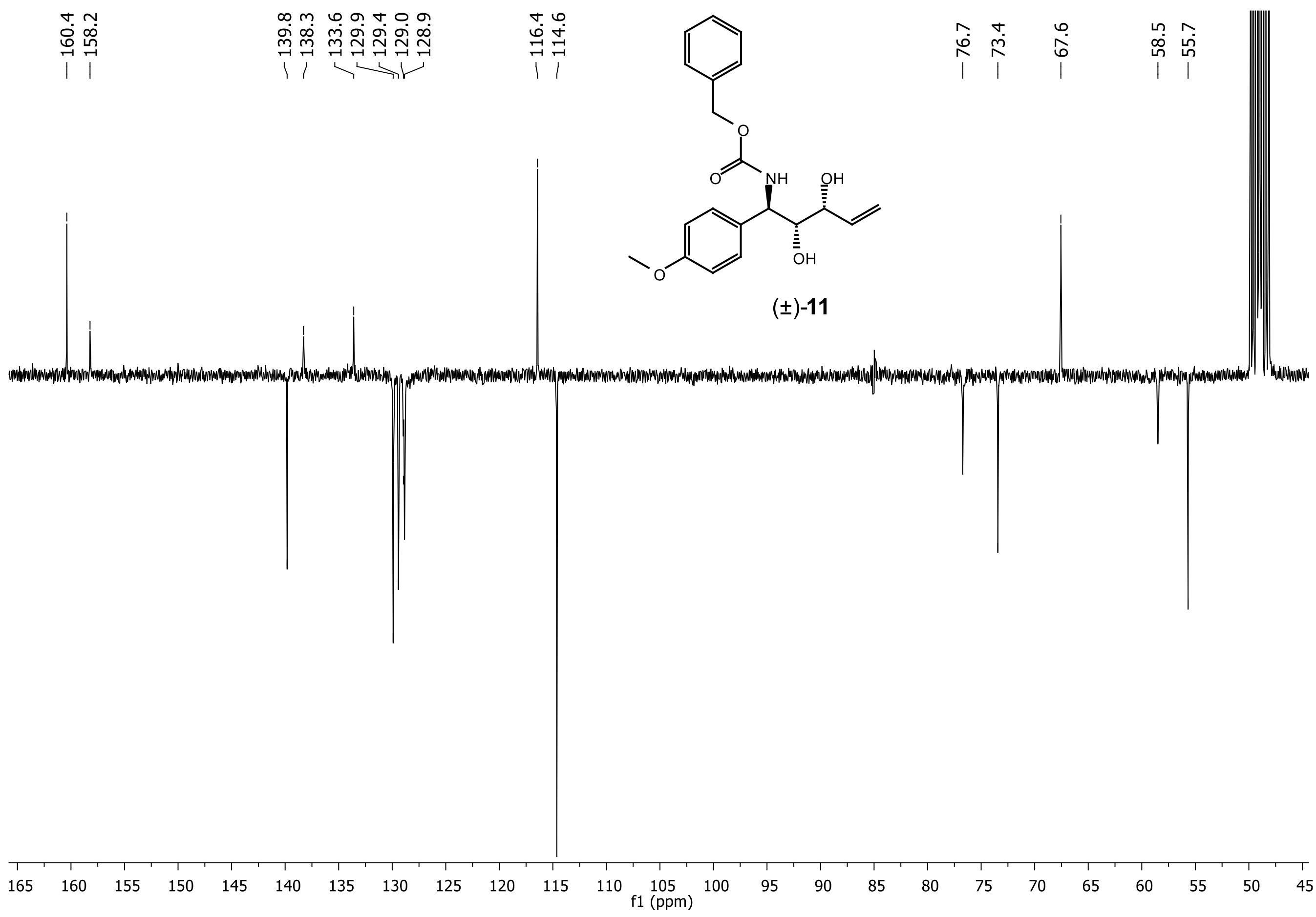
160.4
158.2
139.8
138.3
133.6
129.9
129.4
129.0
128.9
116.4
114.6
76.7
73.4
67.6
58.5
55.7
O
O
NH
OH
OH
O
(±)-11
165 160 155 150 145 140 135 130 125 120 115 110 105 100 95 90 85 80 75 70 65 60 55 50 45
f1 (ppm)

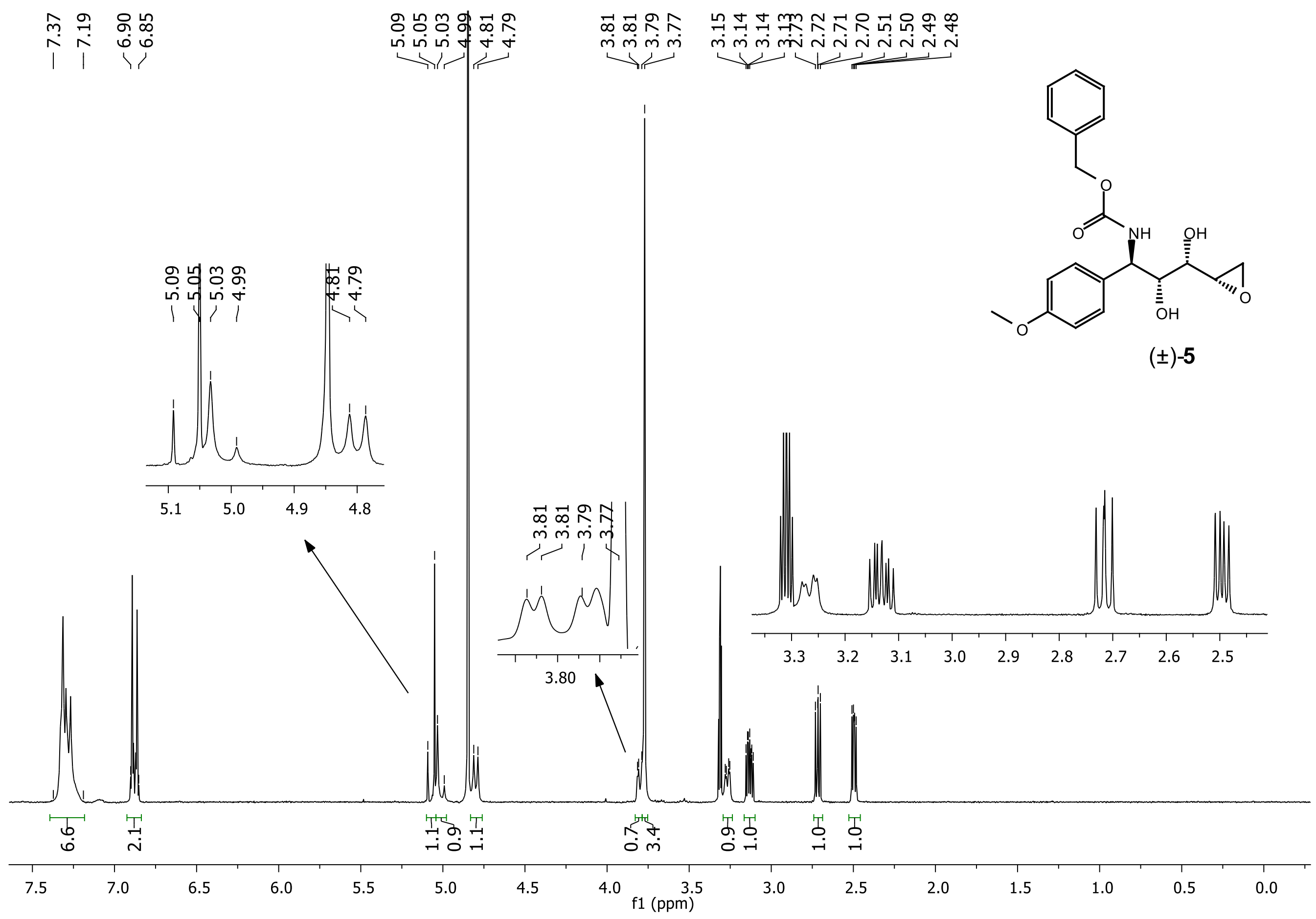
(±)-5
f1 (ppm)

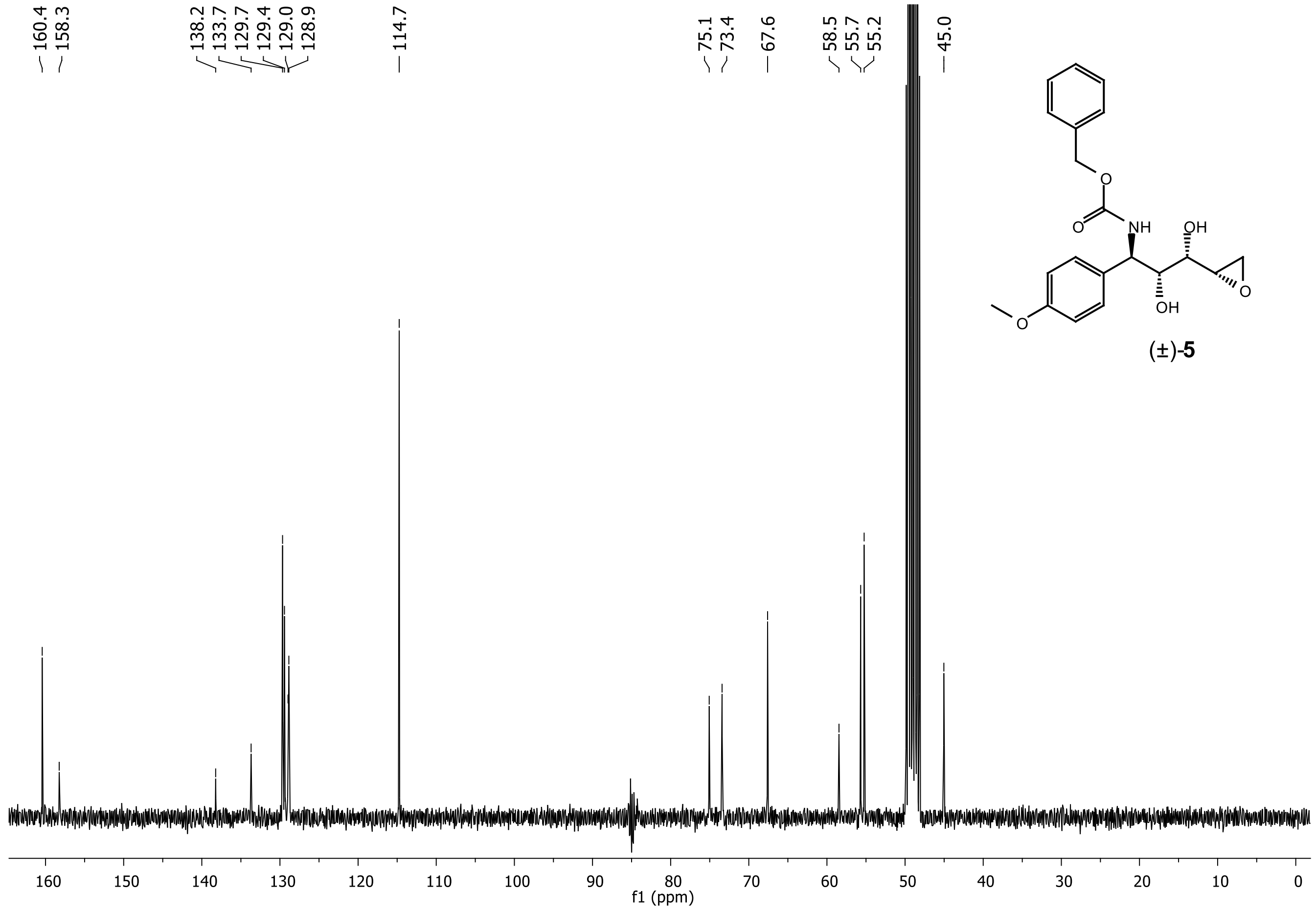
(±)-5
f1 (ppm)

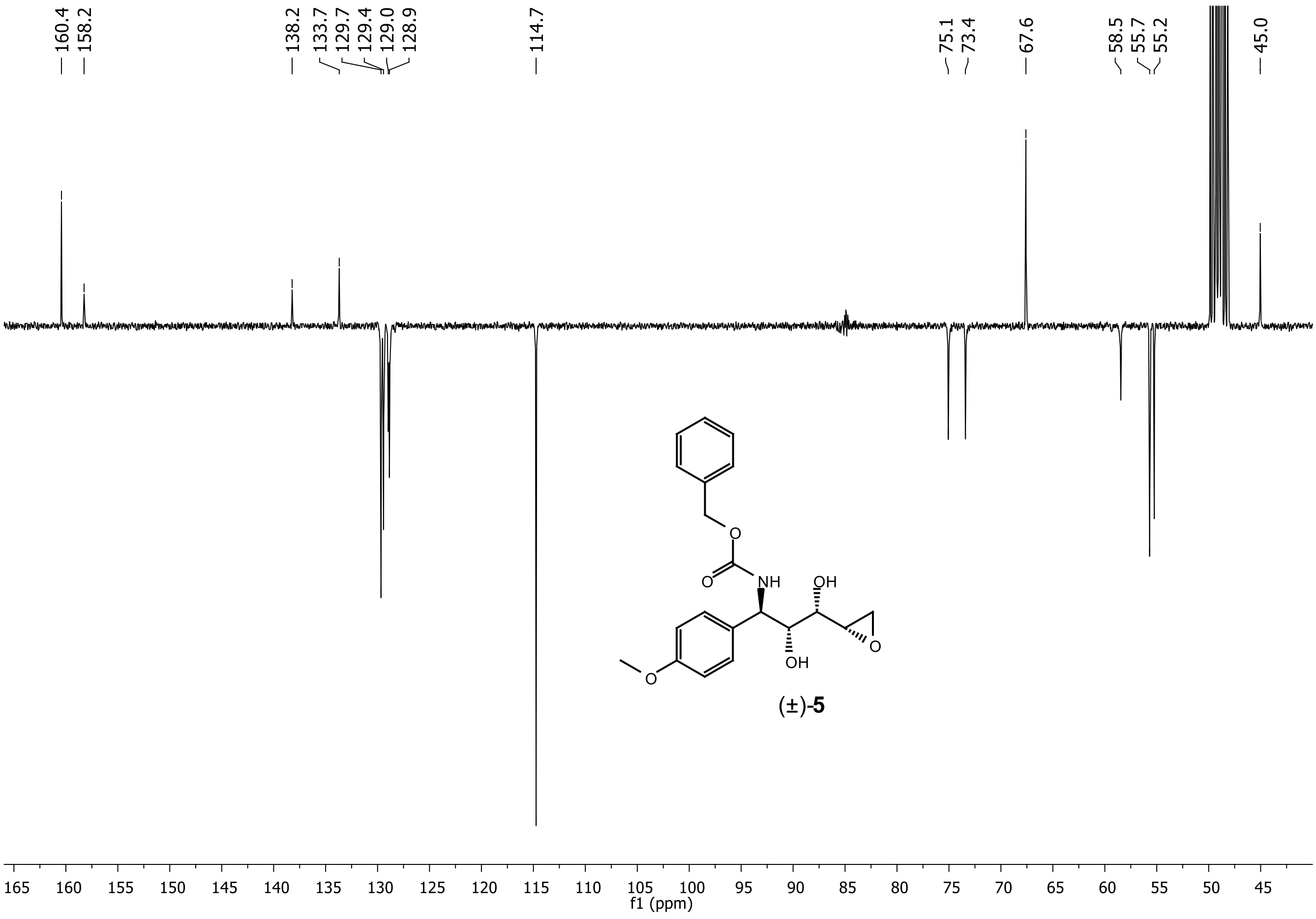
160.4
158.2
138.2
133.7
129.7
129.4
129.0
128.9
114.7
75.1
73.4
67.6
58.5
55.7
55.2
45.0
O
O
NH
OH
OH
O
O
(±)-5
165 160 155 150 145 140 135 130 125 120 115 110 105 100 95 90 85 80 75 70 65 60 55 50 45
f1 (ppm)

<u>Spectrum A</u>

an epoxidation with

$H_2O_2/12WO_3{\cdot}H_3PO_4xH_2O$

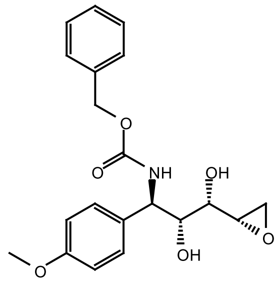


(±)-**5**

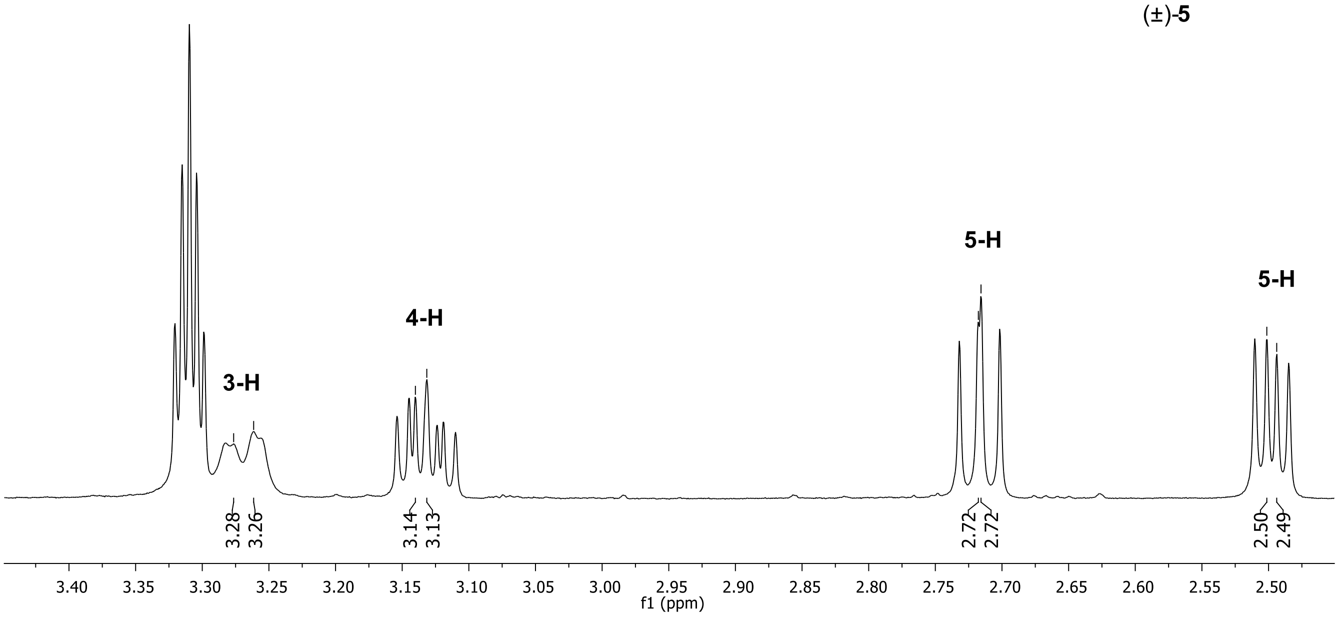


<u>Spectrum B</u>

an epoxidation with

*m*-CPBA

*anti*,*syn*,*syn*-**5**

(A)

*anti*,*syn*,*anti*-**5**

(B)

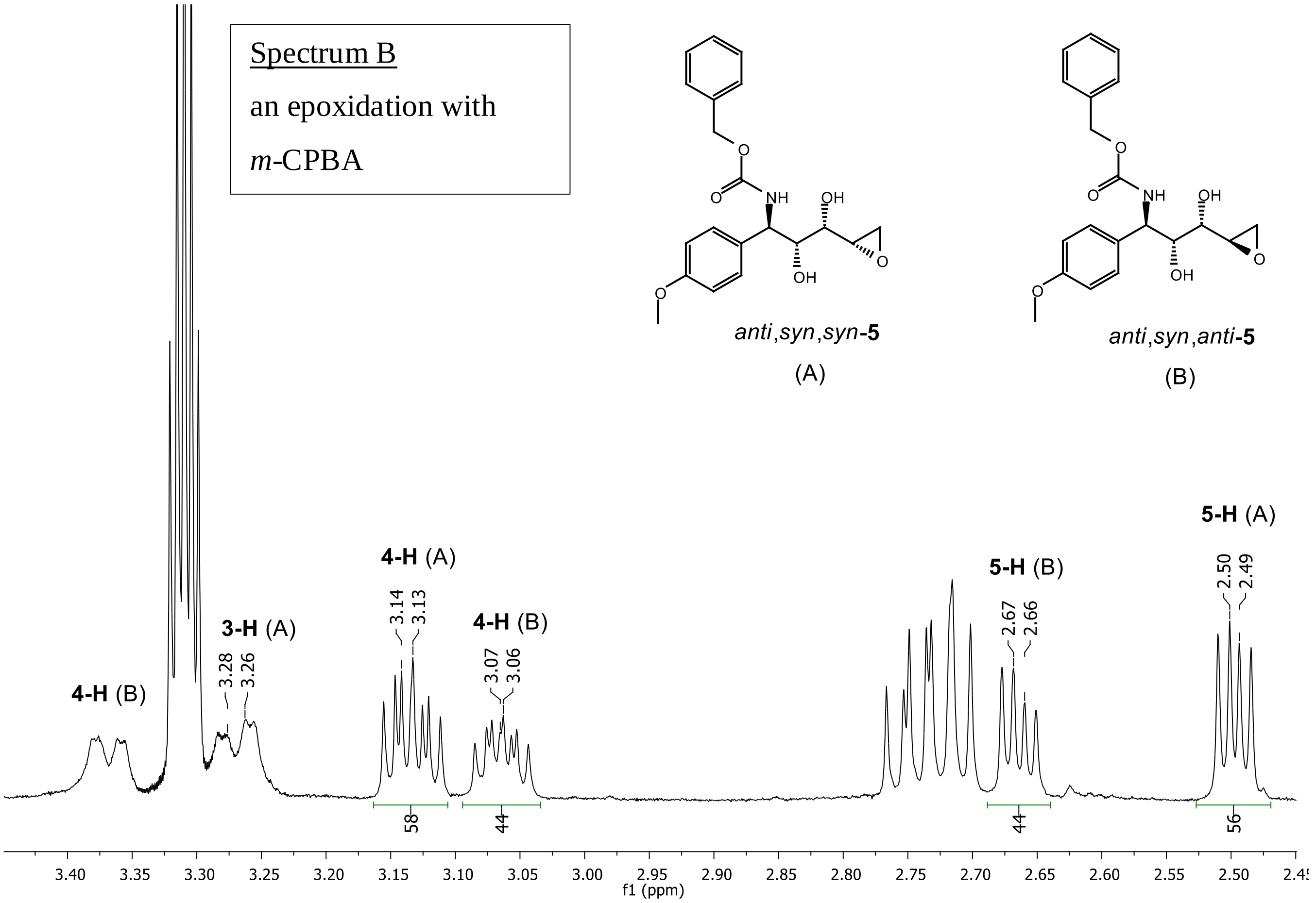

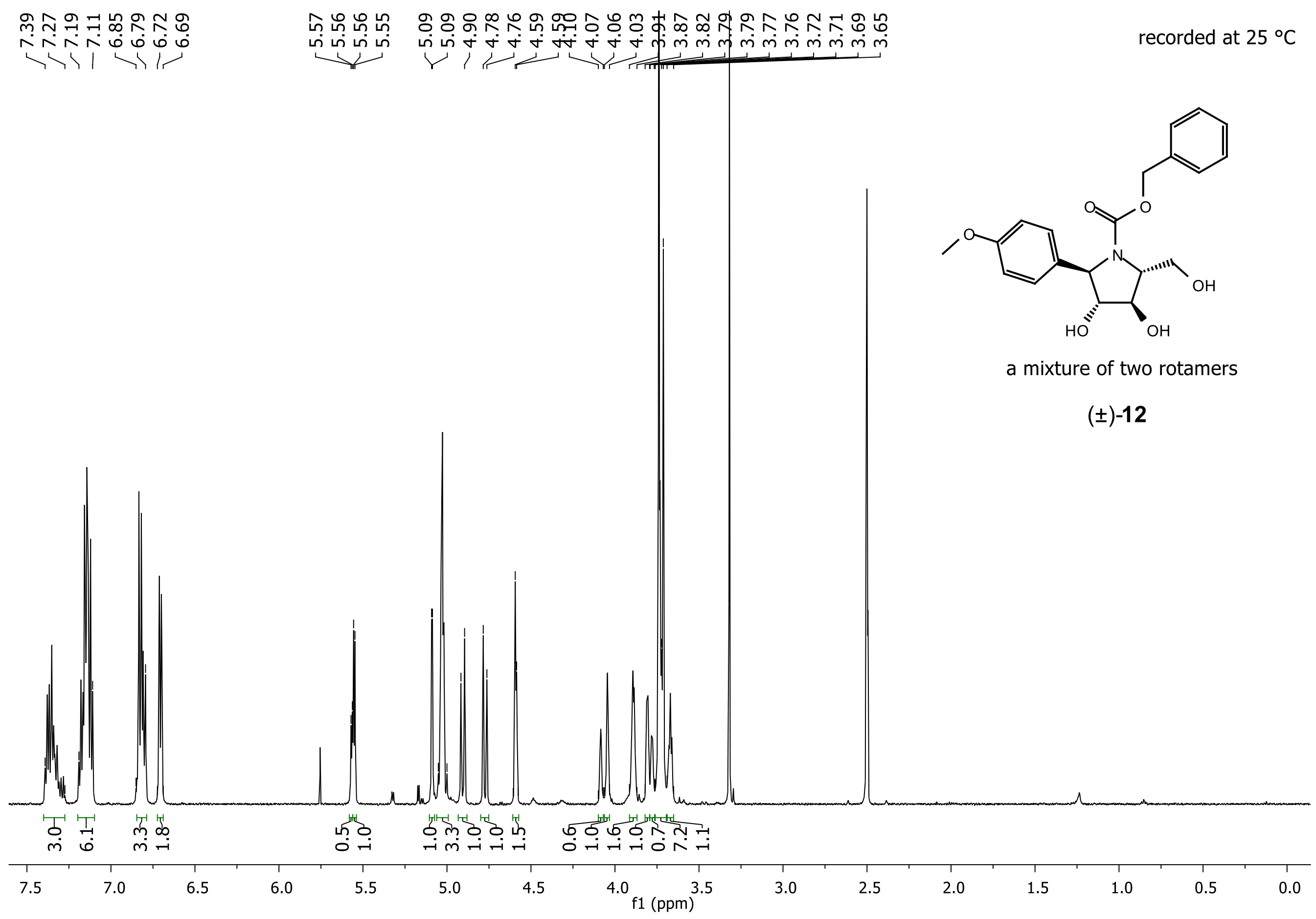
recorded at 25 °C
a mixture of two rotamers
(±)-12
f1 (ppm)

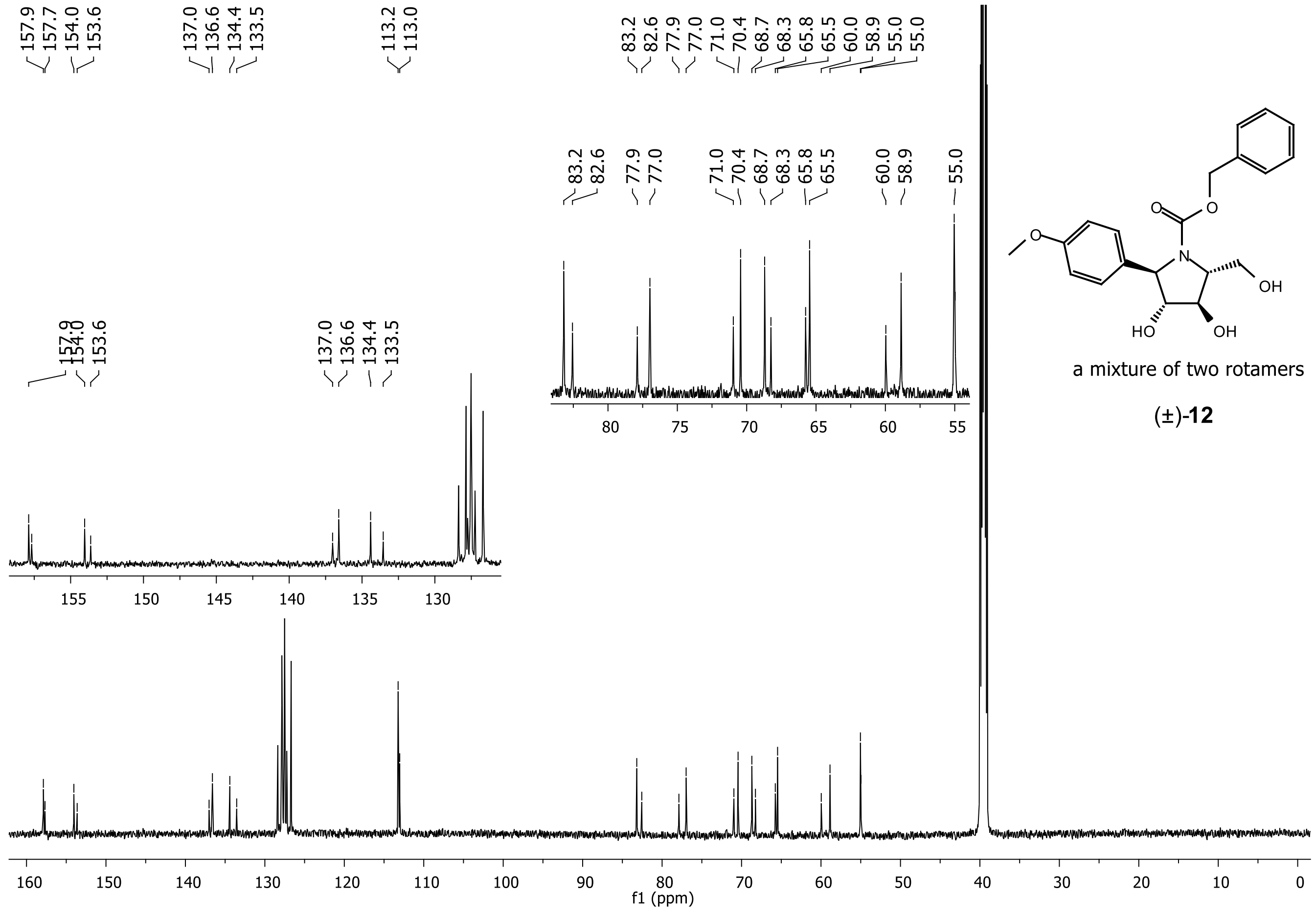
a mixture of two rotamers
(±)-12
f1 (ppm)

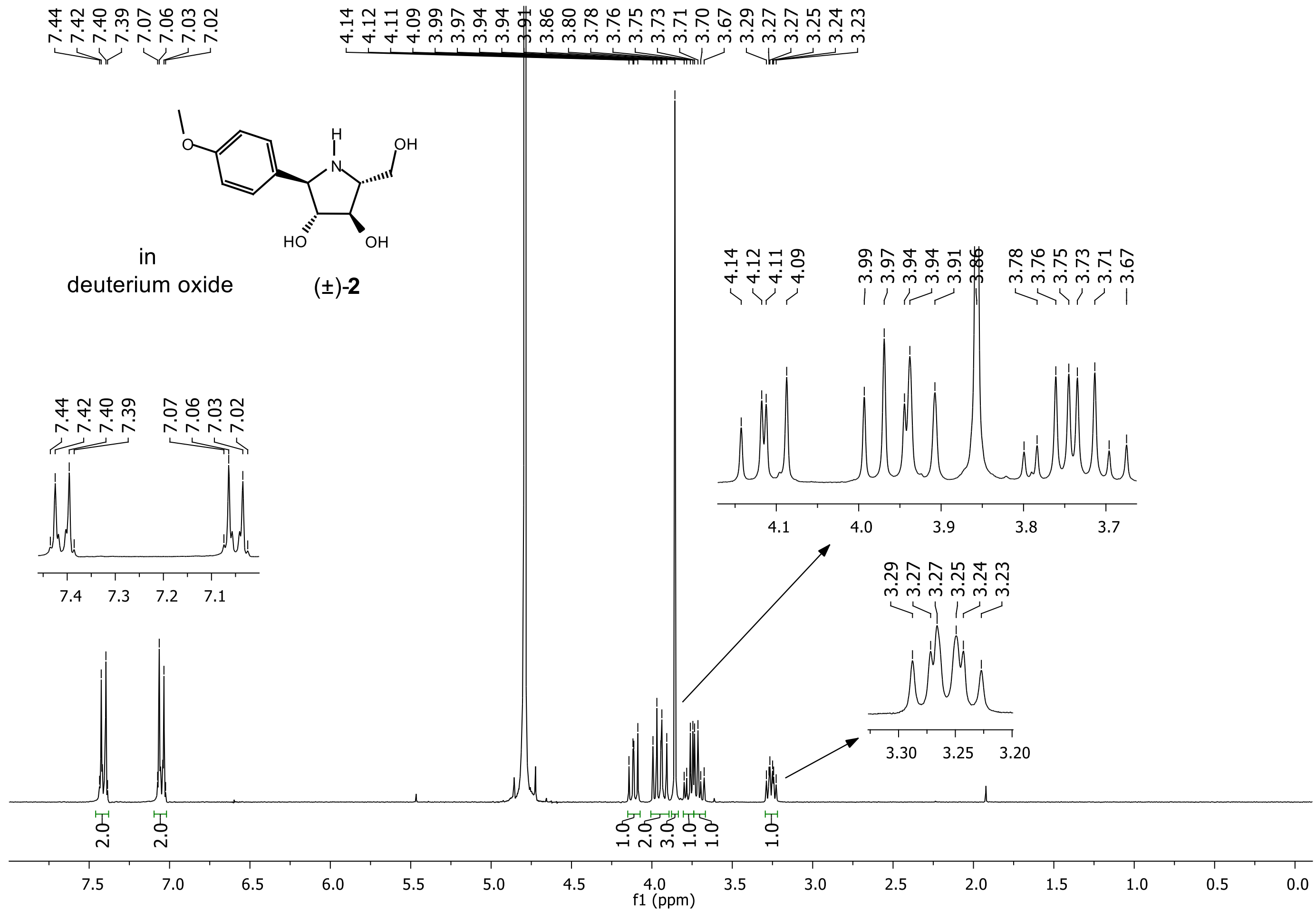
7.44 7.42 7.40 7.39 7.07 7.06 7.03 7.02
4.14 4.12 4.11 4.09 3.99 3.97 3.94 3.94 3.91 3.86 3.80 3.78 3.76 3.75 3.73 3.71 3.70 3.67 3.29 3.27 3.27 3.25 3.24 3.23
in
deuterium oxide
(±)-2
2.0
2.0
1.0
2.0
3.0
1.0
1.0
1.0
f1 (ppm)

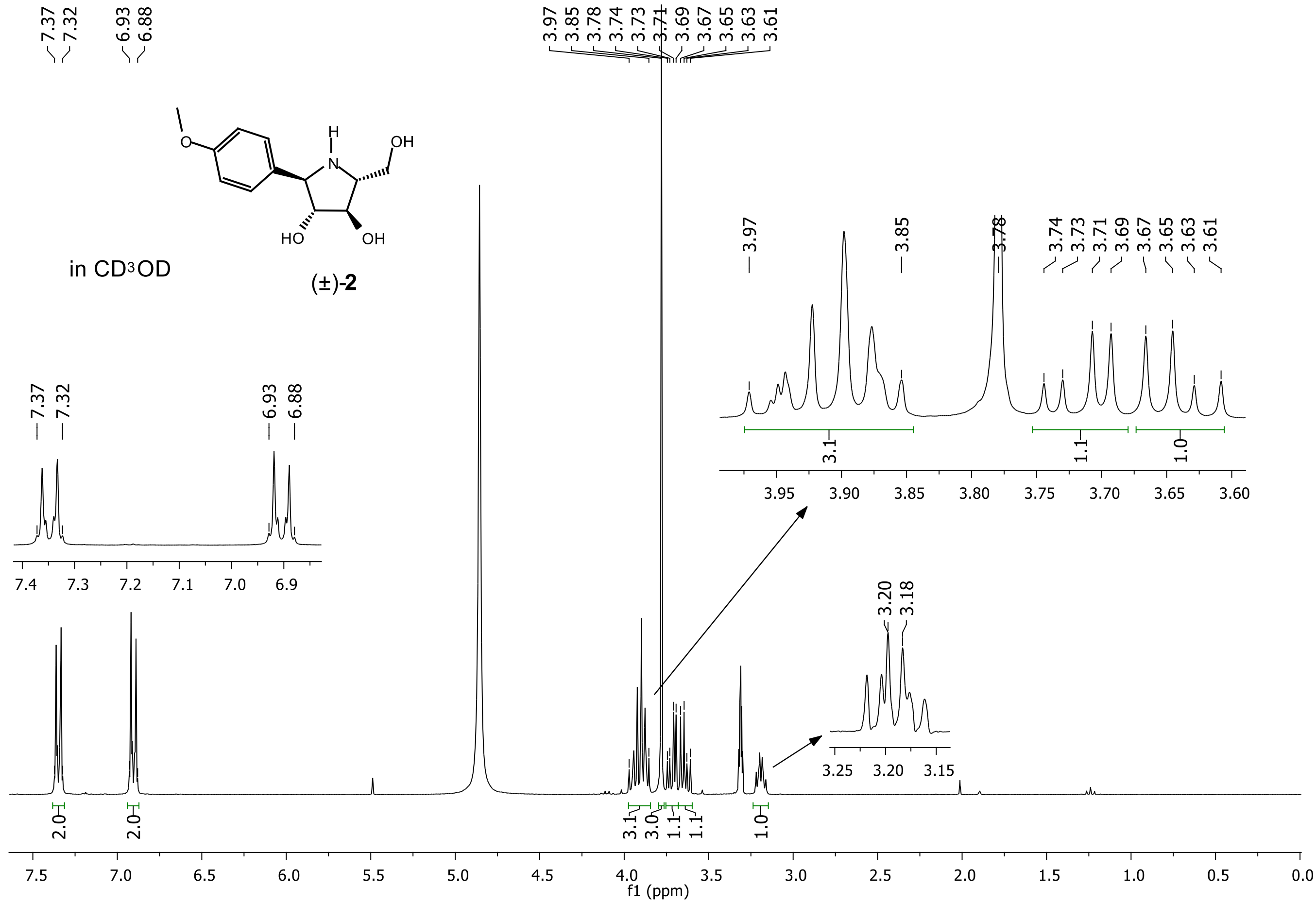
7.37 7.32 6.93 6.88
3.97 3.85 3.78 3.74 3.73 3.71 3.69 3.67 3.65 3.63 3.61
in CD3OD
(±)-2
3.20 3.18
2.0
2.0
3.1
3.0
1.1
1.1
1.0
f1 (ppm)

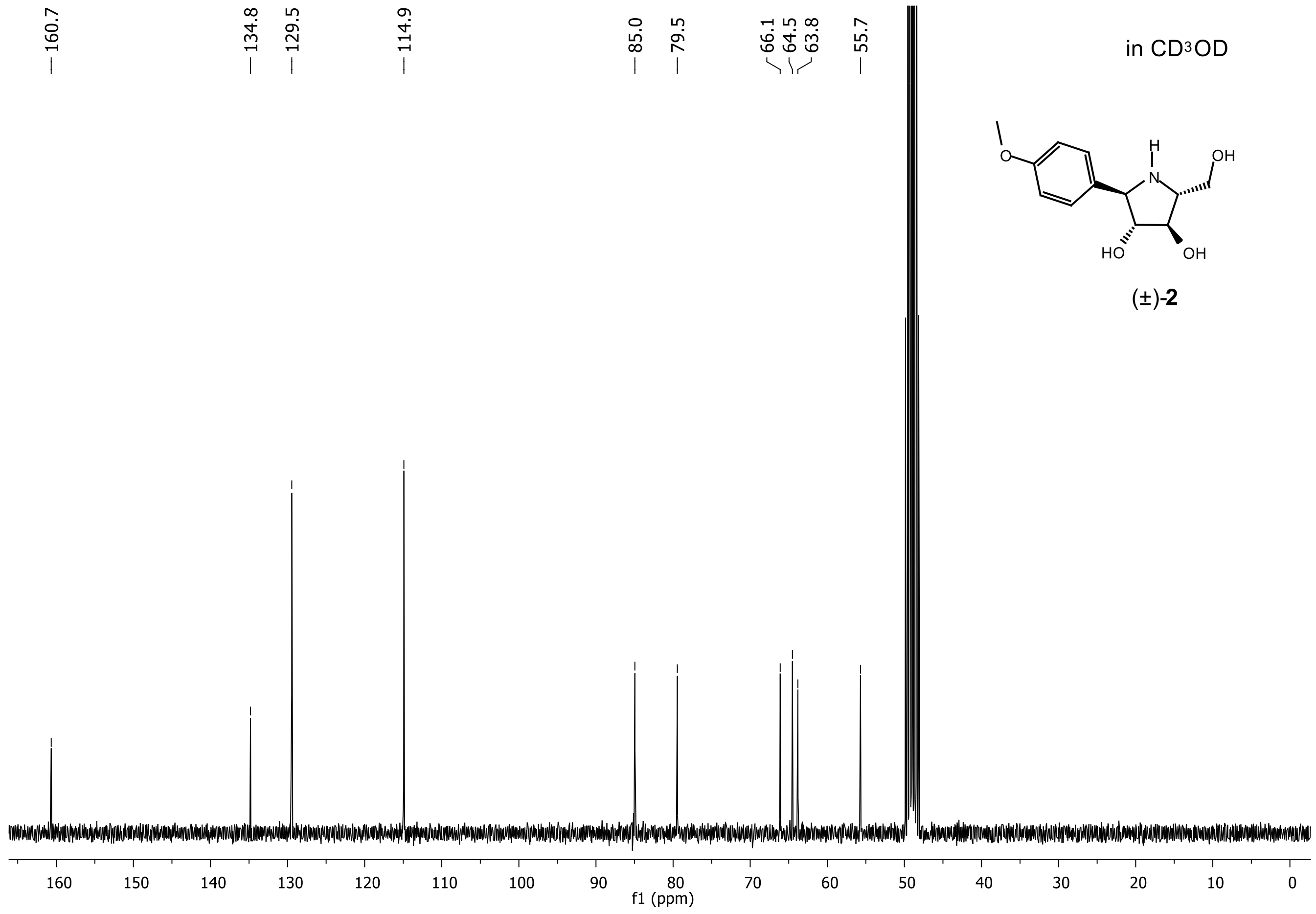
160.7
134.8
129.5
114.9
85.0
79.5
66.1
64.5
63.8
55.7
in CD3OD
H
N
OH
O
HO
OH
(±)-2
160
150
140
130
120
110
100
90
80
70
60
50
40
30
20
10
0
f1 (ppm)

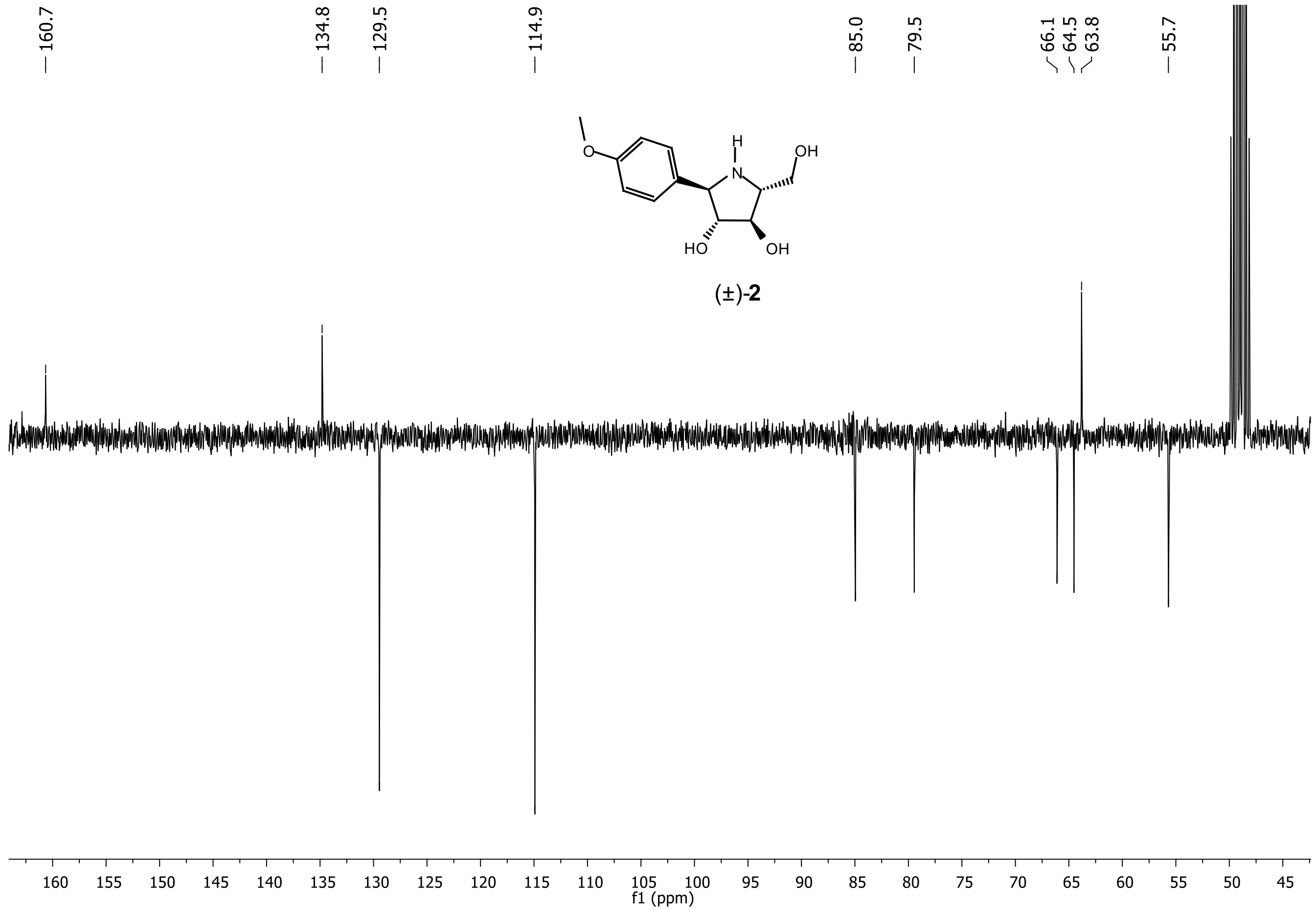
160.7
134.8
129.5
114.9
85.0
79.5
66.1
64.5
63.8
55.7
H
N
OH
O
HO
OH
(±)-2
160
155
150
145
140
135
130
125
120
115
110
105
100
95
90
85
80
75
70
65
60
55
50
45
f1 (ppm)

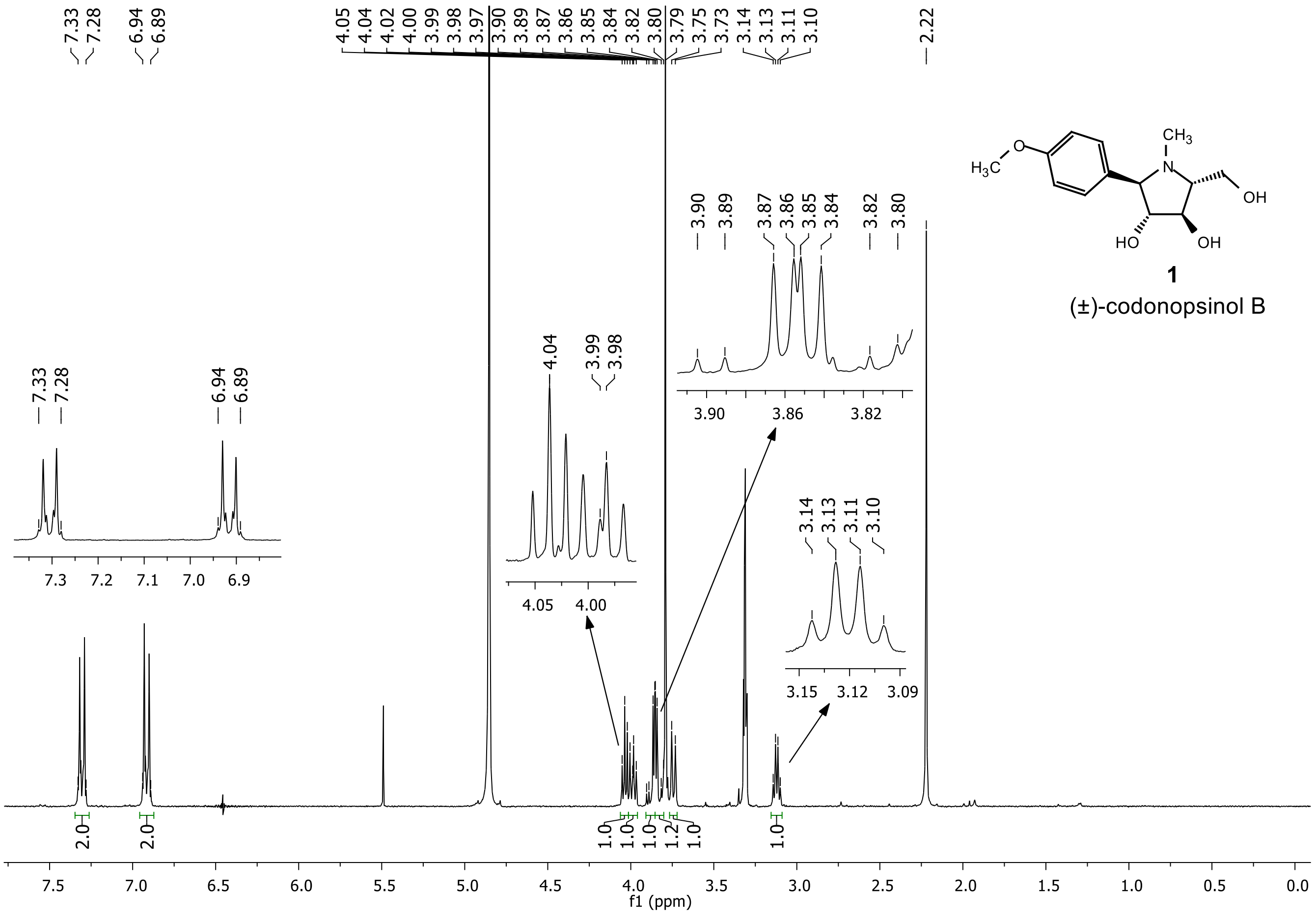
H3C
O
CH3
N
OH
HO
OH
1
(±)-codonopsinol B
f1 (ppm)

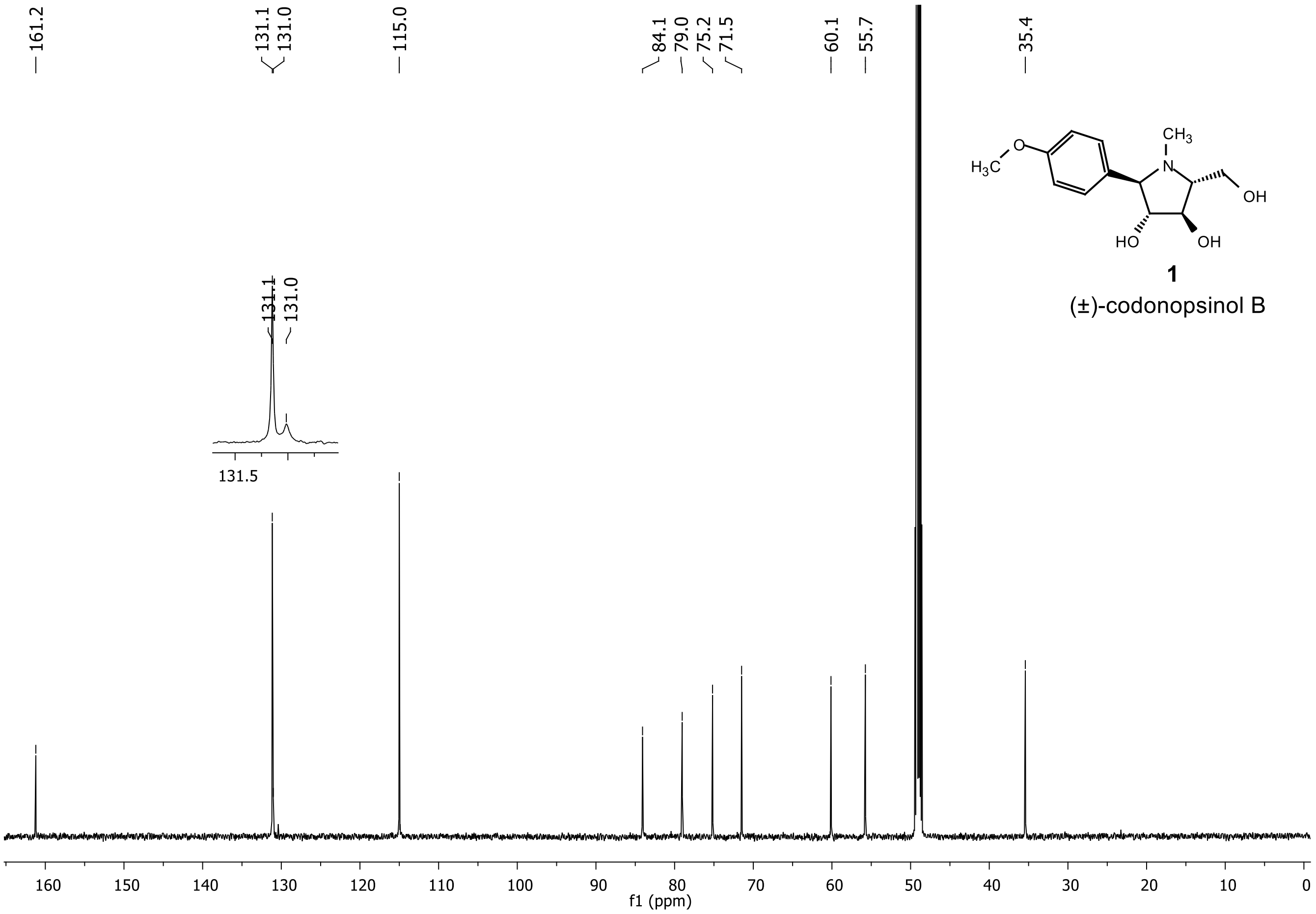
H3C
O
CH3
N
OH
HO
OH
1
(±)-codonopsinol B
f1 (ppm)

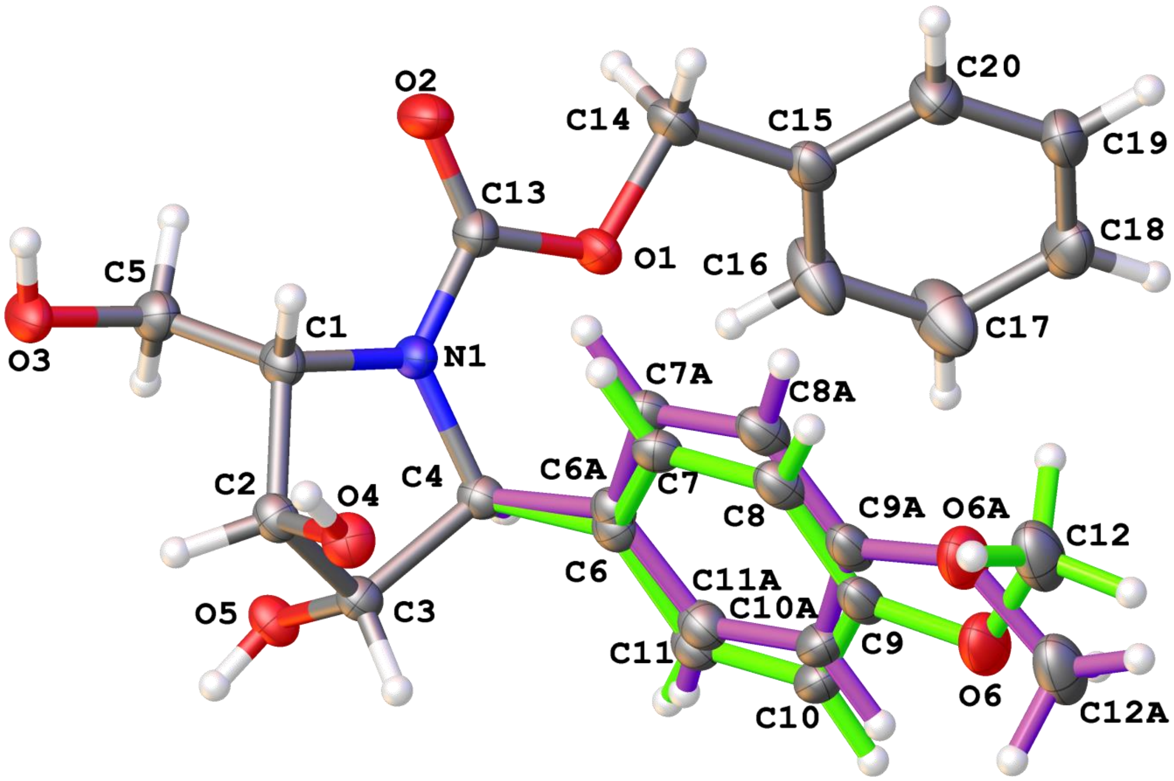


**Figure S1:** The ORTEP-like style drawn of IAM model of **12** with the thermal ellipsoids shown at a 50% probability level. The green and purple lines represent the main and minor part of the disorder.

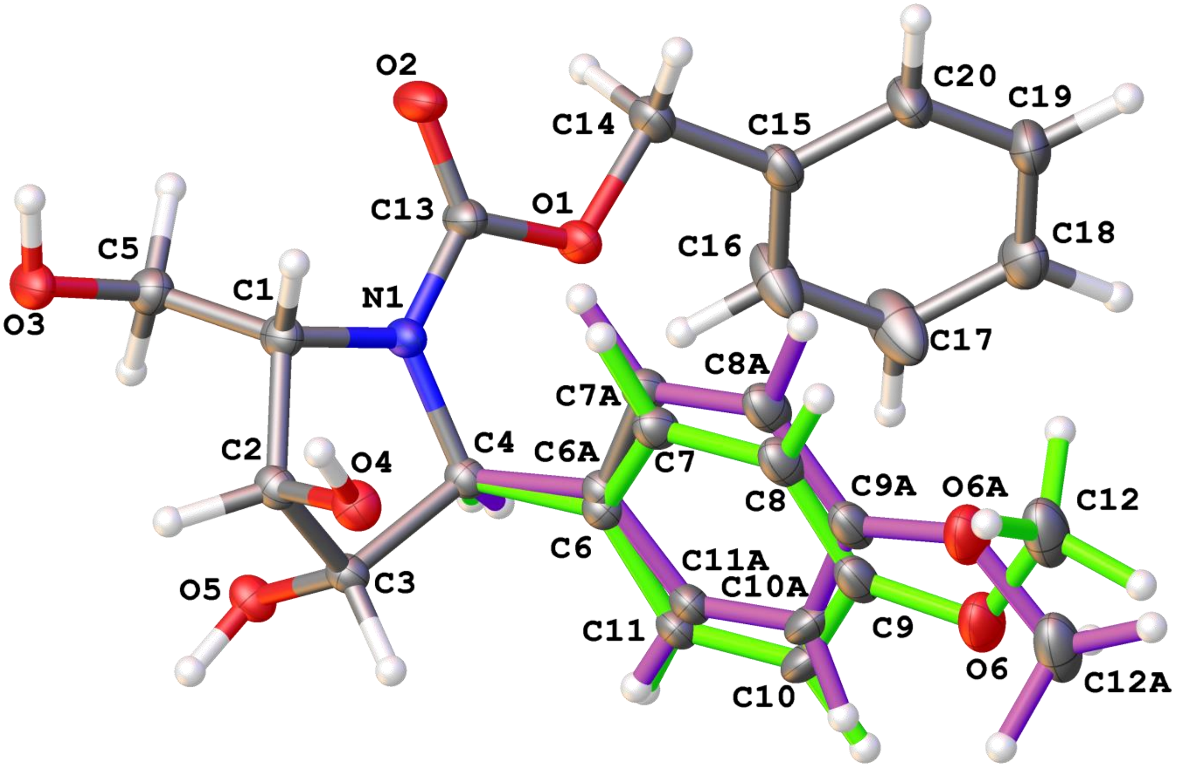


**Figure S2:** The ORTEP-like style drawn of HAR model of **12** with the thermal ellipsoids shown at a 50% probability level. The green and purple lines represent the main and minor part of the disorder.

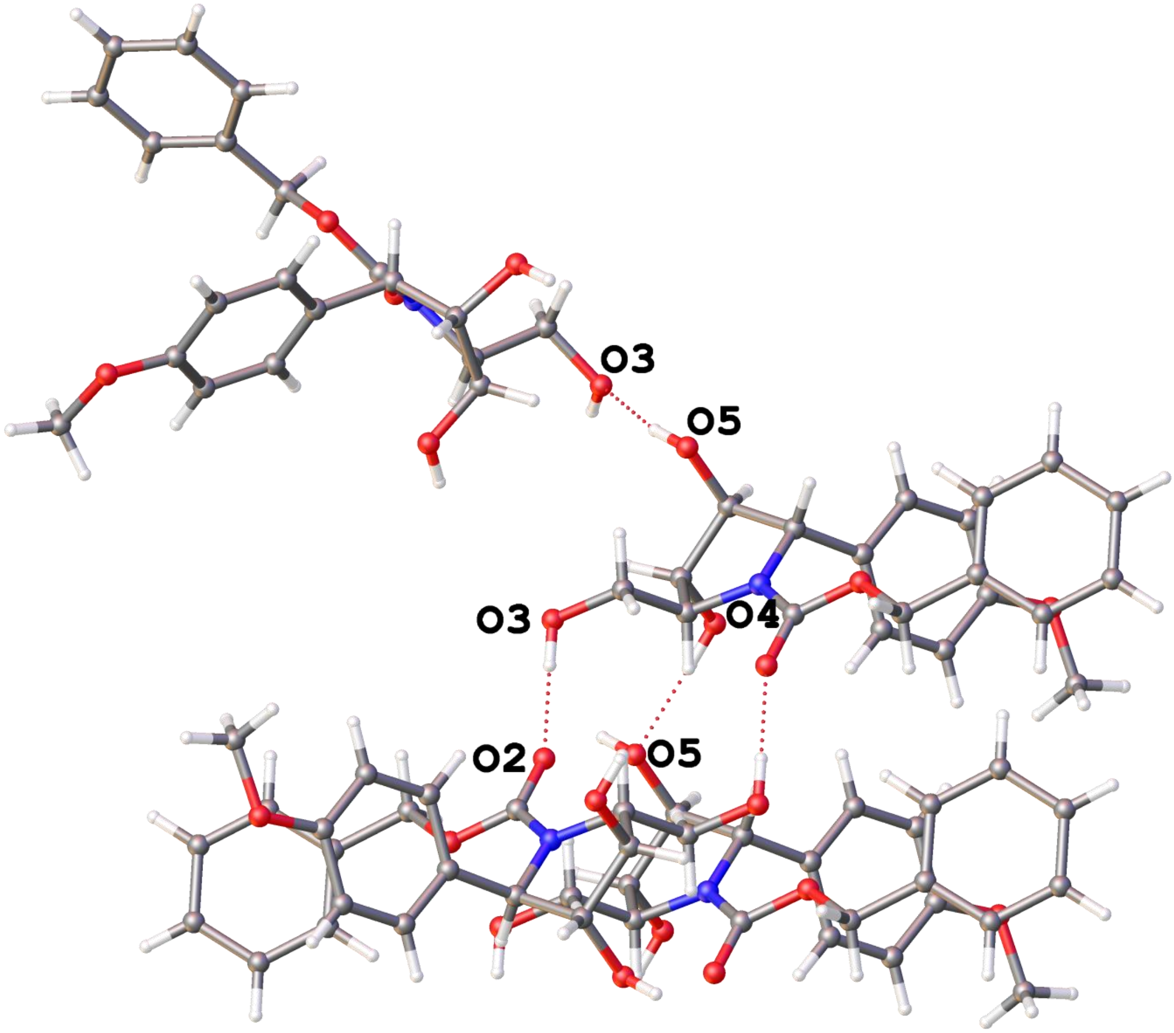


**Figure S3:** The O–H···O hydrogen bonds in the crystal structure of **12**. The minor part of disorder has been removed for clarity.

**Table S1:** Summarization of $IC_{50}$ values for compounds (±)-**1** and (±)-**2**.

| **Tested compound** | **Cell line** | **The concentration range (μM)** | **$IC_{50}$ (μM)** treatment 24 h | **$IC_{50}$ (μM)** treatment 48 h | **$IC_{50}$ (μM)** treatment 72 h |
|---|---|---|---|---|---|
| **1** | U87-MG | 1–1000 | $> 1000$ | $> 1000$ | $> 1000$ |
| **2** | U87-MG | 1–1000 | $> 1000$ | $> 1000$ | $> 1000$ |
| **1** | HK-2 | 1–1000 | $> 1000$ | $> 1000$ | $> 1000$ |
| **2** | HK-2 | 1–1000 | $> 1000$ | $> 1000$ | $> 1000$ |
| **1** | HepG2 | 1–1000 | $> 1000$ | $> 1000$ | $> 1000$ |
| **2** | HepG2 | 1–1000 | $> 1000$ | $> 1000$ | $> 1000$ |
| **1** | JEG-3 | 1–1000 | $> 1000$ | $> 1000$ | –* |
| **2** | JEG-3 | 1–1000 | $> 1000$ | $> 1000$ | –* |

*Note:* The viability of JEG-3 cell line was not tested after 72 hours treatment of compounds.*

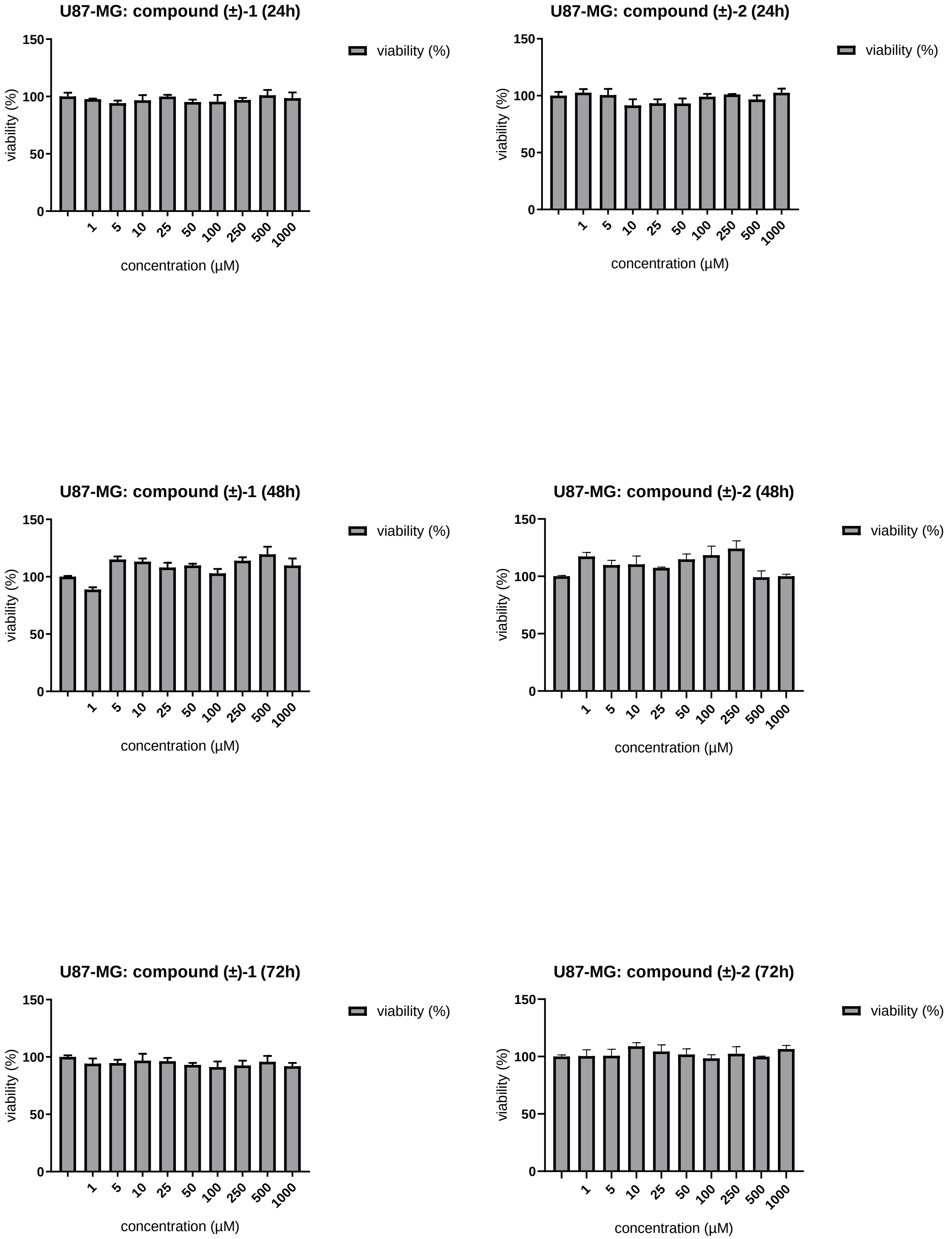


**Figure S4:** Antiproliferative effect of the tested compounds (±)-**1** and (±)-**2** on U87-MG cells after 24, 48, and 72 h treatment.

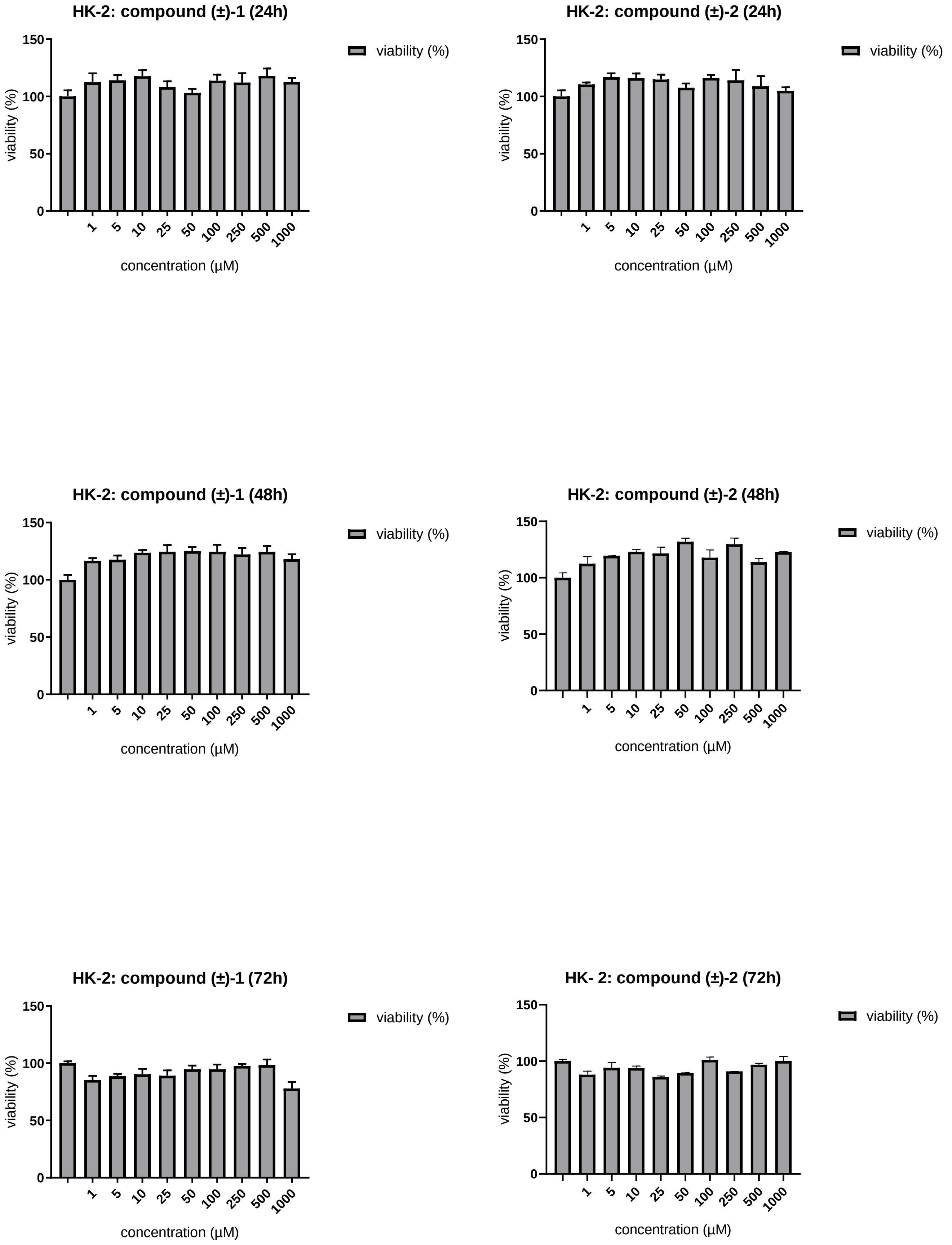


**Figure S5:** Antiproliferative effect of the tested (±)-**1** and (±)-**2** compounds on HK-2 cells after 24, 48, and 72 h treatment.

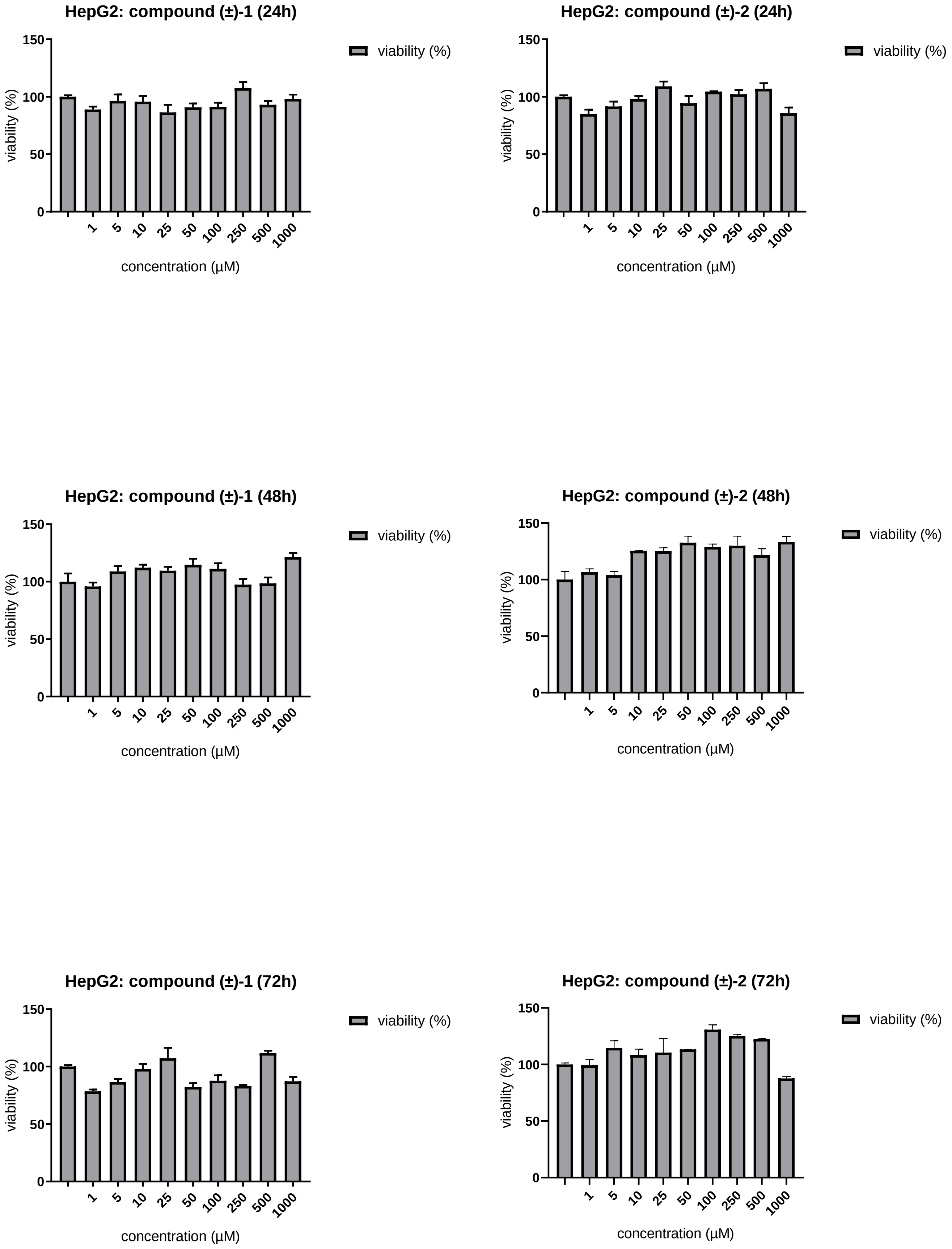


**Figure S6:** Antiproliferative effect of the tested compounds (±)-**1** and (±)-**2** on HepG2 cells after 24, 48, and 72 h treatment.

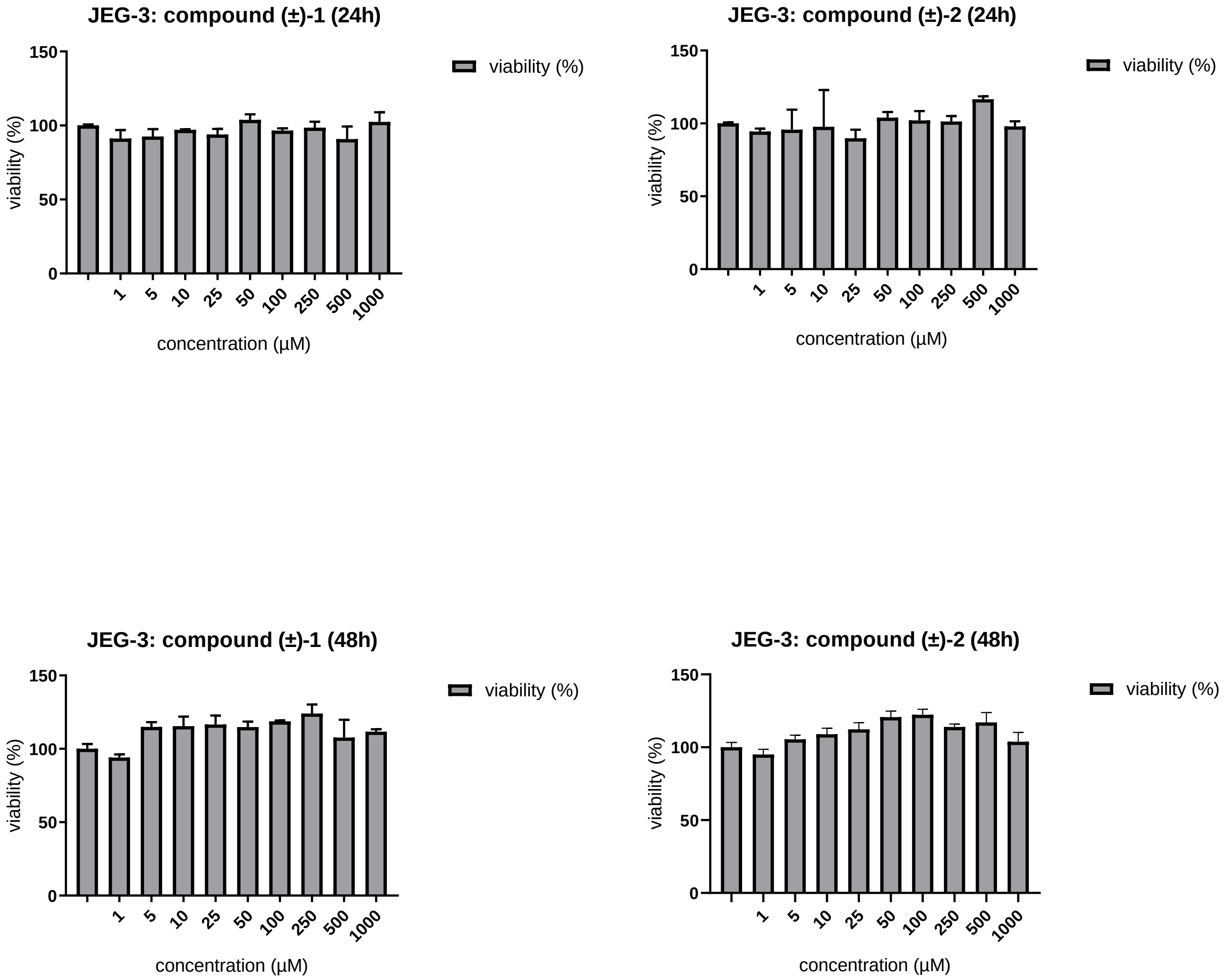


**Figure S7:** Antiproliferative effect of the tested compounds (±)-**1** and (±)-**2** on JEG-3 cells after 24 or 48 h treatment.

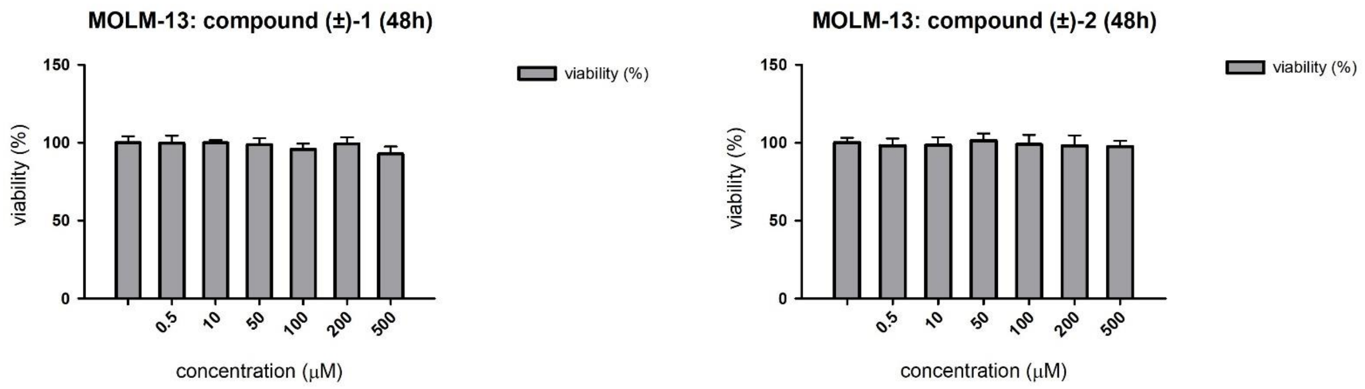


**Figure S8:** Antiproliferative effect of the tested compounds (±)-**1** and (±)-**2** on MOLM-13 cells after 48 h treatment.

## References


1. Katritzky, A. R.; Avan, I.; Tala, S. R. *J. Org. Chem.* **2009**, *74*, 8690–8694. doi:10.1021/jo901612j
2. Lai, J.; Sayalero, S.; Ferrali, A.; Osorio-Planes, L.; Bravo, F.; Rodríguez-Escrich, C.; Pericàs, M. A. *Adv. Synth. Catal.* **2018**, *360*, 2914–2924. doi:10.1002/adsc.201800572
3. Ďurina, L.; Malatinský, T.; Moncol, J.; Záborský, O.; Fischer, R. *Synthesis* **2021**, 53, 688–698. doi:10.1055/s-0040-1706543
4. Tsou, E.-L.; Chen, S.-Y.; Yang, M.-H.; Wang, S.-C.; Cheng, T.-R. R.; Cheng, W.-C. *Bioorg. Med. Chem.* **2008**, *16*, 10198–10204. doi:10.1016/j.bmc.2008.10.063
5. Wakana, D.; Kawahara, N.; Goda, Y. *Chem. Pharm. Bull.* **2013**, *61,* 1315–1317. doi:10.1248/cpb.c13-00516
6. Burla, M. C.; Caliandro, R.; Carrozzini, B.; Cascarano, G. L.; Cuocci, C.; Giacovazzo, C.; Mallamo, M.; Mazzone, A. G.; Polidori, G. *J. Appl. Crystallogr.* **2015**, *48*, 306–309. doi:10.1107/S1600576715001132
7. Sheldrick, G. M. *Acta Crystallogr. Sect. C* **2015**, *71*, 3–8. doi:10.1107/S2053229614024218
8. Neese, F. *Comput. Mol. Sci.* **2012**, *2*, 73–78. doi:10.1002/wcms.81
9. Neese, F. *Comput. Mol. Sci.* **2018**, *8*, e1327. doi:10.1002/wcms.1327
10. Bourhis, L. J.; Dolomanov, O. V.; Gildea, R. J.; Howard, J. A. K.; Puschmann, H. *Acta Crystallogr. Sect. A* **2015**, *71*, 59–75. doi:10.1107/S2053273314022207
11. Kleemiss, F.; Dolomanov, O. V.; Bodensteiner, M.; Peyerimhoff, N.; Midgley, L.; Bourhis, L. J.; Genomi, A.; Malaspina, L. A.; Jayatilaka, D.; Spencer, J. L.; White, F.; Grundkötter-Stock, B.; Steinhauer, S.; Lentz, D.; Puschmann, H.; Grabowsky, S. *Chem. Sci.* **2021**, *12*, 1675–1692. doi:10.1039/D0SC05526C
12. Dolomanov, O. V.; Bourhis, L. J.; Gildea, R. J.; Howard, J. A. K.; Puschmann, H. *J. Appl. Crystallogr.* **2009**, *42*, 339–341. doi:10.1107/S0021889808042726
